# Formation of Glycosyl Trichloroacetamides from Trichloroacetimidate Donors Occurs through an Intermolecular Aglycon Transfer Reaction

**DOI:** 10.1021/acs.orglett.3c02196

**Published:** 2023-08-14

**Authors:** Koen N. A. van de Vrande, Dmitri V. Filippov, Jeroen D. C. Codée

**Affiliations:** Leiden Institute of Chemistry, Leiden University, Einsteinweg 55, 2333 CC Leiden, Netherlands

## Abstract

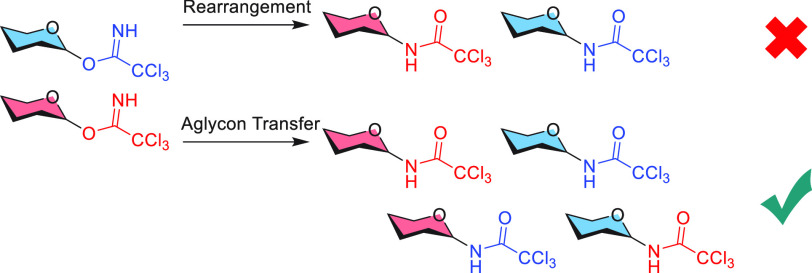

To probe the reaction mechanism, underlying the rearrangement of oft-used trichloroacetimidate glycosyl donors into the corresponding anomeric trichloroacetamides, we have used a combination of ^13^C- and ^15^N-labeled glycosyl trichloroacetimidate donors in a series of crossover experiments. These unambiguously show that trichloroacetamides are formed via an intermolecular aglycon transfer mechanism. This insight enables the design of more effective glycosylation protocols, preventing the formation of dead-end side products.

Glycosyl imidates have been one of the most popular glycosylating agents^1,2^ ever since their first conception by Sinaÿ and co-workers^3,4^ and the introduction of trichloroacetimidates by Schmidt and Michel.^5^ Trichloroacetimidates have been applied in many ground-breaking syntheses of biologically relevant oligosaccharides and have been used on an industrial scale, as exemplified by the multi-kilogram scale production Arixtra, the synthetic heparin-type pentasaccharide anticoagulant.^6^ Their popularity stems from the fact that they can be easily prepared from the corresponding lactol precursors and trichloroacetonitrile and rapidly activated using a catalytic amount of (Lewis) acid. The high reactivity of trichloroacetimidates however may lead to side reactions under glycosylation conditions, and the formation of donor-derived trichloroacetamides has been observed on many occasions, necessitating the use of an excess of expensive building blocks (see Scheme 1A).^7^ The formation of the amide side product is often referred to as a rearrangement reaction, suggesting that it takes place through a unimolecular reaction.^7−10^ To prevent the formation of the trichloroacetamide side product, Schmidt and Toepfer have introduced the “inverse glycosylation procedure”. In this procedure, the acceptor and activator are premixed before the (slow) addition of the donor as Schmidt and Toepfer reasoned that the formation of an “acceptor–activator complex” in the absence of donor would prevent donor decomposition.^8^

**Scheme 1:**
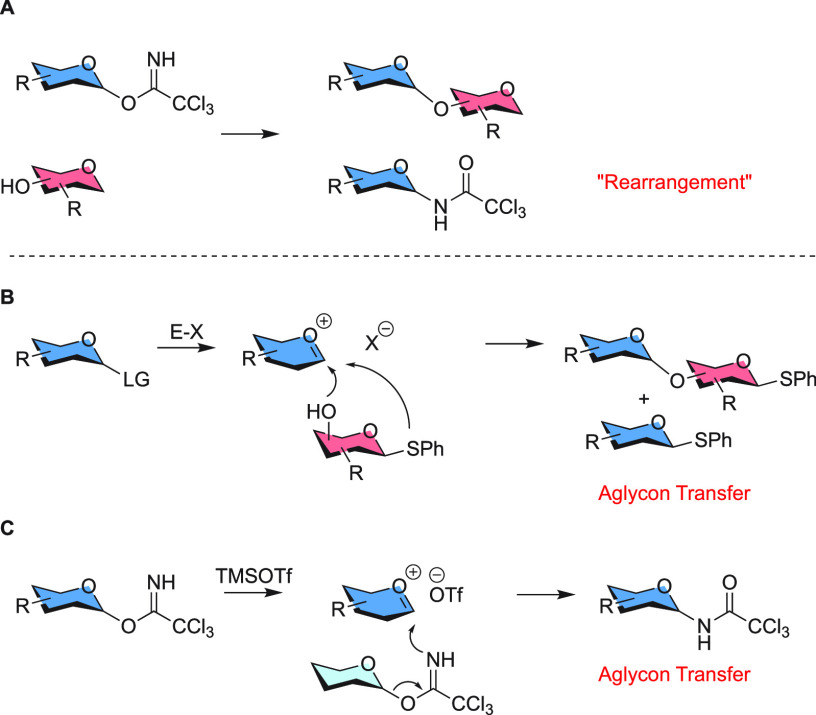
Possible Mechanisms for the “Rearrangement” of Glycosyl Imidates to Glycosyl Amides

Thioglycosides are another very popular class of glycosyl donor glycosides because these are shelf-stable and can be activated in a selective manner using a range of soft electrophiles. This has led to the widespread use of these building blocks in chemoselective one-pot glycosylation procedures, in which a thioglycoside building block having a free hydroxy group is used as an acceptor glycoside to generate a larger thioglycoside that can immediately be used for the next glycosylation through activation of thioacetal.^11^ The nucleophilicity of anomeric thiol, however, may lead to a reaction of thioacetal with an activated donor, leading to the transfer of thio-aglycon of the acceptor to the donor (Scheme 1B). This aglycon transfer process becomes important when the acceptor alcohol is relatively unreactive.^12^ Other donor types can undergo similar side reactions, and even the intermolecular transfer of a *p*-methoxy phenol group from an acceptor to an activated donor has been observed.^13^

The widespread occurrence of intermolecular aglycon transfer reactions combined with the high nucleophilicity of the trichloroacetimidate imine functionality suggests that the trichloroacetamide side products in the glycosylation reaction of trichloroacetimidate donors may originate from an intermolecular aglycon transfer type process rather than a unimolecular rearrangement,^14^ as shown in Scheme 1C. To unravel the mechanism underlying the formation of trichloroacetamide side products, here, we describe a series of crossover experiments using ^13^C/^15^N-labeled glycosyl imidate donors.

The experiment designed to differentiate between the intramolecular rearrangement and the intermolecular aglycon transfer mechanisms is depicted in Scheme 2A. Here, two different isotopically labeled derivatives of the same donor molecule were used: one containing an anomeric ^13^C label and the other a ^15^N-labeled imidate. This label could be obtained from ^15^N ammonium chloride **1** in two steps, as depicted in Scheme 2B. ^15^N-Ammonium chloride was reacted with trichloroacetyl chloride **2**, to yield trichloroacetamide **3**,^15^ which was subsequently distilled over phosphorus pentoxide to deliver ^15^N-labeled trichloroacetonitrile **4**, which was used to synthesize the ^15^N imidate donors.^16^ The ^13^C-labeled donors were synthesized from the commercially available mono-^13^C-labeled monosaccharides following well-established procedures. The “rearrangement” experiment depicted in Scheme 2 started by preparing a 1:1 mixture of anomeric ^13^C- and ^15^N-labeled imidate donors. This mixture was subjected to 10 mol % triflic acid in dichloromethane (DCM) for 30 min, during which the amide products were formed. An intramolecular mechanism would allow for the generation of amides **I** and **IV**, where the initial label is retained and exchange has not taken place. An intermolecular mechanism, on the other hand, can lead to a mixture of all four amides **I**–**IV**. Of the possible amides, compounds **II** and **III** can only be formed by intermolecular aglycon transfer. Of these amides, compound **II** can be used to report on the intermolecular aglycon transfer because it contains both the ^13^C and ^15^N label, and both labels are nuclear magnetic resonance (NMR)-active nuclei.^17,18^ For the experiments, we employed α- and β-configured glucosyl donors **5** and **6** as well as α-mannosyl imidate **7**, providing amide products **8** and **9**, respectively.

**Scheme 2:**
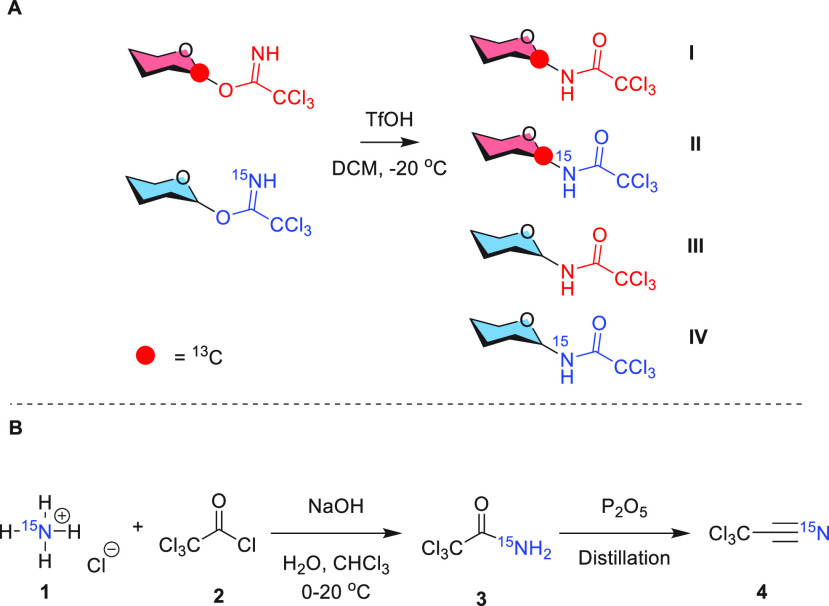
(A) Starting Materials and Products of the ^13^C/^15^N Exchange Experiments and (B) Synthesis of ^15^N-Labeled Trichloroacetonitrile

As shown in Scheme 3, donors **5** and **6** yielded products **8α**/**8β**, with an identical anomeric ratio of 5:1. Product **8α** obtained from donor **5** was isolated as a pure product and used for analysis.^19,20^ Part of the ^1^H NMR spectrum of product **8α** is depicted in Figure 1A, zooming in on the NH and H-1 regions of the spectrum. For both peaks, a characteristic pattern can be seen, where a major middle peak is flanked by two smaller peaks. For both resonances, the middle peaks correspond to the protons that are not directly attached to either ^15^N or ^13^C, while the minor peaks are the protons directly coupled to ^15^N or ^13^C, having characteristic coupling constants of 92.3 Hz for ^1^H–^15^N and 164.8 Hz for ^1^H–^13^C. The integral of the middle peaks equals the total integral of the two flanking peaks, indicating that 50% ^13^C and 50% ^15^N labels are incorporated in the product.

**Scheme 3:**
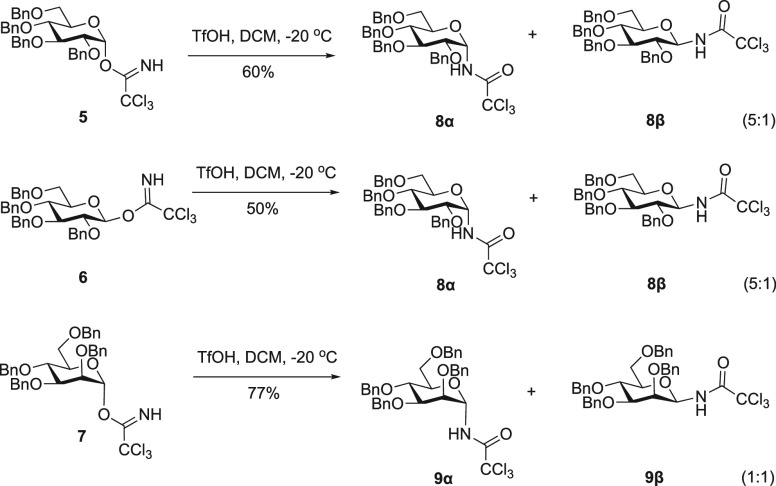
Glycosyl Imidate Donors (**5**–**7**) and Amide Products (**8** and **9**)

**Figure 1:**
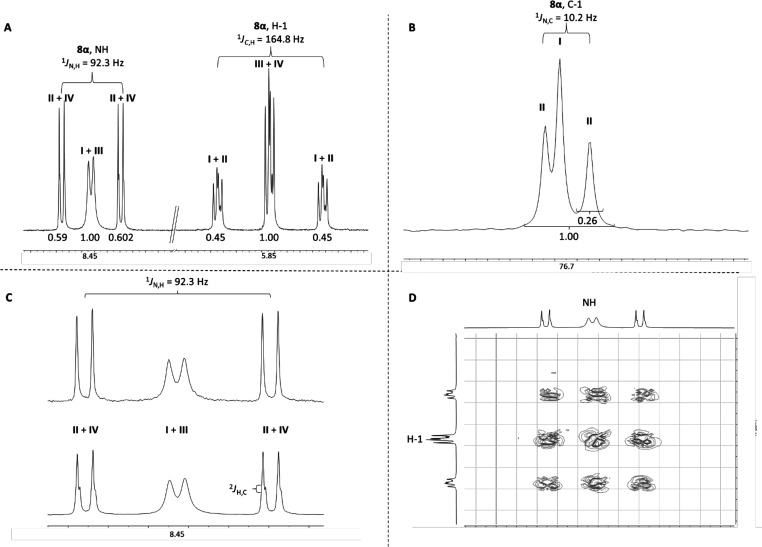
(A) ^1^H NMR of product **8α**, zoomed in onto the NH and H-1 regions. (B) ^13^C NMR of product **8α**, zoomed in onto C-1. (C) (Top) ^13^C-decoupled ^1^H NMR of product **8α**, zoomed in onto the NH region and (bottom) “normal” ^1^H NMR. (D) COSY of product **8α**, zoomed in onto the region between H-1 and NH.

In Figure 1B, the C-1 region of the ^13^C NMR spectrum of product **8α** is depicted. Here, two different peaks for C-1 can be observed: a singlet at 77.15 ppm and a doublet at 77.14 ppm. For the doublet, a coupling of 10.2 Hz is observed, typical of a one-bond ^13^C–^15^N coupling.^18^ This same coupling constant can be observed in the ^15^N NMR spectrum of product **8α** (Supporting Information). The small difference in chemical shift between the singlet and doublet observed in the ^13^C spectrum originates from a ^15^N isotope effect.^21^ The resonances observed correspond to amide **I** (the singlet at 77.15 ppm) and amide **II** (the doublet at 77.14 ppm). Integration of the peaks shows that the singlet and doublet are present in approximately equimolar amount. This product ratio can only be obtained when an intermolecular reaction has taken place, where complete scrambling of the labels takes place.

Additional evidence for the formation of distinctive amide **II** is provided by the spectra depicted in panels C and D of Figure 1. In Figure 1A, a small coupling of 2 Hz can be observed for the peaks split by ^1^H–^15^N coupling, which originates from the ^2^*J*_CH_ coupling of the anomeric ^13^C atom to the anomeric proton. In the ^13^C-decoupled spectrum (Figure 1C), this coupling is absent, turning the ddd splitting into a dd peak. Because ^2^*J*_CH_ is visible for the peaks that also couple to the ^15^N atom, this indicates that both labels are present in the same molecule. Furthermore, in the ^1^H–^1^H correlated spectroscopy (COSY) spectrum of product **8α** (Figure 1D), all of the resonances of the NH proton and H-1 correlate. Thus, the resonance of H-1 that experiences a coupling to ^13^C correlates with NH that is split by a ^1^H–^15^N coupling. Overall, these NMR experiments provide solid proof that the ^13^C and ^15^N labels are present in the same product and, thus, that the glucosyl amides form through an intermolecular aglycon transfer mechanism (Scheme 1C), rejecting the intramolecular imidate rearrangement (Scheme 1A).

For the mannosyl imidate, similar results were obtained. The α-mannosyl imidate **7** provided both the α and β amides in a 1:1 ratio, and in both products, ^13^C and ^15^N were incorporated, leading to relative integrals identical to those observed for the α-glucosyl amides.

While the experiments described above have excluded an intramolecular rearrangement mechanism, scrambling of the ^13^C and ^15^N labels may also occur in a bimolecular reaction, in which trichloroacetamide (TCA) that is formed upon activation attacks an activated donor species. While trichloroacetimidate should be significantly more nucleophilic than amide, this process cannot be ruled out on the basis of the experiments described above.^7^ Therefore, the experiment shown in Scheme 4 was designed: ^15^N-labeled glucosyl imidate **5** was activated in the presence of an equimolar amount or excess of unlabeled TCA **3**. If attack by TCA is a favorable pathway, a significant decrease of the ^15^N label in the product is to be observed.

**Scheme 4:**
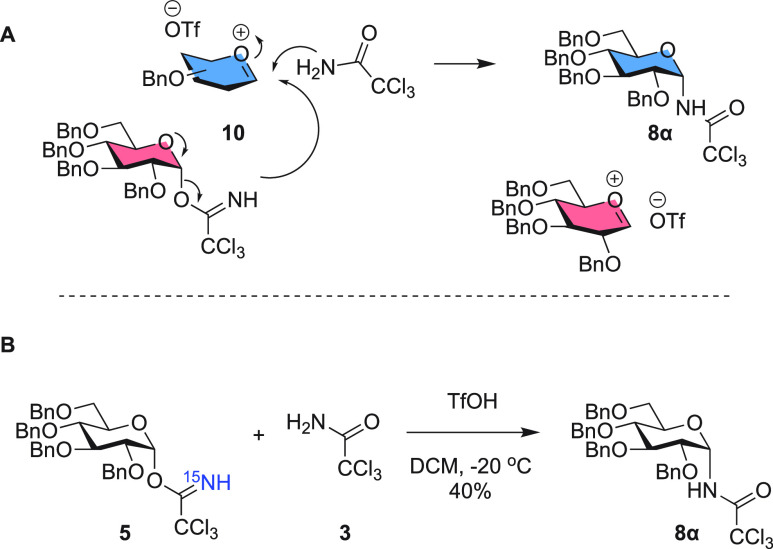
(A) Aglycon Transfer from Another Trichloroacetimidate Donor or from Generated Trichloroacetamide and (B) Experiment Designed To Differentiate between These Mechanisms

The NH region of the NMR spectrum of product **8α**, formed in these experiments, is shown in Figure 2, with the left panel showing the spectrum for the experiment with 1 equiv of TCA and the right panel for the spectrum for the experiment with 3 equiv of TCA. The relative integrals for the double doublet of ^15^N-H versus the integral of the doublet of the ^14^N-H peak, 0.72 versus 0.28 (1 equiv of TCA) and 0.70 versus 0.30 (3 equiv of TCA), indicate that the main product originates from aglycon transfer of imidate, even when an excess of TCA is used as a competitor.

**Figure 2:**
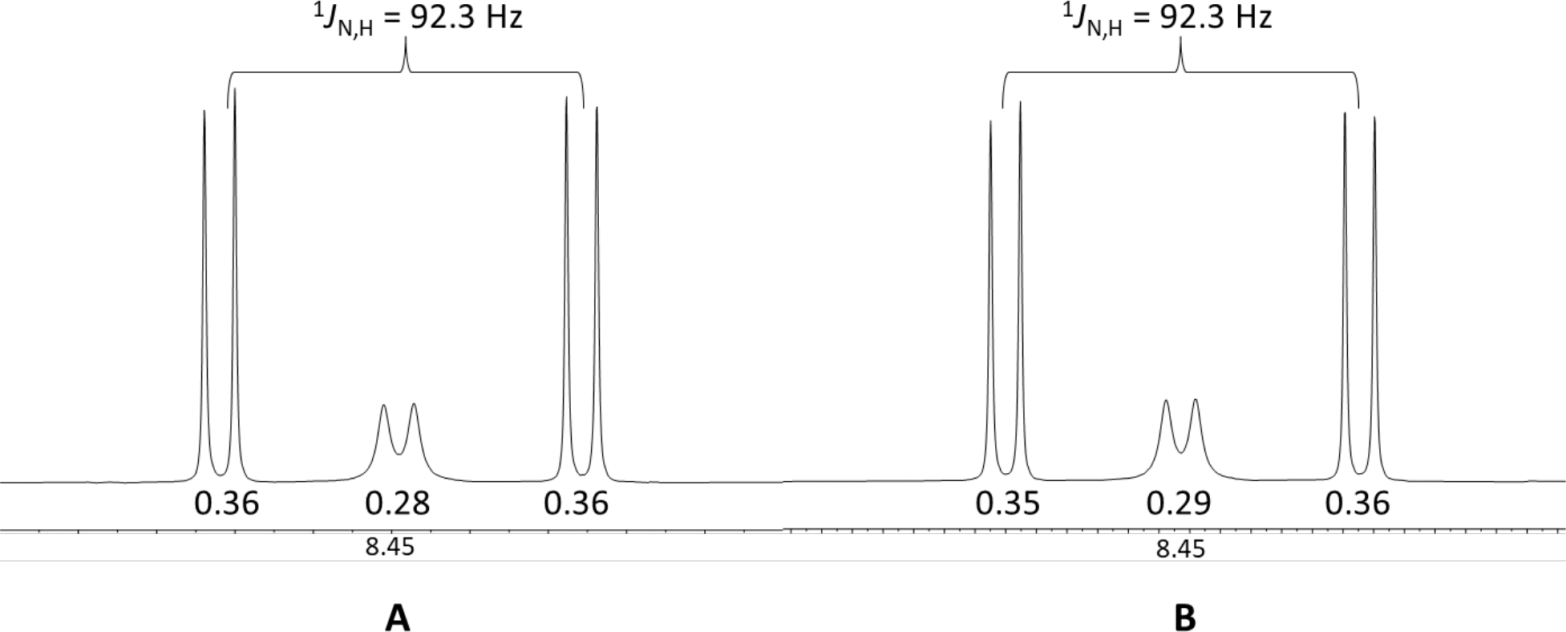
^1^H NMR of amide **8α**, zoomed in onto the NH range, formed after the experiment depicted in Scheme 4 with (A) 1 equiv or (B) 3 equiv of additional trichloroacetamide.

Finally, the potential activation of ^14^N-trichloroacetamide **8α** under acidic reaction conditions was examined, because expulsion of trichloroacetamide from product **8α** and addition of ^15^N-TCA to the activated donor could lead to scrambling of anomeric ^14^N/^15^N trichloroacetamides. Thus, ^14^N-trichloroacetamide **8α** was subjected to acidic imidate transfer conditions in the presence of ^15^N TCA **3**. No uptake of ^15^N could be observed in the product of this reaction, indicating that anomeric trichloroacetamide is stable under the reaction conditions and the ^14^N/^15^N incorporation in product **8α** is the result of a glycosylation reaction under kinetic control.

In conclusion, to differentiate between intramolecular rearrangement and intermolecular aglycon transfer reaction mechanisms to account for the formation of anomeric trichloroacetamides from trichloroacetimidate donors, we have used ^13^C and ^15^N isotopic labeling experiments. These have unambiguously shown that, in the reaction studied, the major route of anomeric trichloroacetamide formation follows the intermolecular aglycon transfer path, in which glycosyl trichloroacetimidate attacks an activated donor species to produce another copy of an activated donor alongside anomeric trichloroacetamide. This mechanism well explains the success of the “inverse” procedure, in which the amount of a reactive glycosyl donor in a glycosylation reaction with a poor nucleophile is kept to a minimum. It also provides an explanantion for the success of the related *N*-phenyl trifluoroacetimidate donors,^22^ because these donors are less nucleophilic and, therefore, less prone to the formation of the acetamide side products. To the best of our knowledge, only one example of the formation of *N*-phenyl trifluoroacetamide has been reported in the literature to date.^23^ The detailed mechanistic insight described here will aid our further understanding of the glycosylation reaction and support the rational optimization of reaction conditions^24^ to enable the assembly of ever more complex oligosaccharides and glycoconjugates.

## Data Availability

The data underlying this study are available in the published article and its Supporting Information.

## Supporting Information Available

Experimental procedures and NMR characterization for all new compounds (PDF)
