# Supplementary material for: Formation of Glycosyl Trichloroacetamides from Trichloroacetimidate Donors Occurs through an Intermolecular Aglycon Transfer Reaction
Source: Org Lett. 2023 Aug 14;25(33):6128–32. doi: 10.1021/acs.orglett.3c02196 (PMC10463224; doi:10.1021/acs.orglett.3c02196)

Supporting Information

Formation of glycosyl trichloroacetamides from trichloroacetimidate donors occurs through an intermolecular aglycon transfer reaction

Koen N. A. van de Vrande, Dmitri V. Filippov and Jeroen D. C. Codée*

Leiden Institute of Chemistry, Leiden University, Einsteinweg 55, 2333 CC Leiden, The Netherlands

*Email: jcodee@chem.leidenuniv.nl

Contents

1. General Experimental Procedures S3
1.1 General Procedure for TfOH catalysed Imidate Rearrangement S3
2. Reagent Synthesis S4
15N Trichloroacetamide (3) S4
^{15}N Trichloro acetonitrile (4) S4
Phenyl 2,3,4,6-tetra-O-benzyl-1-thio-β-D-glucospyranoside (S1) S4
2,3,4,6-tetra-O-benzyl-D-glucospyranoside (S2) S5
2,3,4,6-tetra-O-benzyl-α-D-glucopyranosyl trichloroacetimidate (5) S5
2,3,4,6-tetra-O-benzyl-β-D-glucopyranosyl trichloroacetimidate (6) S6
Phenyl 2,3,4,6-tetra-O-benzyl-1-thio-α-D-mannopyranoside (S3) S7
2,3,4,6-tetra-O-benzyl-D-mannospyranoside (S4) S7
2,3,4,6-tetra-O-benzyl-α-D-mannopyranosyl trichloroacetimidate (7) S7
3. ^{13}C / ^{15}N Exchange Experiment S9
N-trichloroacetyl-2,3,4,6-tetra-O-benzyl-α-D-glucopyranosylamide (8) S9
N-trichloroacetyl-2,3,4,6-tetra-O-benzyl-α/β-D-mannopyranosylamide (9) S9
4. Mode of Attack Determination S10
N-trichloroacetyl-2,3,4,6-tetra-O-benzyl-α-D-glucopyranosylamide (8) S10
5. Reversibility Experiment S11
N-trichloroacetyl-2,3,4,6-tetra-O-benzyl-α-D-glucopyranosylamide (8) S11
6. References S12
7. Spectral Data S13

1. General Experimental Procedures

All chemicals were of commercial grade and used as received unless stated otherwise. Dichloromethane (DCM) and Dimethylformamide (DMF) were stored over activated 4 Å molecular sieves at least 18h before use. Flash column chromatography was performed on silica gel 60 Å (0.04 – 0.063 mm, Screening Devices B.V.). Thin layer chromatography (TLC) was conducted on TLC silica gel 60 plates (Kieselgel 60 F254, Merck) with UV detection (254 nm) and by spraying with a solution of $(NH_4)_6Mo_2O_{24}\cdot H_2O$ (25 g/L) and $(NH_4)_4Ce(SO_4)_2\cdot 2H_2O$ (10 g/L) in 10% aqueous sulphuric acid followed by charring at 250 °C. High-resolution mass spectrometry (HRMS) was performed on a Thermo Finnigan LTQ Orbitrap mass spectrometer equipped with an electrospray ion source in positive-ion mode (source voltage 3.5 kV, sheath gas flow 10, capillary temperature 275 °C) with resolution R = 60.000 at *m/z* 400 (mass range of 150-4000) and dioctylphalate (*m/z* = 391.28428) as lock mass. ^{1}H, ^{13}C and ^{15}N NMR spectra were recorded on Brucker AV-400, Brucker DMX-400 and Brucker AV-500 NMR instruments. Chemical shifts (δ) are given in part per million (ppm) relative to tetramethylsilane as an internal standard or the residual signal of the deuterated solvent. Coupling constants (*J*) are given in Hertz (Hz). All presented 13C spectra are proton decoupled. Structural assignments were made with additional information from gCOSY, gHSQC, and gHMBC experiments. IR spectra were recorded on a Shimadzu FTIR-8300 IR spectrometer and are reported in cm-1.

1.1 General Procedure for TfOH catalysed Imidate Rearrangement

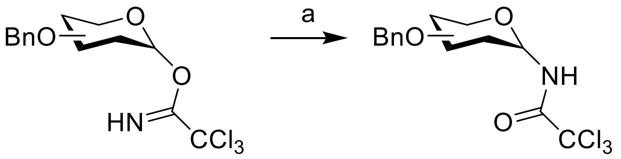

Scheme 1: General procedure for the imidate to amide exchange. Reagents and conditions: a) TfOH (10 mol%), DCM, -20 °C.

A mixture containing the anomeric ^{13}C glycosyl donor (50 mg, 0.08 mmol, 0.5 eq) and the ^{15}N donor (50 mg, 0.08 mmol, 0.5 eq) was prepared and the ^{1}H, ^{13}C and ^{15}N NMR were measured. The sample was concentrated *in vacuo* and co-evaporated twice with dry toluene. The oil was dissolved in dry DCM (3 mL) and dried molsieves (3Å, rods) were added. The reaction mixture was cooled to -20 °C and TfOH (1.3 µL, 0.02 mmol, 0.1 eq) was added. After stirring at -20 °C for 30 min, the reaction was quenched with solid $NaHCO_3$ and allowed to warm to room temperature. The suspension was filtered over cotton and washed with saturated aqueous $NaHCO_3$ twice, once with water, dried over Na_2SO_4 and concentrated *in vacuo*. The crude ^{1}H, ^{13}C and ^{15}N NMR were measured before purification by flash column chromatography yielded the pure amide.

2. Reagent Synthesis

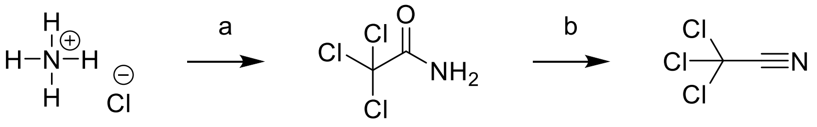

Scheme 2: Synthesis of ^{15}N labelled trichloroacetonitrile. Reagents and conditions: a) Trichloroacetyl chloride, NaOH, Chloroform, H_2O, 0-25 °C; b) P_2O_5, distillation.

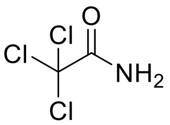

^{15}N Trichloroacetamide (3)

To a solution of trichloroacetyl chloride (10.3 mL, 93 mmol, 1.01 eq) in chloroform (250 mL) at 0 °C were added ^{15}N ammonium chloride (5.0 g, 92 mmol, 1.0 eq) and a solution of NaOH (7.63 g, 189 mmol, 2.06 eq) in water (70 mL). The reaction mixture was stirred at 0 °C for 15 min and then allowed to warm to r.t. in 1 h. The reaction was filtered over a glass filter and the residue was washed with water and chloroform. The two layers of the filtrate were separated and the aqueous layer was extracted twice with Et_2O. The combined organic phase was dried over Na_2SO_4 and concentrated *in vacuo*. The crude was purified by flash column chromatography (10-20% EtOAc in pentane) and combined with the solid residue to give the title compound as a white solid (7.01 g, 42.9 mmol, 47%). IR (thin film): 608, 744, 823, 1099, 1346, 1375, 1608, 1685, 3173, 3235, 3308, 3354. ^1H NMR (400 MHz, Acetone) δ 8.07 – 7.28 (br. s, 1H). ^{13}C NMR (101 MHz, Acetone) δ 163.8 (d, $^1J_{C,N}$ = 21.0 Hz, *C*=ONH_2), 93.7 (d, $^2J_{C,N}$ = 13.1 Hz, *C*Cl_3). ^{15}N NMR (41 MHz, Acetone) δ 92.6 HRMS (M+2ACN+H^+) 244.97761, found 244.97809.

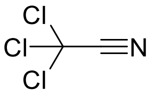

^{15}N Trichloro acetonitrile (4)

A mixture of ^{15}N trichloroacetamide (1.93 g, 11.8 mmol, 1.0 eq) and phosphorus pentoxide (3.5 g, 12.3 mmol, 1.04 eq) was mixed thoroughly. The mixture was heated with a heat gun and the formed liquid was collected by distillation using a short-path distillation setup. IR (thin film): 643, 744, 821, 1100, 1346, 1375, 1682, 3235. ^{13}C NMR (126 MHz, CDCl3) δ 113.2 (d, $^1J_{C,N}$ = 14.4 Hz, *C*N), 70.2 (d, $^2J_{C,N}$ = 4.0 Hz, *C*Cl_3) ^{15}N NMR (51 MHz, CDCl3) δ 255.8.

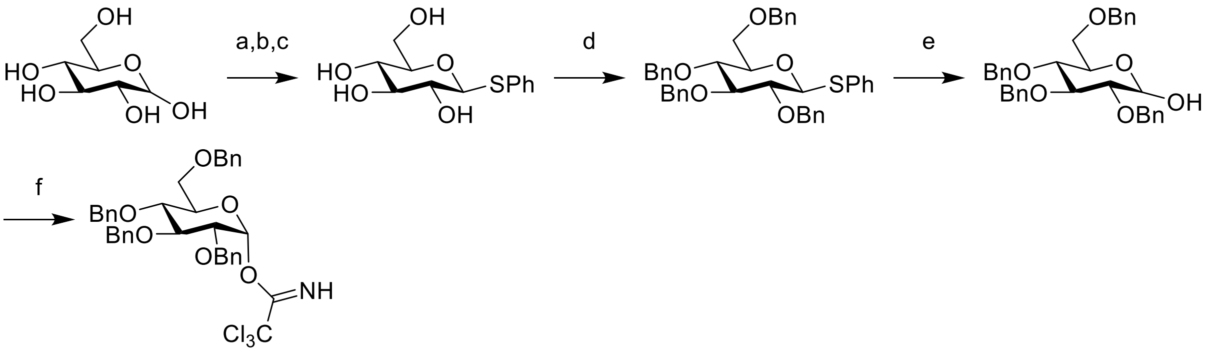

Scheme 3: Synthesis of the α-glucose imidate donor. Reagents and conditions: a) Ac_2O, $HClO_4$, r.t., overnight; b) PhSH, BF_3●Et_2O, DCM, reflux, overnight; c) NaOMe, MeOH, r.t., overnight; d) BnBr, NaH, DMF, 0-25 °C,; e) NBS, Acetone, H_2O, 70 min., 0-25 °C; f) Trichloroacetonitrile, NaH, DCM, 2:40 h, 0-25 °C.

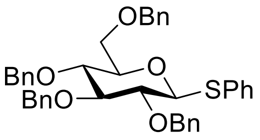

Phenyl 2,3,4,6-tetra-O-benzyl-1-thio-β-D-glucospyranoside (S1)

To a solution of phenyl 1-thio-β-D-glucopyranoside (1.31 g, 4.97 mmol, 1.0 eq) (prepared according to literature[1]) in dry DMF (24 mL) at 0 °C were added NaH (60 w%, 0.93 g, 23 mmol, 4.8 eq) and BnBr (2.7 mL, 23 mmol, 4.8 eq). The reaction mixture was stirred at r.t. overnight. After completion, the reaction was cooled to 0 °C and quenched with MeOH. The crude was diluted with water and extracted trice with Et_2O. The combined organic phases were dried over Na_2SO_4 and concentrated *in vacuo*. Flash column chromatography of

the crude (5-20% EtOAc in pentane) gave the title compound as a white solid (3.08 g, 4.97 mmol, *quant.*). IR (thin film): 644, 744, 821, 1100, 1346, 1375, 1611, 1682, 1694, 3235, ^{1}H NMR (400 MHz, CDCl3) δ 7.70 – 7.54 (m, 2H, CH_{arom}), 7.46 – 7.13 (m, 23H, CH_{arom}), 4.92 – 4.79 (m, 4H, CH_2 Bn), 4.73 (d, J = 10.2 Hz, 1H, CH_2 Bn), 4.67 (d, J = 9.8 Hz, 1H, H-1), 4.64 – 4.57 (m, 2H, CH_2 Bn), 4.54 (d, J = 12.0 Hz, 1H, CH_2 Bn), 3.84 – 3.59 (m, 4H, H-3, H-4, H-6), 3.58 – 3.46 (m, 2H, H-2, H-5) ^{13}C NMR (101 MHz, CDCl3) δ 138.5, 138.4, 138.1, 133.9 ($C_{q,\ arom}$), 132.1, 129.0, 128.6, 128.6, 128.5, 128.4, 128.1, 128.0, 128.0, 127.9, 127.8, 127.7, 127.6 (CH_{arom}), 87.6 (C-1), 86.9 (C-3, C-4), 80.9 (C-2), 79.2 (C-5), 77.9 (C-3, C-4), 76.0, 75.6, 75.2, 73.5 (*C*H_2 Bn), 69.1 (C-6). HRMS ($M+NH_4^+$): cald. for $C_{39}{}^{13}CH_{40}O_5S_1NH_4$ 651.29683, found 651.29759. Spectra in agreement with literature.[2]

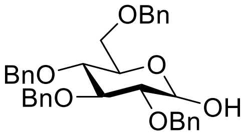

2,3,4,6-tetra-O-benzyl-D-glucospyranoside (S2)

To a solution of phenyl 2,3,4,6-tetra-O-benzyl-1-thio-β-D-glucospyranoside (0.57 g, 0.9 mmol, 1.0 eq) in acetone (8 mL) and water (0.9 mL) at 0 °C was added NBS (0.48 g, 2.7 mmol, 3.0 eq).The reaction was stirred at r.t. for 70 min, after which it was quenched with saturated aqueous $Na_2S_2O_3$ and extracted trice with EtOAc. The combined organic phase was washed with saturated aqueous $NaHCO_3$ and brine, dried over Na_2SO_4 and concentrated *in vacuo*. Flash column chromatography (20-30% EtOAc in pentane) yielded the title compound as a white solid (0.35 g, 0.65 mmol, 71%). Data reported for a 2.5 : 1 α/β mixture: %). IR (thin film): 692, 731, 1053, 1356, 1452, 1584, 3027. ^{1}H NMR (400 MHz, CDCl3) δ 7.42 – 7.22 (m, 23H, CH_{arom}), 7.21 – 7.10 (m, 3H, CH_{arom}), 5.22 (dd, J = 3.1, 3.1 Hz, 1H, H-1α), 4.94 (dd, J = 10.8, 7.6 Hz, 2H, CH_2 Bn), 4.89 – 4.66 (m, 6H, CH_2 Bn, H-1β), 4.63 – 4.54 (m, 2H, CH_2 Bn), 4.48 (dd, J = 11.5, 3.2 Hz, 3H, CH_2 Bn), 4.03 (ddd, J = 10.1, 4.0, 2.1 Hz, 1H, H-5α), 3.97 (t, J = 9.3 Hz, 1H, H-4α), 3.74 – 3.51 (m, 6H, H-2α, H-3α, Hβ), 3.45 (d, J = 5.4 Hz, 0.4H, Hβ), 3.40 (dd, J = 9.1, 7.7 Hz, 0.4H, Hβ), 3.03 (d, J = 2.6 Hz, 1H, OH). ^{13}C NMR (101 MHz, CDCl3) δ 138.8, 138.3, 138.0 ($C_{q,\ arom}$), 128.7, 128.6, 128.5, 128.3, 128.2, 128.1, 128.1, 128.1, 128.0, 128.0, 127.9, 127.9, 127.8 (CH_{arom}), 97.6 (C-1β), 91.5 (C-1α), 84.7, 83.2 (Cβ), 81.9 (C-5α), 80.1 (C-2, C-3α), 77.91, 77.8 (C-2, C-3α), 75.9, 75.2, 73.7, 73.6, 73.4 (CH_2 Bn), 70.5 (C-4α), 69.0 (C-6β), 68.7 (C-6α). HRMS ($M+NH_4^+$): cald. for $C_{33}{}^{13}C_1H_{36}O_6SNH_4$ 559.28837, found 559.28826. Spectra in agreement with literature.[3]

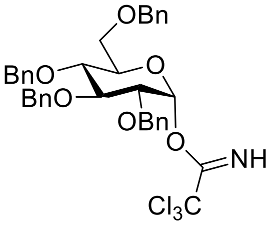

2,3,4,6-tetra-O-benzyl-α-D-glucopyranosyl trichloroacetimidate (5)

To a solution of 2,3,4,6-tetra-O-benzyl-D-glucospyranoside (150 mg, 0.28 mmol, 1.0 eq) in dry DCM (1.7 mL) were added trichloroacetonitrile (0.13 mL, 1.3 mmol, 4.6 eq) and NaH (60 w%, 1 mg, 0.03 mmol, 0.1 eq). The reaction mixture was stirred at r.t. for 10 min, after which it was cooled to 0 °C and additional NaH (60 w%, 14 mg, 0.36 mmol, 1.3 eq) was added. The reaction was allowed to warm to r.t. and stirred for an additional 2.5 h. The reaction mixture was filtered and concentrated *in vacuo*. Flash column chromatography (5-20% EtOAc in pentane) yielded the title compound as a clear oil (120-160 mg, 65-85%). Data reported for isotopically unlabelled compound: ^{1}H NMR (400 MHz, CDCl3) δ 8.58 (s, 1H, NH), 7.39 – 7.24 (m, 18H, CH_{arom}), 7.18 – 7.11 (m, 2H, CH_{arom}), 6.52 (d, J = 3.4 Hz, 1H, H-1), 4.96 (d, J = 11.0 Hz, 1H, CH_2 Bn), 4.84 (t, J = 10.4 Hz, 2H, CH_2 Bn), 4.78 – 4.65 (m, 2H, CH_2 Bn), 4.60 (d, J = 12.0 Hz, 1H, CH_2 Bn), 4.52 (d, J = 10.7 Hz, 1H, CH_2 Bn), 4.46 (d, J = 12.1 Hz, 1H, CH_2 Bn), 4.05 (t, J = 9.4 Hz, 1H, H-3), 3.99 (ddd, J = 10.2, 3.2, 1.9 Hz, 1H, H-5), 3.84 – 3.74 (m, 3H, H-2, H-4, H-6), 3.66 (dd, J = 11.0, 2.0 Hz, 1H, H-6). ^{13}C NMR (101 MHz, CDCl3) δ 161.4 (C=N), 138.7, 138.1, 138.1, 137.9 ($C_{q,\ arom}$), 128.8, 128.8, 128.6, 128.5, 128.5, 128.5, 128.5, 128.2, 128.1, 128.1, 128.0, 128.0, 127.9, 127.8, 127.7, 127.7 (CH_{arom}), 94.5 (C-1), 81.5 (C-3), 79.4 (C-2, C-4), 76.9 (C-2, C-4), 75.8, 75.5, 73.6 (CH_2 Bn), 73.2 (C-5), 73.0 (CH_2 Bn), 68.1 (C-6), 30.4 (CCl_3). Characteristic peaks for the ^{13}C labelled compound: IR (thin film): 694, 736, 795, 1061. ^{1}H NMR (400 MHz, CDCl3) δ 6.52 (dd, J = 175.2,

3.4 Hz, 1H, H-1). Characteristic peaks for the ^{15}N labelled compound: IR (thin film): 695, 745, 800, 1024, 1064, 1086. ^{1}H NMR (400 MHz, CDCl3) δ 8.57 (d, J = 62.1 Hz, 1H, NH). ^{13}C NMR (101 MHz, CDCl3) δ 161.3 (d, J = 9.9 Hz, C=N), 94.4 (d, J = 3.5 Hz, C-1). ^{15}N NMR (41 MHz, CDCl3) δ 212.2. HRMS (M+Na$^+$): cald. for $C_{33}{}^{13}C_1H_{36}Cl_3NO_6SNa$ 707.15340, found 707.15393 cald. for $C_{36}H_{36}Cl_3{}^{15}NO_6Na$ 707.14708, found 707.14715 Spectra in agreement with literature.[4]

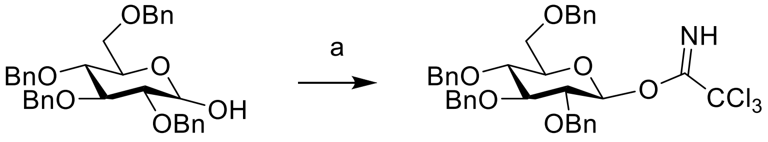

Scheme 4: Synthesis of β-glucose imidate donor. Reagents and conditions: a) Trichloroacetonitrile, K_2CO_3, DCM, r.t., overnight.

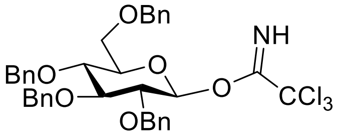

2,3,4,6-tetra-O-benzyl-β-D-glucopyranosyl trichloroacetimidate (6)

To a solution of 2,3,4,6-tetra-O-benzyl-D-glucospyranoside (0.53 g, 0.92 mmol, 1.0 eq) in dry DCM (7.5 mL) were added trichloroacetonitrile (0.8 mL, 7.8 mmol, 8.4 eq) and K_2CO_3 (0.55 g, 4.0 mmol, 4.3 eq). The reaction mixture was stirred overnight at r.t. After completion, the reaction mixture was filtered and concentrated *in vacuo*. Flash column chromatography (10-20% EtOAc in pentane + 1% Et_3N) yielded the title compound as a clear oil (440.9 mg, 0.64 mmol, 70%). Data reported for isotopically unlabelled compound: ^{1}H NMR (500 MHz, $CDCl_3$) δ 8.70 (s, 1H, NH), 7.38 – 7.24 (m, 18H, CH_{arom}), 7.17 (m, 2H, CH_{arom}), 6.00 – 5.71 (m, 1H, H-1), 4.94 (d, *J* = 10.8 Hz, 1H, CH_2 Bn), 4.91 (d, *J* = 11.0 Hz, 1H, CH_2 Bn), 4.82 (d, *J* = 10.8 Hz, 2H, CH_2 Bn), 4.76 (d, *J* = 10.8 Hz, 1H, CH_2 Bn), 4.62 (d, *J* = 12.2 Hz, 1H, CH_2 Bn), 4.58 (d, *J* = 10.8 Hz, 1H, CH_2 Bn), 4.54 (d, *J* = 12.2 Hz, 1H, CH_2 Bn), 3.75 (m, *J* = 2.7 Hz, 6H, H-2, H-3, H-4, H-5, H-6), 3.63 (m, 1H, H-3, H-4, H-5). ^{13}C NMR (126 MHz, $CDCl_3$) δ 161.3 (C=N), 138.6, 138.3, 138.1, 138.1 ($C_{q, arom}$), 128.5, 128.5, 128.5, 128.1, 128.1, 128.0, 127.9, 127.9, 127.8, 127.8 (CH_{arom}), 98.5 (C-1), 84.7, 81.1, 77.4, 76.0 (C-2, C-3, C-4, C-5), 75.8, 75.1, 75.1, 73.5 (CH_2 Bn), 68.3 (C-6). Characteristic peaks for the ^{13}C labelled compound: IR (thin film): 693, 731, 1044, 1073, 1286, 1362, 1452, 1496, 1674. ^{1}H NMR (400 MHz, $CDCl_3$) δ 6.10 – 5.53 (m, 1H, H-1). Characteristic peaks for the ^{15}N labelled compound: %). IR (thin film):693, 731, 1025, 1056, 1209, 1287, 1452, 1496, 1655, 2863. ^{1}H NMR (400 MHz, $CDCl_3$) δ 8.70 (d, *J* = 61.5 Hz, 1H, NH). ^{13}C NMR (101 MHz, $CDCl_3$) δ 161.3 (d, *J* = 9.9 Hz, C=N), 98.5 (d, *J* = 3.2 Hz, C-1). ^{15}N NMR (41 MHz, $CDCl_3$) δ 215.3. HRMS (M+Na$^+$): cald. for $C_{33}{}^{13}C_1H_{36}Cl_3NO_6SNa$ 707.15340, found 707.15339 cald. for $C_{36}H_{36}Cl_3{}^{15}NO_6Na$ 707.14708, found 707.14752. Spectra in agreement with literature.[4]

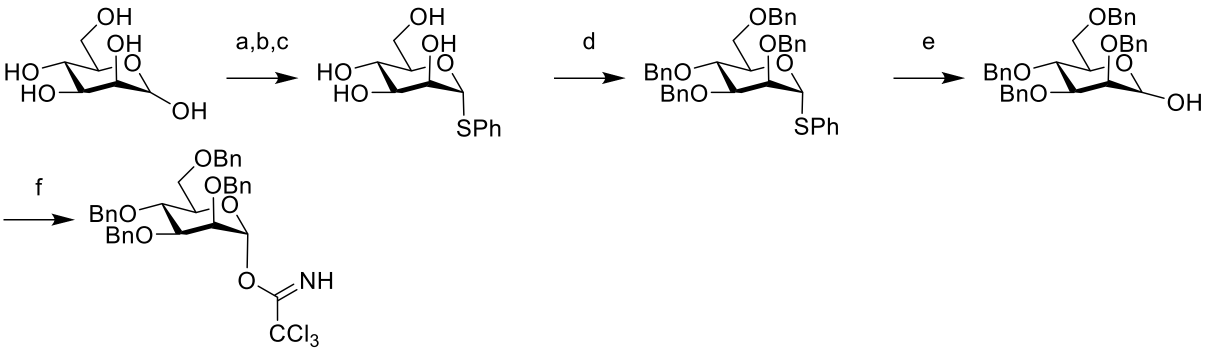

Scheme 5: Synthesis of the α-mannose imidate donor. Reagents and conditions: a) Ac_2O, $HClO_4$, r.t., overnight; b) PhSH, BF_3●Et_2O, DCM, r.t., overnight; c) NaOMe, MeOH, r.t., overnight; d) BnBr, NaH, DMF, 0-25 ^oC,; e) NBS, Acetone, H_2O, 70 min., 0-25 ^oC; f) Trichloroacetonitrile, NaH, DCM, 2:40 h, 0-25 ^oC.

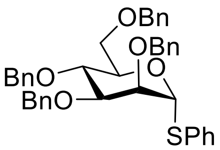

Phenyl 2,3,4,6-tetra-O-benzyl-1-thio-α-D-mannopyranoside (S3)

To a solution of phenyl 1-thio-α-D-mannopyranoside (1.98 g, 7.3 mmol, 1.0 eq) (prepared according to literature[5]) in dry DMF (34 mL) at 0 °C were added NaH (60 w%, 1.40 g, 35 mmol, 4.8 eq) and BnBr (42 mL, 35 mmol, 4.8 eq). The reaction mixture was stirred at room temperature overnight and afterwards quenched with MeOH and diluted with Et_2O. The mixture was washed trice with water and once with brine, dried over Na_2SO_4 and concentrated *in vacuo*. Flash column chromatography (5-20% Et_2O in pentane) yielded the title compound as a white oil (4.26 g, 6.7 mmol, 92%). IR (thin film): 694, 728, 1015, 1054, 1075. ^1H NMR (400 MHz, $CDCl_3$) δ 7.51 – 7.40 (m, 2H, CH_{arom}), 7.39 – 7.16 (m, 23H, CH_{arom}), 5.62 (d, *J* = 1.8 Hz, 1H, H-1), 4.91 (d, *J* = 10.8 Hz, 1H, CH_2 Bn), 4.73 (d, *J* = 12.2 Hz, 1H, CH_2 Bn), 4.65 (d, *J* = 9.6 Hz, 1H, CH_2 Bn), 4.62 (d, *J* = 10.1 Hz, 1H, CH_2 Bn), 4.60 (d, *J* = 2.1 Hz, 2H, CH_2 Bn), 4.53 (d, *J* = 10.7 Hz, 1H, CH_2 Bn), 4.48 (d, *J* = 11.9 Hz, 1H, CH_2 Bn), 4.29 (ddd, *J* = 9.9, 5.0, 2.0 Hz, 1H, H-4), 4.07 (td, *J* = 9.5, 1.8 Hz, 1H, H-3), 4.00 (dd, *J* = 3.1, 1.7 Hz, 1H, H-2), 3.91 – 3.80 (m, 2H, H-5, H-6), 3.75 (dt, *J* = 10.9, 1.8 Hz, 1H, H-6). ^{13}C NMR (101 MHz, $CDCl_3$) δ 138.6, 138.5, 138.3, 138.0, 134.5 ($C_{q, arom}$), 131.7, 129.1, 128.5, 128.5, 128.5, 128.4, 128.1, 128.0, 128.0, 127.9, 127.8, 127.8, 127.6, 127.5 (CH_{arom}), 85.8 (C-1), 80.3 (C-5), 76.3 (C-2), 75.3 (CH_2 Bn), 75.1 (C-3), 73.4 (CH_2 Bn), 72.9 (C-4), 72.2 (CH_2 Bn), 72.0 (CH_2 Bn), 69.3 (C-6). HRMS ($M+NH_4^+$): cald. for $C_{39}{}^{13}CH_{40}O_5SNH_4$ 651.29683, found 651.29621. Spectra in agreement with literature.[6]

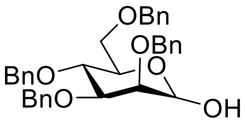

2,3,4,6-tetra-O-benzyl-D-mannospyranoside (S4)

To a solution of Phenyl 2,3,4,6-tetra-O-benzyl-1-thio-α-D-mannopyranoside (0.51 g, 0.79 mmol, 1.0 eq) in acetone (7 mL) and water (0.7 mL) at 0 °C was added NBS (0.42 g, 2.4 mmol, 3.0 eq) and the reaction mixture was stirred at room temperature for 1h. The reaction was quenched with saturated aqueous $Na_2S_2O_3$ and extracted trice with EtOAc. The combined organic phase was washed with saturated aqueous $NaHCO_3$ and brine, dried over Na_2SO_4 and concentrated *in vacuo*. Flash column chromatography (10-30% EtOAc in pentane) yielded the title compound as a clear oil (0.37 g, 0.68 mmol, 87%). IR (thin film): 692, 731, 1020, 1053. ^1H NMR (400 MHz, $CDCl_3$) δ 7.46 – 7.22 (m, 18H, CH_{arom}), 7.20 – 7.11 (m, 2H, CH_{arom}), 5.25 (dd, *J* = 3.4, 1.9 Hz, 1H, H-1), 4.88 (d, *J* = 10.9 Hz, 1H, CH_2 Bn), 4.72 (d, *J* = 5.4 Hz, 2H, CH_2 Bn), 4.61 (s, 2H, CH_2 Bn), 4.55 (d, *J* = 8.9 Hz, 2H, CH_2 Bn), 4.52 – 4.46 (m, 1H, CH_2 Bn), 4.03 (ddd, *J* = 8.9, 6.3, 2.2 Hz, 1H, H-5), 3.95 (dd, *J* = 9.4, 3.1 Hz, 1H, H-3), 3.85 (t, *J* = 9.5 Hz, 1H, H-4), 3.79 (dd, *J* = 3.0, 2.0 Hz, 1H, H-2), 3.76 – 3.63 (m, 2H, H-6), 3.27 (d, *J* = 3.4 Hz, 1H, OH). ^{13}C NMR (101 MHz, $CDCl_3$) δ 138.6, 138.5, 138.5, 138.2 ($C_{q, arom}$), 128.5, 128.5, 128.5, 128.1, 128.0, 127.8, 127.7, 127.7 (CH_{arom}), 92.9 (C-1), 79.9 (C-3), 75.3 (C-4), 75.2 (CH_2 Bn), 74.9 (C-2), 73.4 (CH_2 Bn), 72.8 (CH_2 Bn), 72.3 (CH_2 Bn), 71.7 (C-5), 69.7 (C-6). HRMS ($M+NH_4^+$): cald. for $C_{33}{}^{13}CH_{36}O_6NH_4$ 559.28837, found 559.28814. Spectra in agreement with literature.[4]

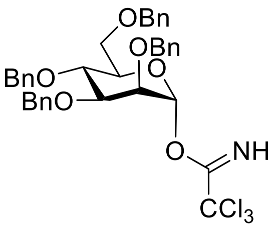

2,3,4,6-tetra-O-benzyl-α-D-mannopyranosyl trichloroacetimidate (7)

To a solution of 2,3,4,6-tetra-O-benzyl-D-mannopyranoside (0.01 g, 0.19 mmol, 1.0 eq) and trichloro acetonitrile (0.1 mL, 0.85 mmol, 4.6 eq) in dry DCM (1.1 mL) was added NaH (60 w%, 1 mg, 0.02 mmol, 0.1 eq) and the reaction was stirred for 10 min. The reaction mixture was cooled to 0 °C and additional NaH (60 w%, 10 mg. 0.24 mmol, 1.3 eq) was added. The reaction was stirred for 2.5 h, filtered and concentrated *in vacuo*. Flash column chromatography (5-10% EtOAc in pentane + 1% Et_3N) yielded the title compound as a colourless oil. Data reported for isotopically unlabelled compound: ^1H NMR (400 MHz, $CDCl_3$) δ 8.52 (s, 1H, NH), 7.49 – 7.14 (m, 20H, CH_{arom}), 6.38 (d, *J* = 2.1 Hz, 1H, H-1), 4.90 (d, *J* = 10.6 Hz, 1H, CH_2 Bn), 4.76 (t, *J* = 2.4 Hz, 2H, CH_2 Bn), 4.73 – 4.48 (m, 5H, CH_2 Bn), 4.16 (t, *J* = 9.7 Hz, 1H, H-4), 4.01 – 3.90 (m, 2H, H-3, H-5), 3.87 (t, *J* = 2.6 Hz, 1H, H-2), 3.83

(dd, J = 11.1, 4.3 Hz, 1H, H-6), 3.72 (dd, J = 11.3, 2.1 Hz, 1H, H-6). ^{13}C NMR (101 MHz, $CDCl_3$) δ 160.5 (C=N), 138.3, 138.3, 138.1, 138.0 ($C_{q, arom}$), 128.8, 128.7, 128.6, 128.6, 128.5, 128.5, 128.4, 128.4, 128.3, 128.2, 128.2, 128.1, 128.1, 128.0, 128.0, 127.9, 127.9, 127.9, 127.8, 127.8, 127.7, 127.7, 127.7, 127.6 (CH_{arom}), 96.1 (C-1), 78.9 (C-3, C-4), 75.4 (CH_2 Bn), 74.9 (C-4), 74.2 (C-3, C-4), 73.6 (C-2), 73.3 (CH_2 Bn), 72.7 (CH_2 Bn), 72.4 (CH_2 Bn), 68.9 (C-6). Characteristic peaks for the anomeric ^{13}C labelled compound: IR (thin film): 692, 731, 792, 901, 958, 1025, 1054. ^1H NMR (400 MHz, $CDCl_3$) δ 6.37 (dd, J = 176.5, 2.0 Hz, 1H). Characteristic peaks for the ^{15}N labelled compound: IR (thin film): 692, 731, 794, 910, 960, 1025. ^1H NMR (400 MHz, $CDCl_3$) δ 8.52 (d, J = 61.7 Hz, 1H, NH). ^{13}C NMR (101 MHz, $CDCl_3$) δ 160.4 (d, J = 9.6 Hz, C=N). ^{15}N NMR (41 MHz, CDCl3) δ 213.5. HRMS ($M+Na^+$): cald. for $C_{35}{}^{13}CH_{36}Cl_3NO_6Na$ 707.15340, found 707.15396 cald. for $C_{36}H_{36}Cl_3{}^{15}NO_6Na$ 707.14708, found 707.14744. Spectra in agreement with literature.[4]

3. ^{13}C / ^{15}N Exchange Experiment

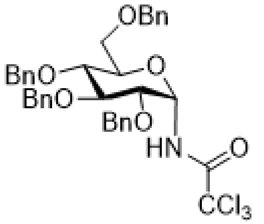

N-trichloroacetyl-2,3,4,6-tetra-O-benzyl-α-D-glucopyranosylamide (8)

Obtained after using the General Procedure for imidate transfer on both 2,3,4,6-tetra-O-benzyl-α-D-glucopyranosyl trichloroacetimidate and 2,3,4,6-tetra-O-benzyl-β-D-glucopyranosyl trichloroacetimidate. Flash column chromatography (5-15% EtOAc in pentane) yielded the title compound as a colourless oil (63 mg, 0.091 mmol, 60%, α/β > 20:1 for α donor; 48.8 mg, 0.071 mmol, 50%, α/β > 20:1 for β donor). Data reported for the α anomer: IR (thin film): 676, 695, 732, 819, 1067, 1453, 1495, 1728, 2869. ^{1}H NMR (400 MHz, Acetone) δ 8.46 (d, *J* = 7.7 Hz, 0.5H, ^{14}NH), 8.46 (ddd, *J* = 92.3, 7.7, 2.0 Hz, 0.5H, ^{15}NH), 7.48 – 7.24 (m, 20H, CH_{arom}), 5.87 (dd, *J* = 7.8, 5.5 Hz, 0.5H, ^{12}CH-1), 5.87 (ddd, *J* = 164.8, 7.8, 5.5 Hz, 0.5H, ^{13}CH-1), 4.93 (d, *J* = 11.3 Hz, 1H, CH_2 Bn), 4.85 (d, *J* = 11.0 Hz, 1H, CH_2 Bn), 4.80 (d, *J* = 11.3 Hz, 1H, CH_2 Bn), 4.75 (s, 2H, CH_2 Bn), 4.65 (d, *J* = 10.8 Hz, 1H, CH_2 Bn), 4.59 (d, *J* = 11.9 Hz, 1H, CH_2 Bn), 4.55 (d, *J* = 12.0 Hz, 1H, CH_2 Bn), 4.14 (t, *J* = 8.9 Hz, 1H, H-3), 3.97 (ddt, *J* = 9.2, 5.6, 1.7 Hz, 1H, H-2), 3.89 – 3.82 (m, 1H, H-5), 3.79 (dd, J = 11.0, 4.1 Hz, 1H, H-6), 3.71 (dd, J = 11.0, 1.9 Hz, 1H, H-6), 3.66 (dd, J = 9.8, 8.5 Hz, 1H, H-4). ^{13}C NMR (101 MHz, Acetone) δ 162.6 (C=O), 139.3, 138.8, 138.8, 138.2 ($C_{q,arom}$), 128.4, 128.3, 128.3, 128.1, 127.9, 127.8, 127.8, 127.7, 127.6, 127.4, 127.4 (CH_{arom}), 102.5, 95.8, 94.1, 81.4 (C-3), 77.5 (C-2), 76.9 (C-1, 14N), 76.9 (d, J = 10.2 Hz, C-1, ^{15}N), 76.8 (C-5), 76.8 (CH_2 Bn), 74.8 (CH_2 Bn), 74.6 (CH_2 Bn), 72.9 (CH_2 Bn), 72.4 (C-4), 68.4 (C-6). Inverse gated ^{13}C NMR (101 MHz, Tol) δ 76.8 (s, C-1, 14N), 76.8 (d, *J* = 10.1 Hz, C-1, ^{15}N).^{15}N NMR (51 MHz, Acetone) δ 108.6 (NH, ^{12}C), 108.6 (d, J = 10.2 Hz, NH, ^{13}C). HRMS ($M+NH_4^+$): cald. for $C_{36}H_{36}Cl_3NO_6NH_4$ 701.19465, found 701.19482 cald. for $C_{35}{}^{13}CH_{36}Cl_3NO_6NH_4$ 702.19800, found 702.19769 cald. for $C_{36}H_{36}Cl_3{}^{15}NO_6NH_4$ 702.19168, found 702.19116 cald. for $C_{35}{}^{13}CH_{36}Cl_3{}^{15}NO_6NH_4$ 703.19504, found 703.19369. Spectra in agreement with literature.[7]

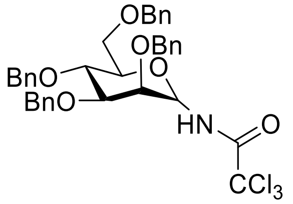

N-trichloroacetyl-2,3,4,6-tetra-O-benzyl-α/β-D-mannopyranosylamide (9)

Obtained after using the General Procedure for imidate transfer on 2,3,4,6-tetra-O-benzyl-α-D-mannopyranosyl trichloroacetimidate. Flash column chromatography (20% Et_2O in pentane) yielded the title compound as a colourless oil (77.4 mg, 0.12 mmol, 77%, α/β = 1 : 1.6). IR (thin film): 695, 732, 819, 1025, 1090, 1453, 1495, 1724, 2864. ^{1}H NMR (400 MHz, Acetone) δ 8.80 (d, *J* = 8.4 Hz, 0.5H, ^{14}NHα), 8.80 (dd, *J* = 91.5, 8.4 Hz, 0.5H, ^{15}NHα), 7.88 (d, *J* = 8.8 Hz, 0.75H, ^{14}NHβ), 7.88 (dd, *J* = 96.0, 8.8 Hz, 0.75H, ^{15}NHβ), 7.51 – 7.22 (m, 50H, CH_{arom}), 5.75 (ddd, *J* = 163.8, 8.4, 5.8 Hz, 0.5H, ^{13}CH-1α), 5.74 (dd, *J* = 8.4, 5.8 Hz, 0.5H, ^{12}CH-1α), 5.38 (ddd, *J* = 158.3, 8.9, 1.7 Hz, 0.75H, ^{13}CH-1β), 5.31 (dd, *J* = 8.9, 1.7 Hz, 0.75H, ^{12}CH-1β), 5.08 (d, *J* = 11.5 Hz, 1H, CH_2 Bn), 4.99 – 4.48 (m, 19H, CH_2 Bn), 4.25 (t, *J* = 2.0 Hz, 1.5H, H-2β), 4.14 (m, 2H, H-2α,), 4.11 – 4.03 (m, 1H), 4.01 (d, *J* = 1.5 Hz, 2H), 3.96 – 3.90 (m, 1H), 3.85 – 3.77 (m, 2H), 3.77 – 3.73 (m, 2.5H, H-6), 3.64 (dtt, *J* = 7.2, 3.0, 1.5 Hz, 1.5H, H-5β). ^{13}C NMR (101 MHz, Acetone) δ 162.6 (^{14}N-C=Oα), 162.5 (d, *J* = 20.9 Hz, ^{15}N-C=Oα), 161.4 (^{14}N-C=Oβ), 161.4 (d, *J* = 20.8 Hz, ^{15}N-C=Oβ), 139.7, 139.6, 139.5, 139.5, 139.4, 139.3 ($C_{q,\,arom}$), 129.4, 129.2, 129.1, 129.1, 129.0, 129.0, 129.0, 128.7, 128.7, 128.6, 128.6, 128.5, 128.4, 128.4, 128.4, 128.3, 128.2, 128.2, 128.1 (CH_{arom}), 83.8 (C-5α), 79.4 (d, *J* = 13.9 Hz, ^{15}NC-1β), 79.4 (^{14}NC-1β), 78.0 (d, *J* = 11.1 Hz, ^{15}NC-1α), 78.0 (^{14}NC-1α), 77.2 (C-5β), 76.0 (C-2β), 75.9 (C-2α), 75.8 (CH_2 Bn), 75.7 (C-3α), 75.3, 75.1, 73.7 (CH_2 Bn), 73.6 (CH_2 Bn), 73.4 (CH_2 Bn), 73.3 (CH_2 Bn), 72.9 (CH_2 Bn), 72.8 (CH_2 Bn), 69.9 (C-6), 69.8 (C-6). Inverse gated ^{13}C NMR (101 MHz, Tol) δ 78.6 (s, C-1, 14Nβ), 78.6 (d, *J* = 14.1 Hz, C-1, ^{15}Nβ), 77.4 (s, C-1, 14Nα), 77.4 (d, *J* = 10.9 Hz, C-1, ^{15}Nα). ^{15}N NMR (41 MHz, Acetone) δ 114.5 (^{12}CNα), 114.5 (d, *J* = 11 Hz, ^{13}CNα), 114.1 (^{12}CNβ), 114.1 (d, *J* = 14 Hz, ^{13}CNβ). HRMS ($M+NH_4^+$): cald. for $C_{36}H_{36}Cl_3NO_6NH_4$ 701.19458, found 701.19465 cald. for $C_{35}{}^{13}CH_{36}Cl_3NO_6NH_4$ 702.19800, found 702.19740 cald. for $C_{36}H_{36}Cl_3{}^{15}NO_6NH_4$ 702.19168, found 702.19101 cald. for $C_{35}{}^{13}CH_{36}Cl_3{}^{15}NO_6NH_4$ 703.19504, found 703.19351. Spectra in agreement with literature.[7]

4. Mode of Attack Determination

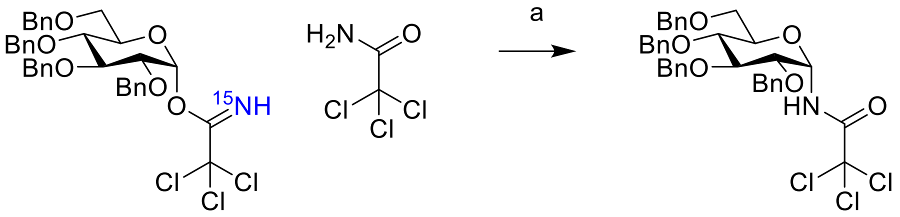

Scheme 6: Experiment to determine weather the nucleophile or the removed leaving group attacks. Reagents and conditions: a) TfOH (10 mol%), DCM, -20 °C.

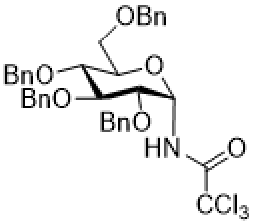

N-trichloroacetyl-2,3,4,6-tetra-O-benzyl-α-D-glucopyranosylamide (8)

A mixture of ^{15}N-2,3,4,6-tetra-O-benzyl-α-D-glucopyranosyl trichloroacetimidate (100 mg, 0.15 mmol, 1.0 eq) and unlabelled tricholoacetamide (24 mg, 0.15 mmol, 1.0 eq or 72 mg, 0.44 mmol, 3.0 eq) was co-evaporated with dry toluene and dried molsieves (3 Å, rods) were added. The oil was dissolved in dry DCM (3 mL) and cooled to -20 °C. TfOH (1.3 µL, 0.02 mmol, 0.1 eq) was added and the reaction mixture was stirred at -20 °C for 30 min. The reaction was quenched with solid $NaHCO_3$ and allowed to warm to room temperature. The suspension was filtered over cotton and the filtrate was washed twice with saturated aqueous $NaHCO_3$ and once with water, dried over Na_2SO_4 and concentrated *in vacuo*. Flash column chromatography (5-15% EtOAc in pentane) yielded the title compound as a colourless oil (40 mg, 0.058 mmol, 40%). Data for the experiment with 1 equivalent of additional trichloracetamide: ^{1}H NMR (400 MHz, Acetone) δ 8.45 (d, *J* = 7.7 Hz, 0.28H, ^{14}NH), 8.44 (dd, *J* = 93.1, 7.7 Hz, 0.72H, ^{15}NH), 7.43 – 7.22 (m, 20H, CH_{arom}), 5.86 (dd, *J* = 7.7, 5.6 Hz, 1H, H-1), 4.92 (d, *J* = 11.2 Hz, 1H, CH_2 Bn), 4.83 (d, *J* = 10.9 Hz, 1H, CH_2 Bn), 4.79 (d, *J* = 11.3 Hz, 1H, CH_2 Bn), 4.73 (s, 2H, CH_2 Bn), 4.64 (d, *J* = 10.9 Hz, 1H, CH_2 Bn), 4.58 (d, *J* = 12.0 Hz, 1H, CH_2 Bn), 4.53 (d, *J* = 12.0 Hz, 1H, CH_2 Bn), 4.13 (dd, *J* = 9.3, 8.5 Hz, 1H, H-3), 3.99 – 3.92 (m, 1H, H-2), 3.84 (ddd, *J* = 9.8, 4.1, 1.9 Hz, 1H, H-5), 3.77 (dd, *J* = 11.0, 4.1 Hz, 1H, H-6), 3.69 (dd, *J* = 11.0, 1.9 Hz, 1H, H-6), 3.65 (dd, *J* = 9.9, 8.5 Hz, 1H, H-4). ^{13}C NMR (101 MHz, Acetone) δ 163.0 (^{14}N-C=O), 163.0 (d, *J* = 19.4 Hz, ^{15}N-C=O), 140.0, 139., 139.5, 138.9 ($C_{q,\ arom}$), 129.2, 129.0, 129.0, 128.8, 128.7, 128.6, 128.6, 128.5, 128.3, 128.2, 128.2 (CH_{arom}), 81.9 (C-3), 79.1 (C-2), 78.3 (C-4), 77.7 (^{14}NC-1), 77.7 (d, *J* = 10.1 Hz, ^{15}NC-1) 75.6 (CH_2 Bn), 75.4 (CH_2 Bn), 73.7 (CH_2 Bn), 73.2 (CH_2 Bn), 73.0 (C-5), 69.7 (C-6). ^{15}N NMR (41 MHz, Acetone) δ 110.4. Characteristic peaks for the experiment with 3 equivalents of trichloroacetamide: ^{1}H NMR (400 MHz, Acetone) δ 8.45 (d, *J* = 7.7 Hz, 0.30H, ^{14}NH), 8.44 (dd, *J* = 92.2, 7.7 Hz, 0.70H, ^{15}NH). HRMS ($M+NH_4^+$): cald. for $C_{36}H_{36}Cl_3NO_6NH_4$ 701.19458, found 701.19465 cald. for $C_{36}H_{36}Cl_3{}^{15}NO_6NH_4$ 702.19168, found 702.19101. Spectra in agreement with literature.[7]

5. Reversibility Experiment

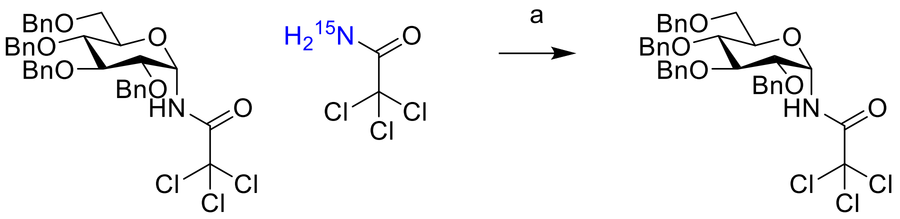

Scheme 7: Reaction used to determine the reversibility of the imidate transfer. Reagents and conditions: a) TfOH (10 mol%), DCM, -20 °C.

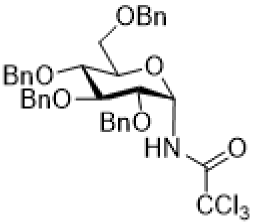

N-trichloroacetyl-2,3,4,6-tetra-O-benzyl-α-D-glucopyranosylamide (8)

A mixture of N-trichloroacetyl-2,3,4,6-tetra-O-benzyl-α-D-glucopyranosylamide (100 mg, 0.15 mmol, 1.0 eq) and ^{15}N trichloroacetamide (24 mg, 0.15 mmol, 1.0 eq) was co-evaporated with dry toluene and dissolved in dry DCM (3 mL). Dried molsieves (3 Å, rods) were added and the reaction mixture was cooled to -20 °C. TfOH (1.3 µL, 0.02 mmol, 0.1 eq) was added and the reaction was stirred at -20 °C for 30 min. The reaction was quenched with solid $NaHCO_3$ and filtered over cotton. The filtrate was washed twice with saturated aqueous $NaHCO_3$ and once with water, dried over Na_2SO_4 and concentrated *in vacuo*. Flash column chromatography (5-15% EtOAc in pentane) yielded the title compound as a colourless oil (54 mg, 0.081 mmol, 55%). No ^{15}N incorporation was observed. ^{1}H NMR (400 MHz, Acetone) δ 8.45 (d, *J* = 7.7 Hz, 1H, NH), 7.43 – 7.21 (m, 20H, CH_{arom}), 5.87 (dd, *J* = 7.8, 5.6 Hz, 1H, h-1), 4.93 (d, *J* = 11.2 Hz, 1H, CH_2 Bn), 4.84 (d, *J* = 10.9 Hz, 1H, CH_2 Bn), 4.79 (d, *J* = 11.2 Hz, 1H, CH_2 Bn), 4.73 (s, 2H, CH_2 Bn), 4.64 (d, *J* = 10.9 Hz, 1H, CH_2 Bn), 4.58 (d, *J* = 12.0 Hz, 1H, CH_2 Bn), 4.53 (d, *J* = 11.9 Hz, 1H, CH_2 Bn), 4.14 (t, *J* = 8.9 Hz, 1H, H-3), 3.95 (dd, *J* = 9.3, 5.6 Hz, 1H, H-2), 3.84 (ddd, *J* = 9.9, 4.1, 1.9 Hz, 1H, H-5), 3.78 (dd, *J* = 11.0, 4.1 Hz, 1H, H-6), 3.70 (dd, *J* = 11.0, 2.0 Hz, 1H, H-6), 3.65 (dd, *J* = 9.9, 8.6 Hz, 1H, H-4). ^{13}C NMR (101 MHz, Acetone) δ 163.0 (N-C=O), 140.0, 139.5, 139.5, 138.9 ($C_{q,\ arom}$), 129.1, 129.0, 129.0, 128.8, 128.7, 128.6, 128.6, 128.5, 128.3, 128.2, 128.2 (CH_{arom}), 81.9 (C-3), 79.1 (C-2), 78.2 (C-4), 77.7 (C-1), 75.6 (CH_2 Bn), 75.3 (CH_2 Bn), 73.7 (CH_2 Bn), 73.2 (CH_2 Bn), 73.0 (C-5), 69.7 (C-6). HRMS ($M+NH_4^+$): cald. for $C_{36}H_{36}Cl_3NO_6NH_4$ 701.19458, found 701.19465. Spectra in agreement with literature.[7]

6. References

1. van der Vorm, S.; Hansen, T.; Overkleeft, H. S.; van der Marel, G. A.; Codée, J. D. C. The Influence of Acceptor Nucleophilicity on the Glycosylation Reaction Mechanism. *Chem Sci* **2017**, *8* (3), 1867–1875. DOI:10.1039/c6sc04638j.
2. Dinkelaar, J.; de Jong, A. R.; van Meer, R.; Somers, M.; Lodder, G.; Overkleeft, H. S.; Codée, J. D. C.; van der Marel, G. A. Stereodirecting Effect of the Pyranosyl C-5 Substituent in Glycosylation Reactions. *Journal of Organic Chemistry* **2009**, *74* (14), 4982–4991. DOI:10.1021/jo900662v.
3. Wang, J.; Zhao, Y.; Zhao, W.; Wang, P.; Li, J. Total Synthesis of N-Butyl-1-Deoxynojirimycin. *J Carbohydr Chem* **2016**, *35* (8–9), 445–454. DOI:10.1080/07328303.2017.1330415.
4. Chatterjee, S.; Moon, S.; Hentschel, F.; Gilmore, K.; Seeberger, P. H. An Empirical Understanding of the Glycosylation Reaction. *J Am Chem Soc* **2018**, *140* (38), 11942–11953. DOI:10.1021/jacs.8b04525.
5. Rahkila, J.; Ekholm, F. S.; Panchadhayee, R.; Ardá, A.; Cañada, F. J.; Jiménez-Barbero, J.; Leino, R. Synthesis and Conformational Analysis of Phosphorylated β-(1→2) Linked Mannosides. *Carbohydr Res* **2014**, *383*, 58–68. DOI:10.1016/j.carres.2013.10.017.
6. Heuckendorff, M.; Poulsen, L. T.; Hedberg, C.; Jensen, H. H. Dissection of the Effects That Govern Thioglucoside and Thiomannoside Reactivity. *Org Biomol Chem* **2018**, *16* (13), 2277–2288. DOI:10.1039/c7ob02968c.
7. Larsen, K.; Olsen, C. E.; Motawia, M. S. Acid-Catalysed Rearrangement of Glycosyl Trichloroacetimidates: A Novel Route to Glycosylamines. *Carbohydr Res* **2008**, *343* (2), 383–387. DOI:10.1016/j.carres.2007.10.019.

7. Spectral Data

^{1}H NMR of ^{15}N Trichloroacetamide (3) (400 MHz, Acetone-d6)

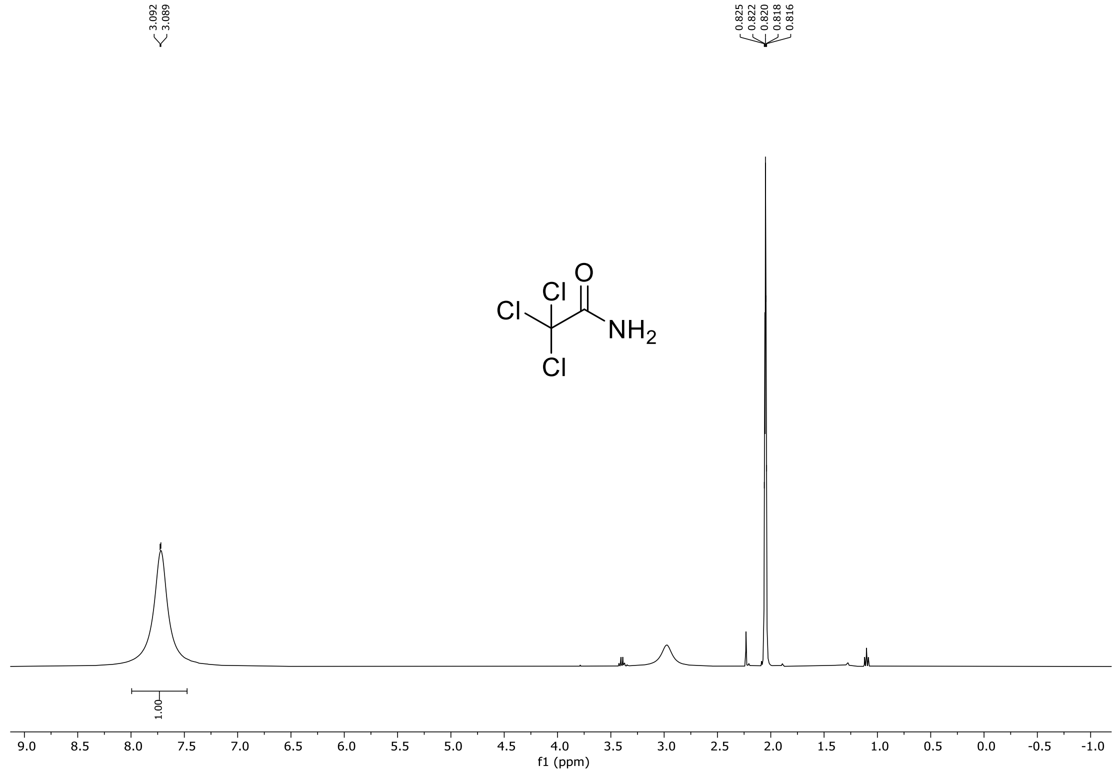

^{13}C NMR of ^{15}N Trichloroacetamide (3) (101 MHz, Acetone-d6)

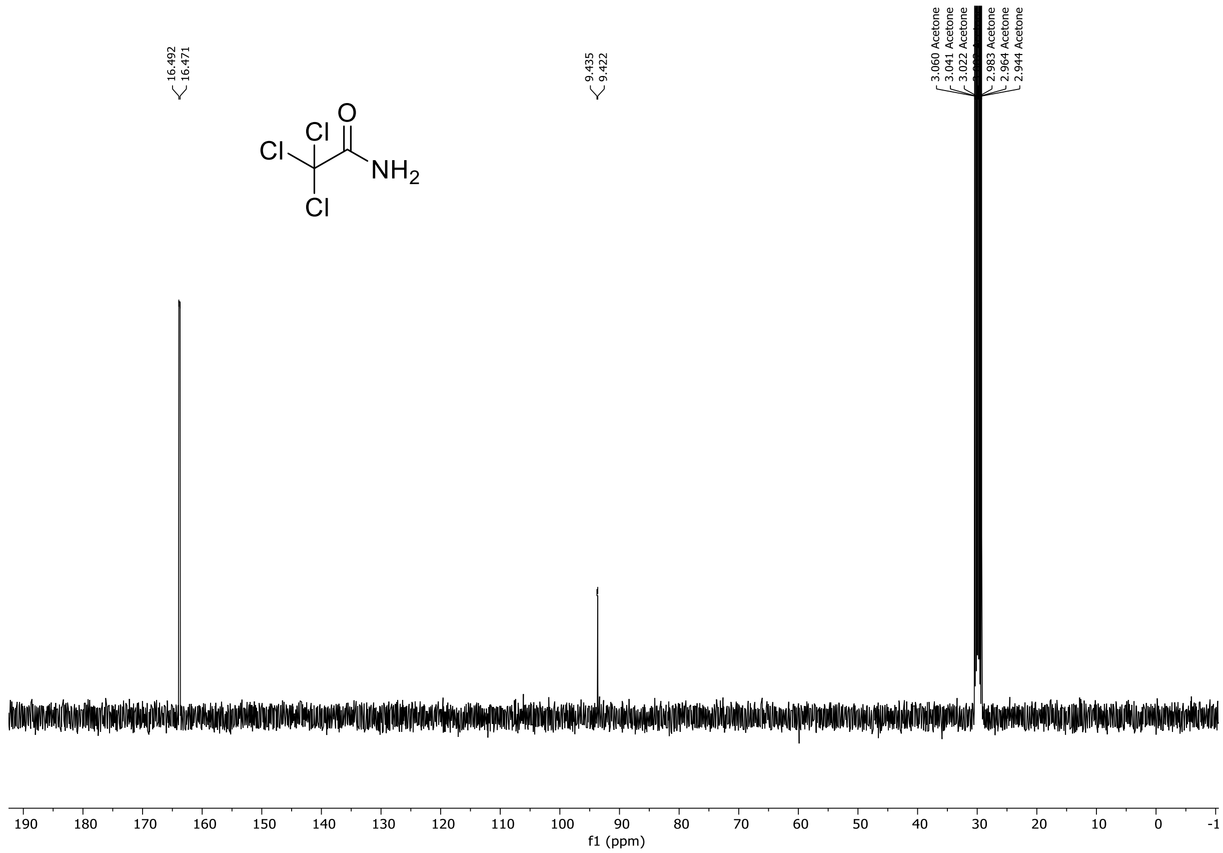

^{15}N NMR of ^{15}N Trichloroacetamide (3) (41 MHz, Acetone-d6)

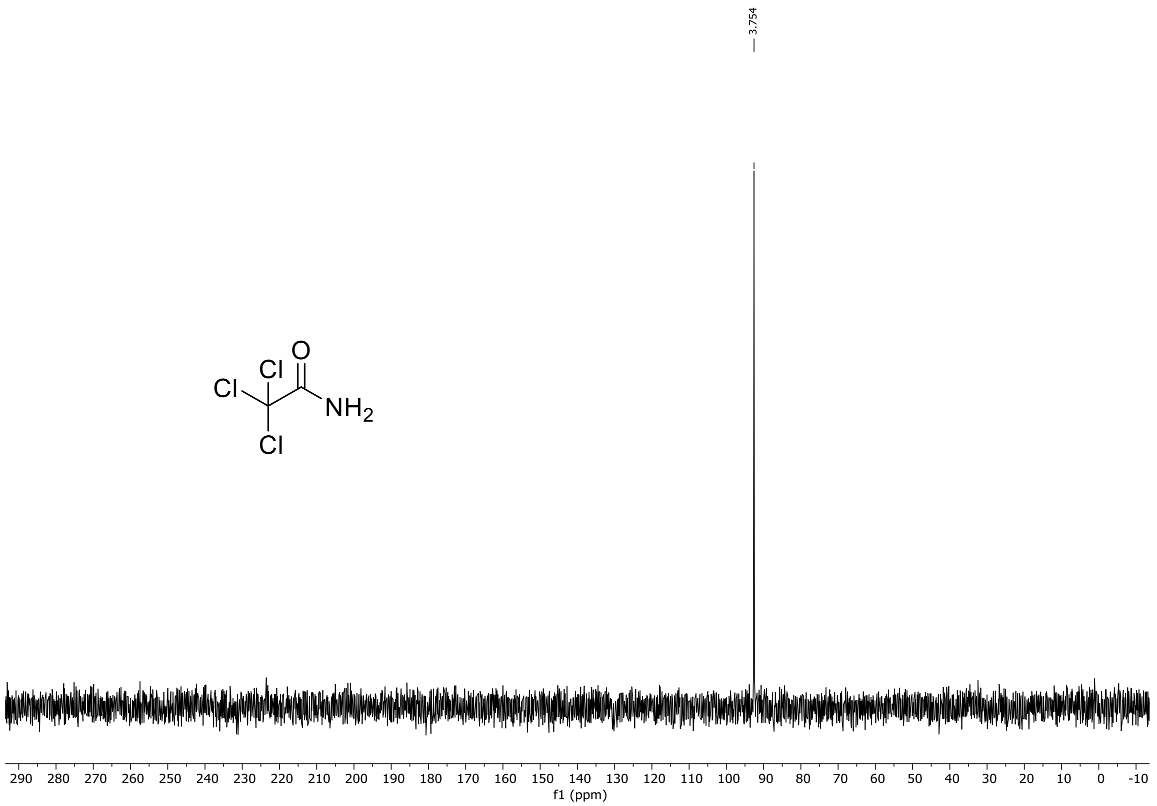

^{13}C NMR of ^{15}N Trichloroacetonitrile (4) (126 MHz, $CDCl_3$)

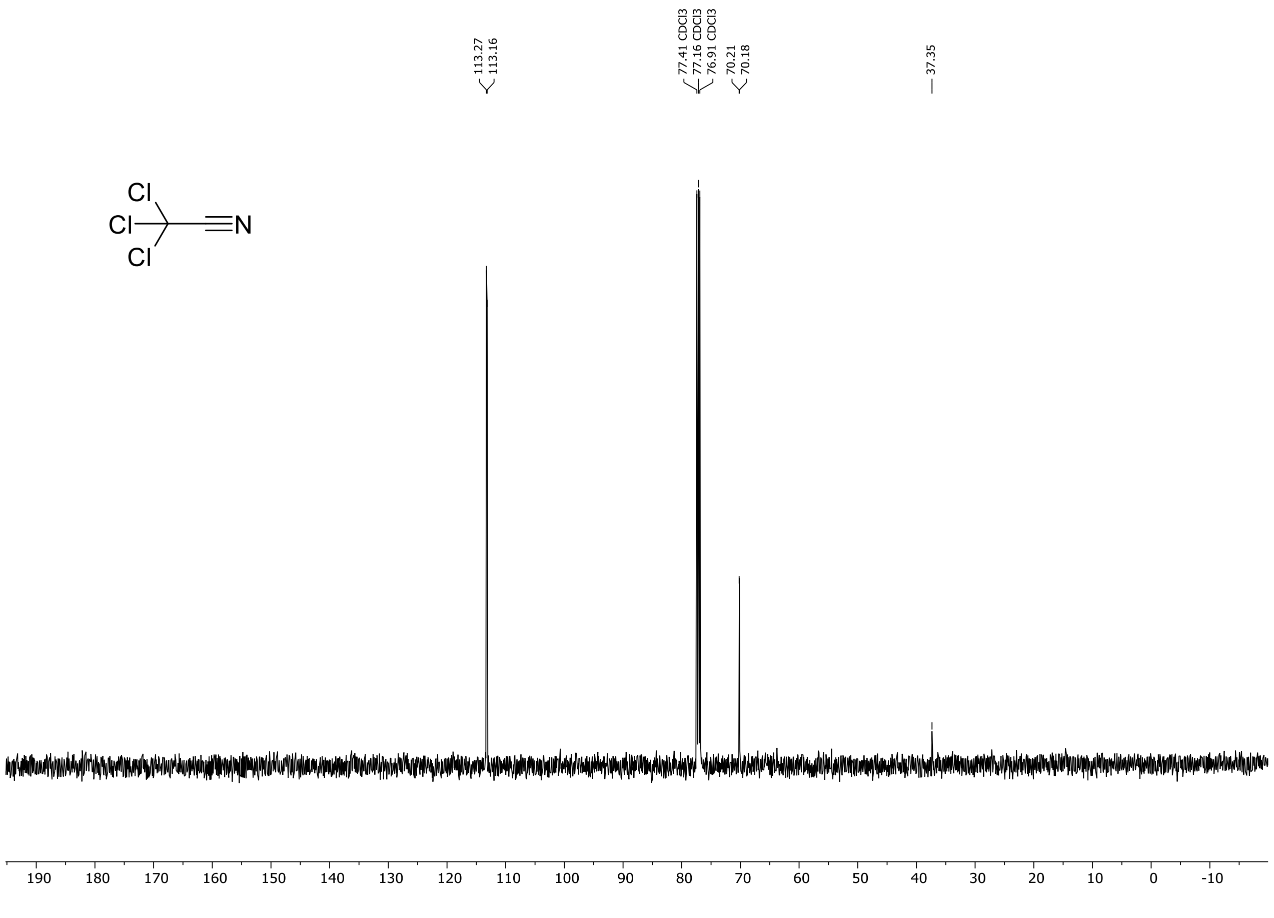

^{15}N NMR of ^{15}N Trichloroacetonitrile (4) (51 MHz, $CDCl_3$)

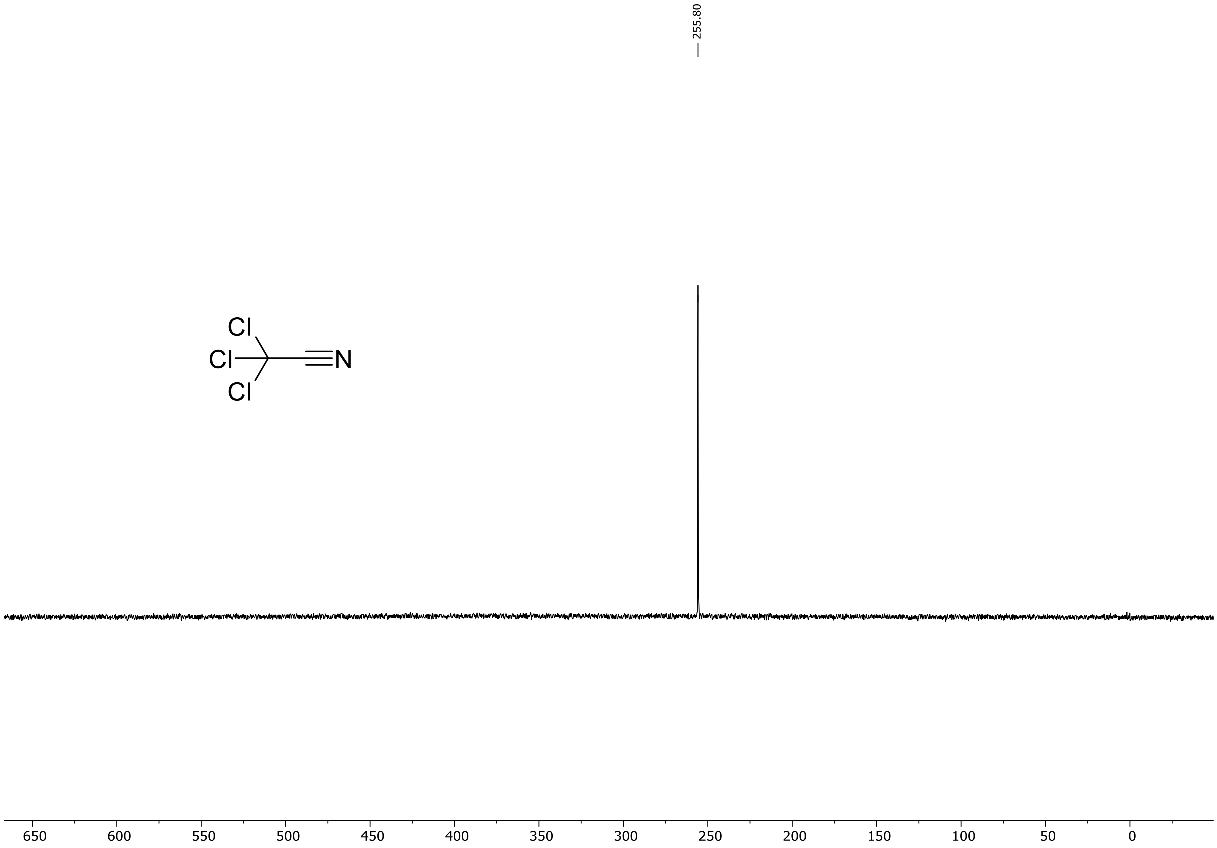

^{1}H NMR of Phenyl 2,3,4,6-tetra-O-benzyl-1-thio-β-D-glucospyranoside (S1) (400 MHz, $CDCl_3$)

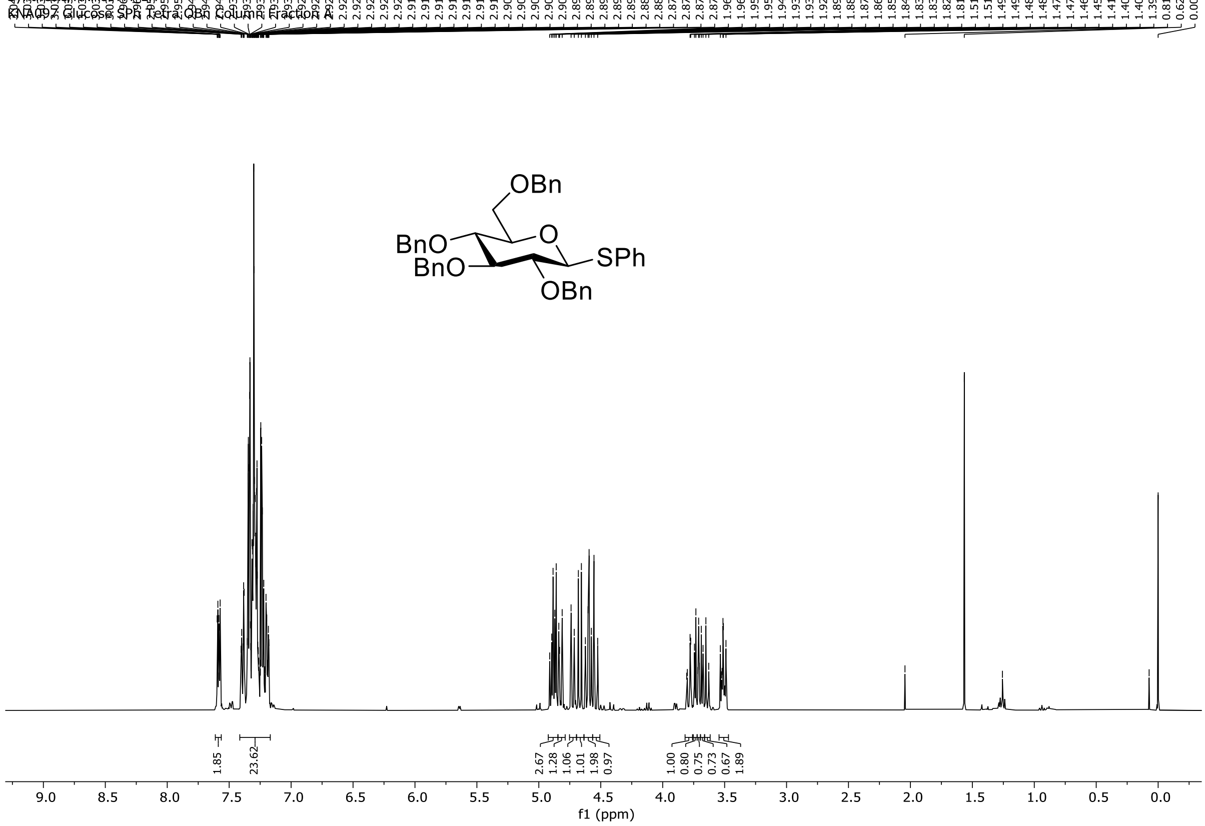

^{13}C NMR of Phenyl 2,3,4,6-tetra-O-benzyl-1-thio-β-D-glucospyranoside (S1) (101 MHz, CDCl$_3$)

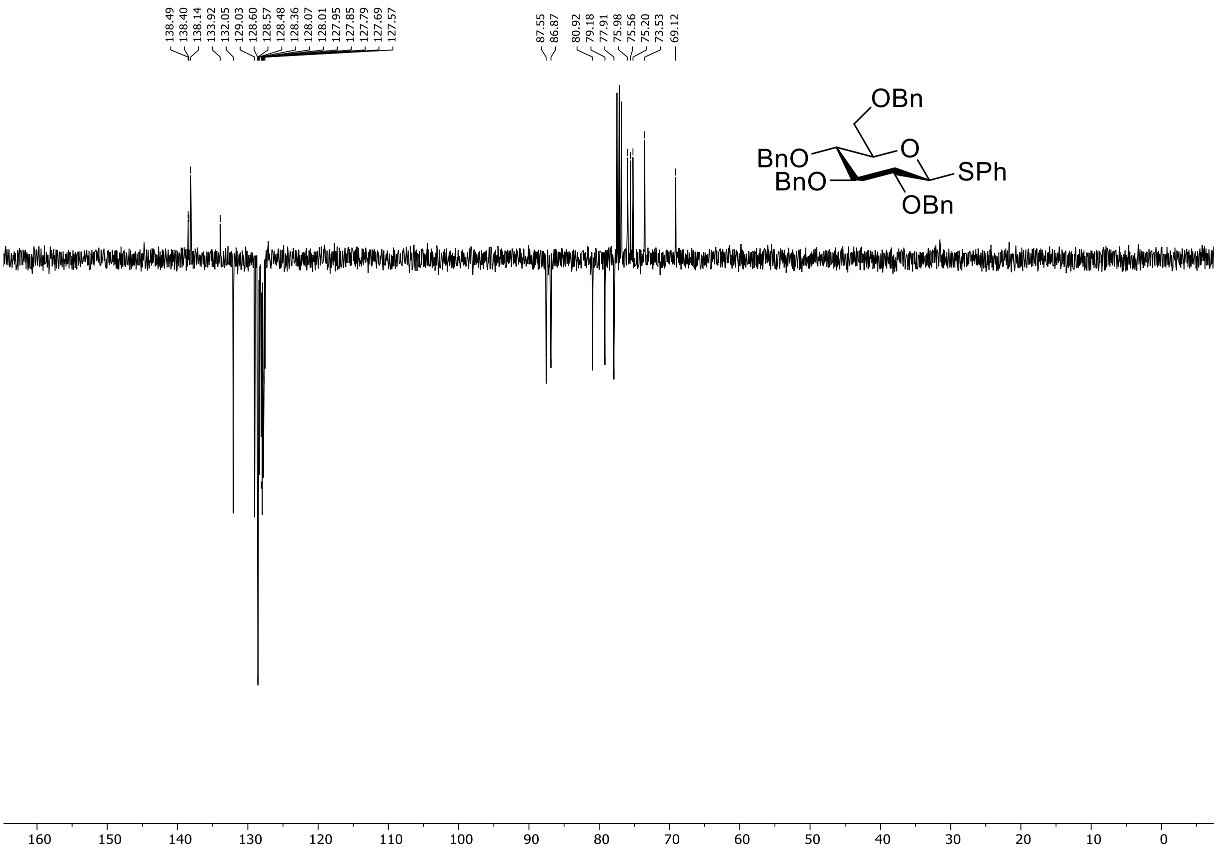

^{1}H NMR of 2,3,4,6-tetra-O-benzyl-D-glucospyranoside (S2) (400 MHz, CDCl$_3$)

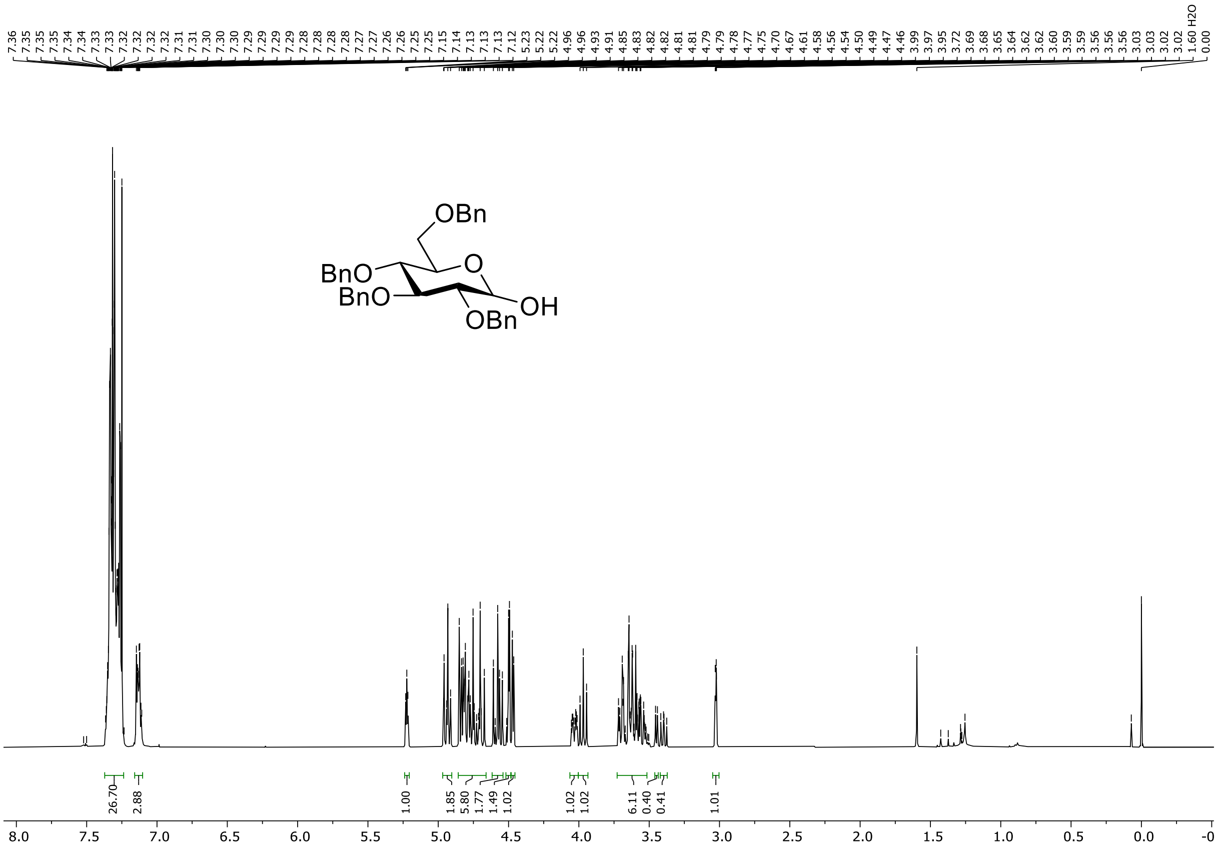

^{13}C NMR of 2,3,4,6-tetra-O-benzyl-D-glucospyranoside (S2) (101 MHz, CDCl$_3$)

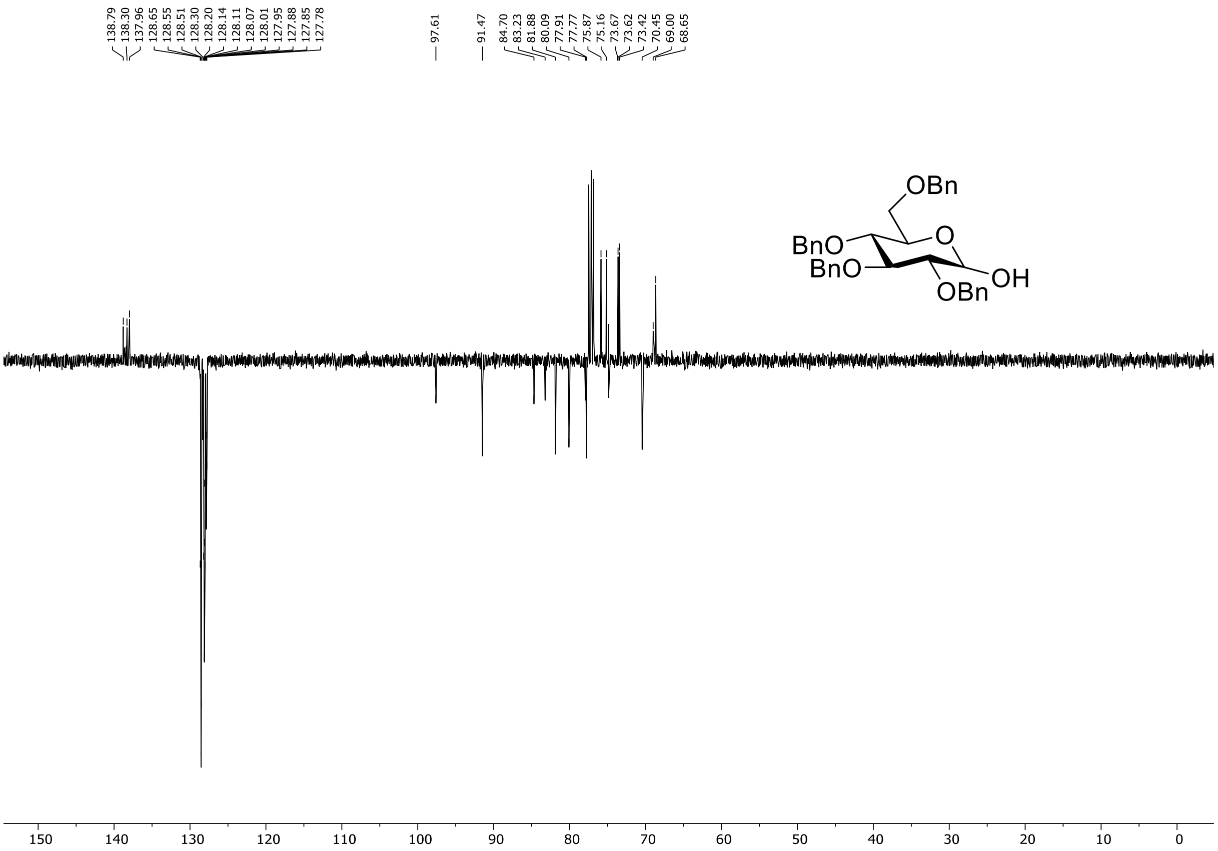

HH COSY of 2,3,4,6-tetra-O-benzyl-D-glucospyranoside (S2) (CDCl$_3$)

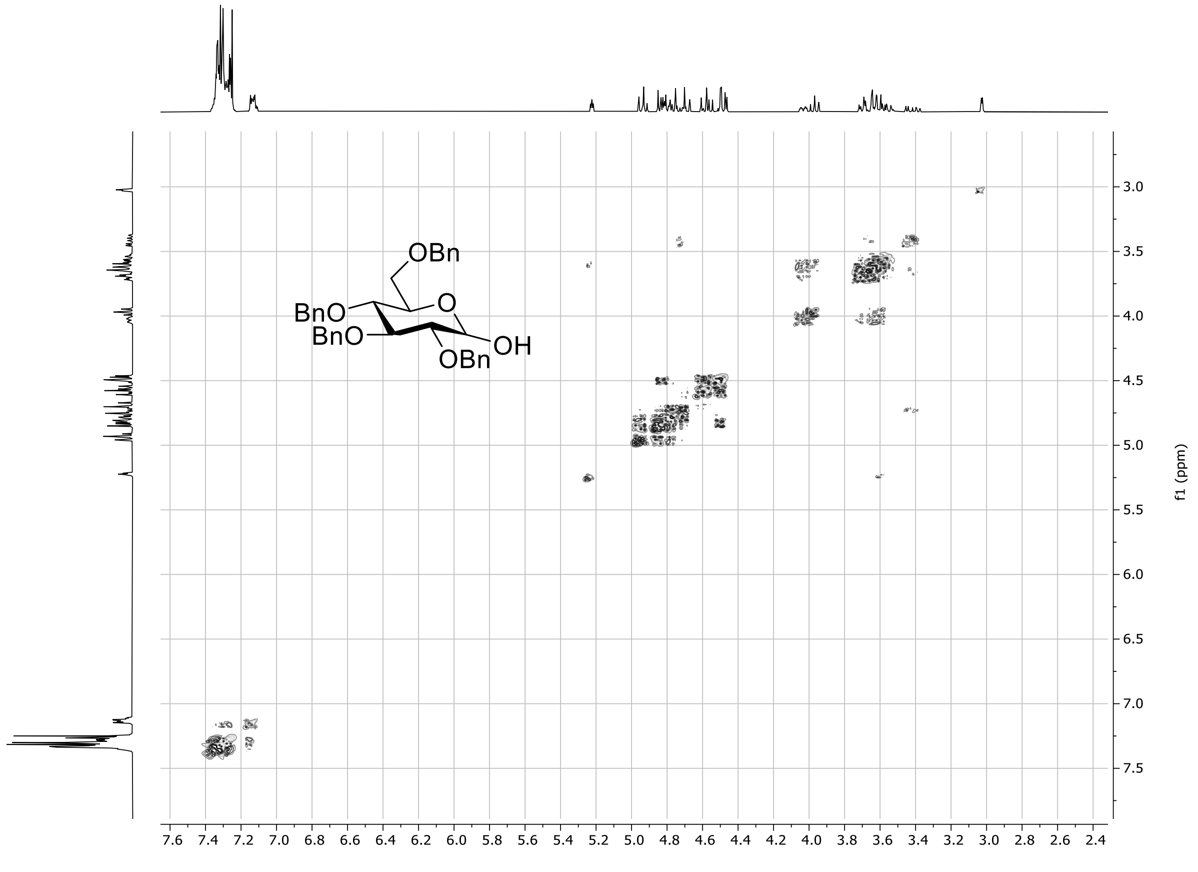

HC HSQC of 2,3,4,6-tetra-O-benzyl-D-glucospyranoside (S2) (CDCl$_3$)

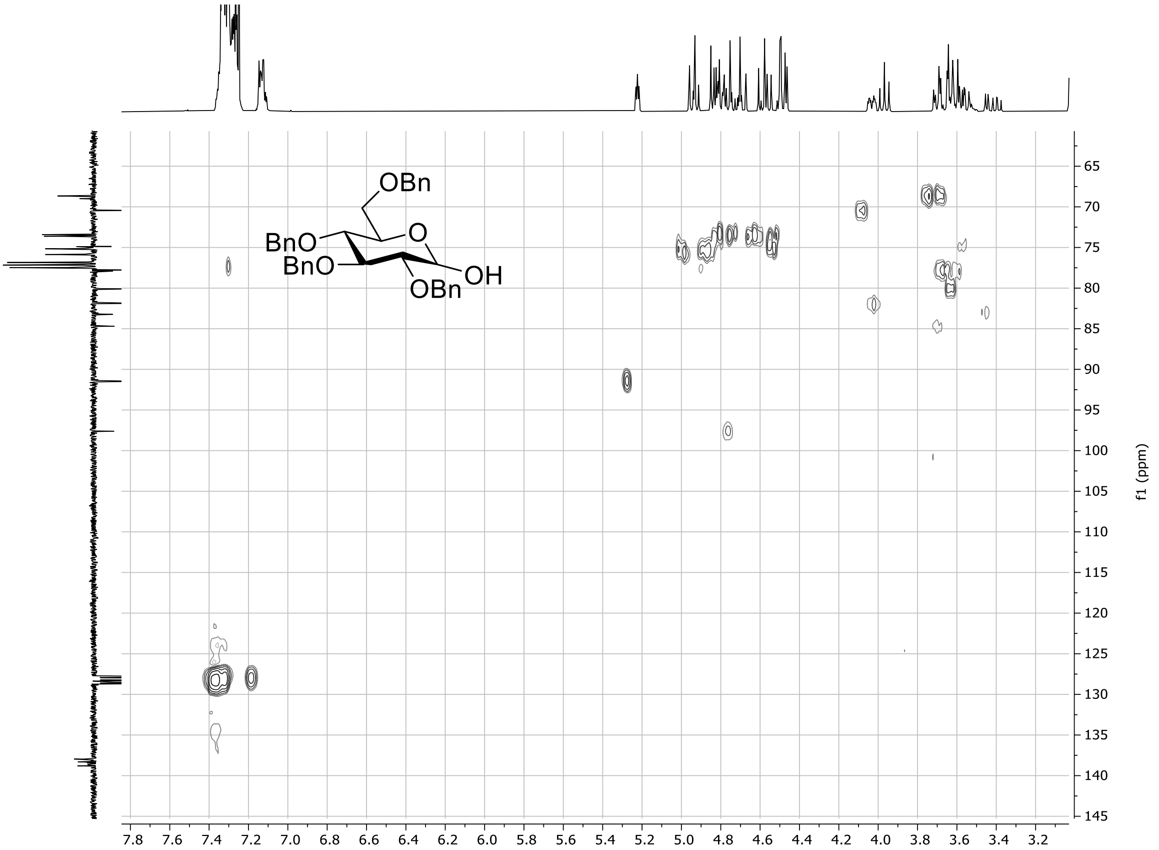

^{1}H NMR of 2,3,4,6-tetra-O-benzyl-α-D-glucopyranosyl trichloroacetimidate (5) (400 MHz, CDCl$_3$)

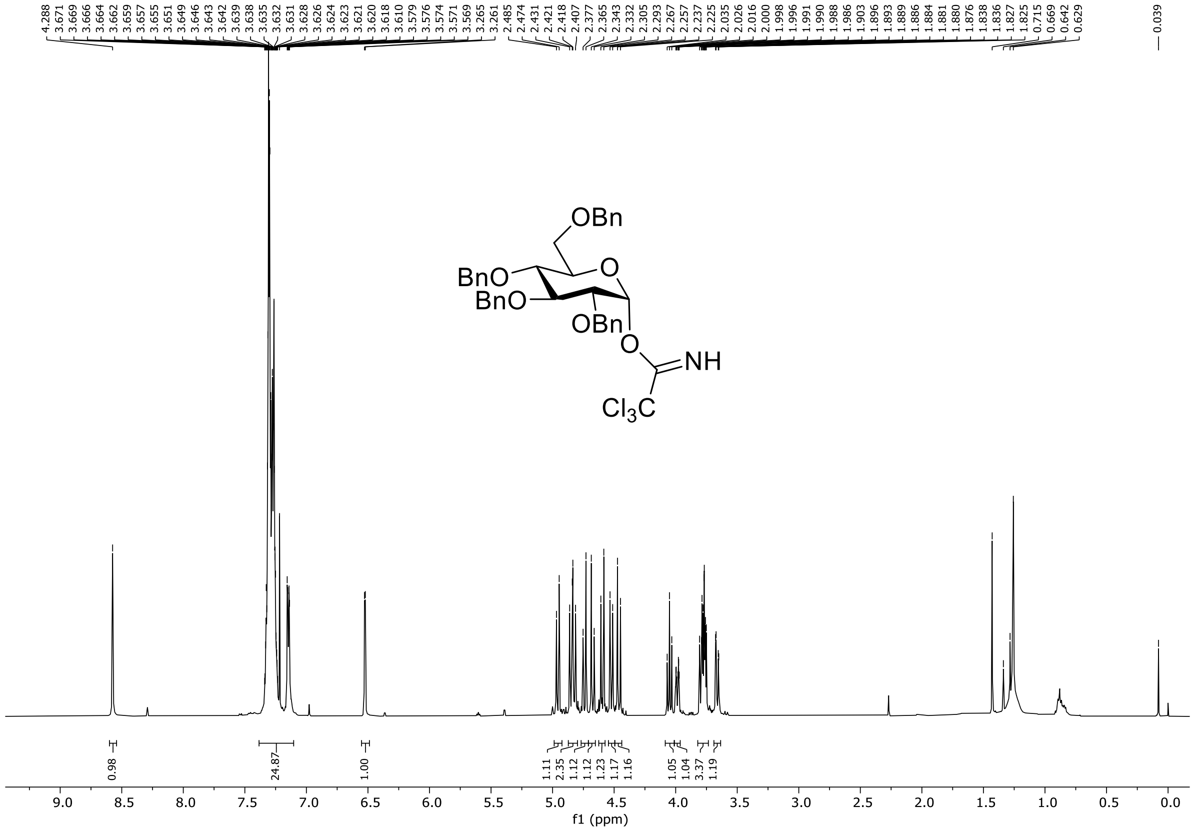

^{13}C NMR of 2,3,4,6-tetra-O-benzyl-α-D-glucopyranosyl trichloroacetimidate (5) (101 MHz, $CDCl_3$)

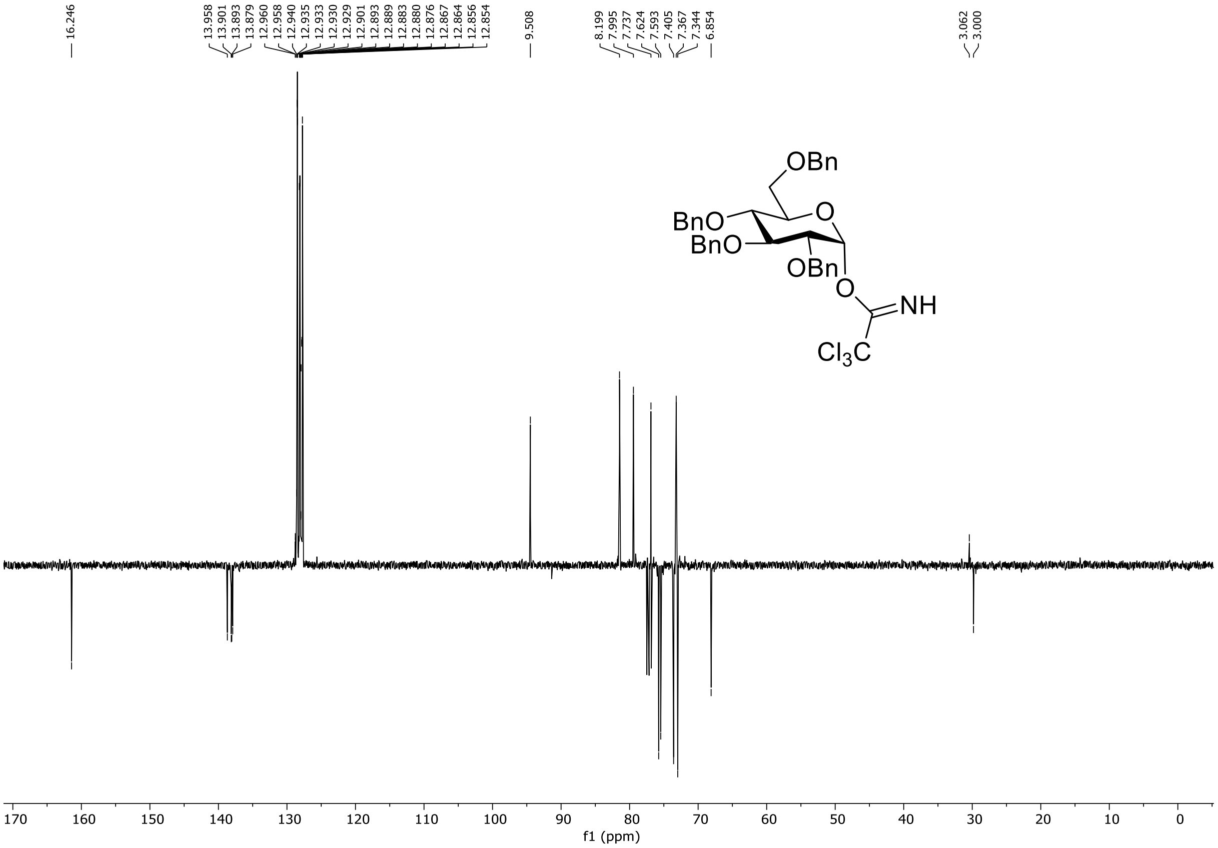

HH COSY of 2,3,4,6-tetra-O-benzyl-α-D-glucopyranosyl trichloroacetimidate (5) ($CDCl_3$)

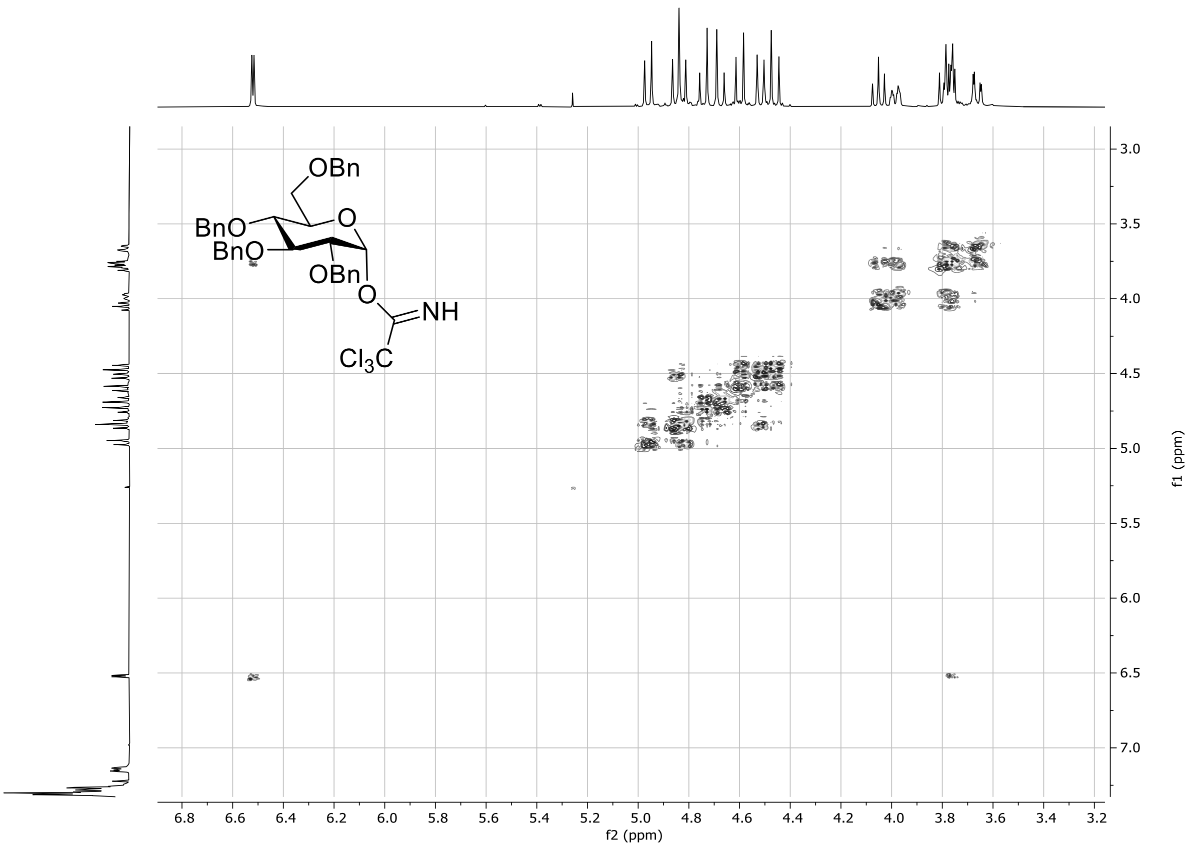

CH HSQC of 2,3,4,6-tetra-O-benzyl-α-D-glucopyranosyl trichloroacetimidate (5) ($CDCl_3$)

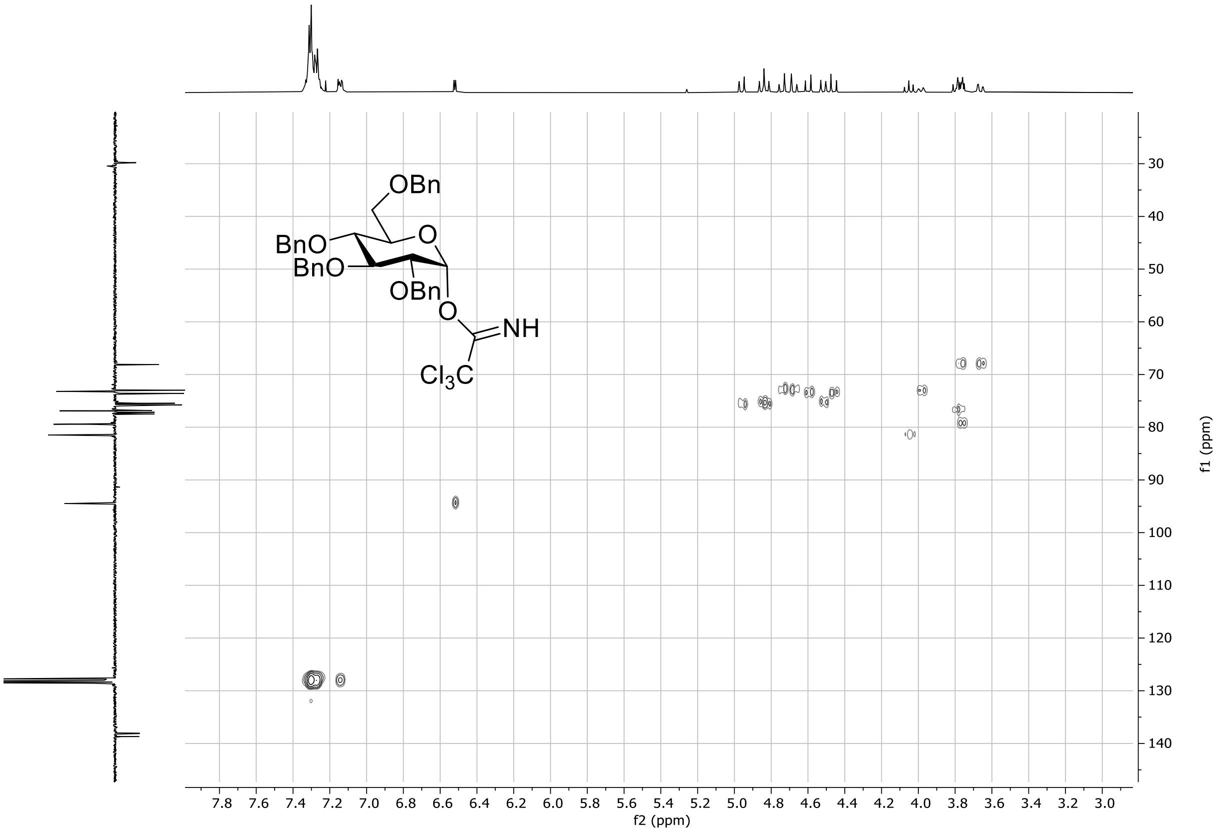

^{1}H NMR of Anomeric ^{13}C 2,3,4,6-tetra-O-benzyl-α-D-glucopyranosyl trichloroacetimidate (5) (400 MHz, $CDCl_3$)

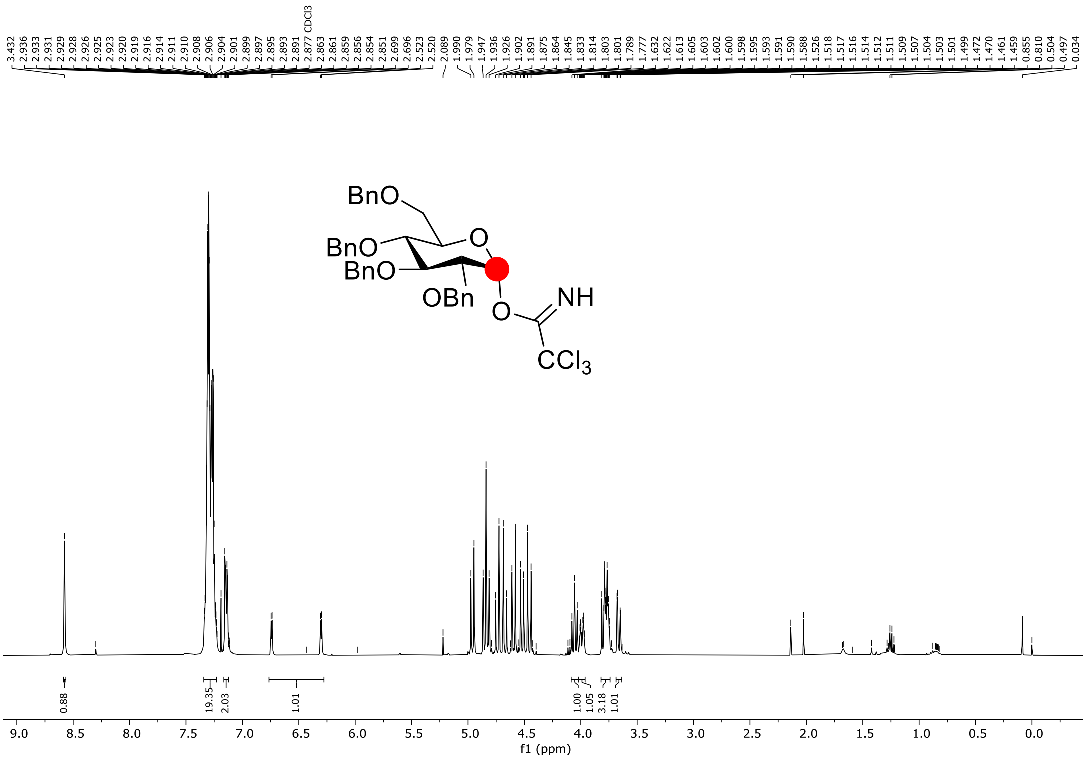

^{13}C NMR of Anomeric ^{13}C 2,3,4,6-tetra-O-benzyl-α-D-glucopyranosyl trichloroacetimidate (5) (101 MHz, $CDCl_3$)

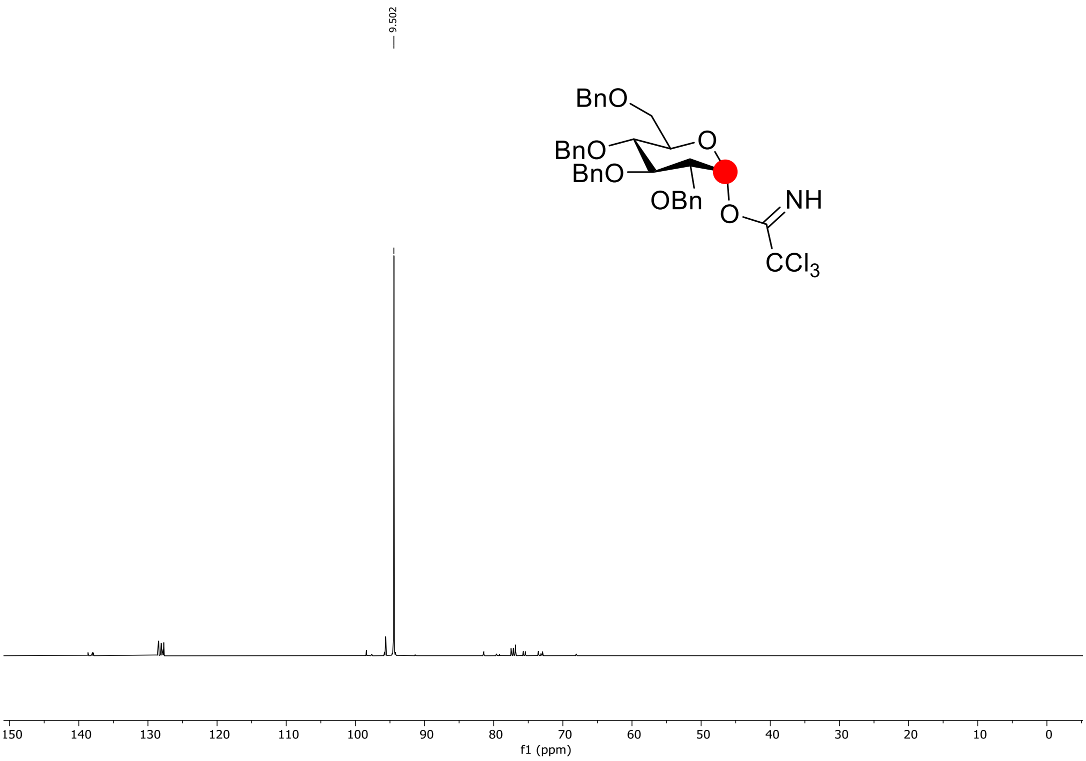

^{1}H NMR of ^{15}N 2,3,4,6-tetra-O-benzyl-α-D-glucopyranosyl trichloroacetimidate (5) (400 MHz, $CDCl_3$)

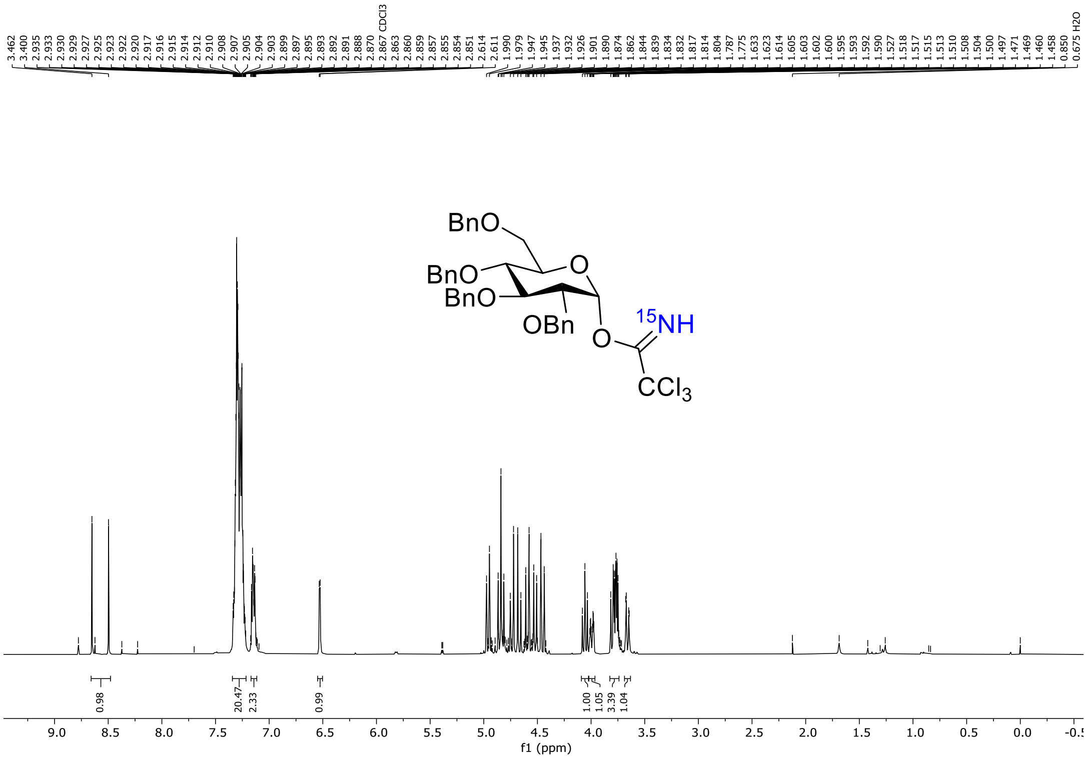

^{13}C NMR of ^{15}N 2,3,4,6-tetra-O-benzyl-α-D-glucopyranosyl trichloroacetimidate (5) (101 MHz, $CDCl_3$)

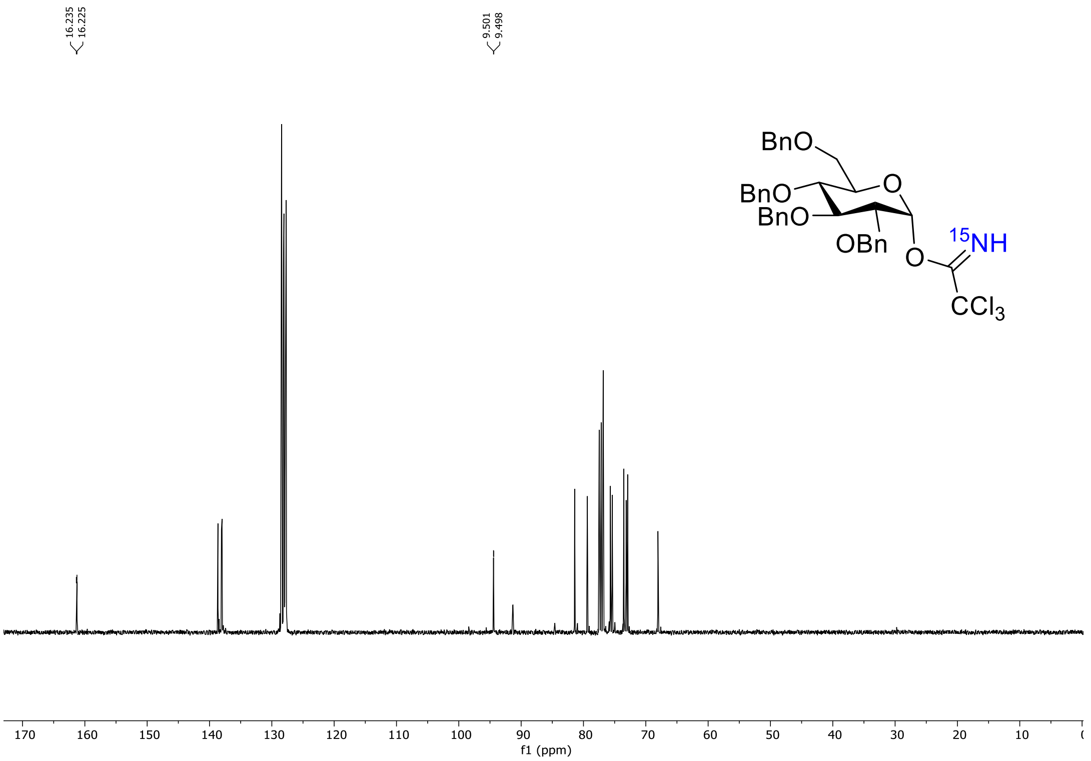

^{15}N NMR of ^{15}N 2,3,4,6-tetra-O-benzyl-α-D-glucopyranosyl trichloroacetimidate (5) (41 MHz, $CDCl_3$)

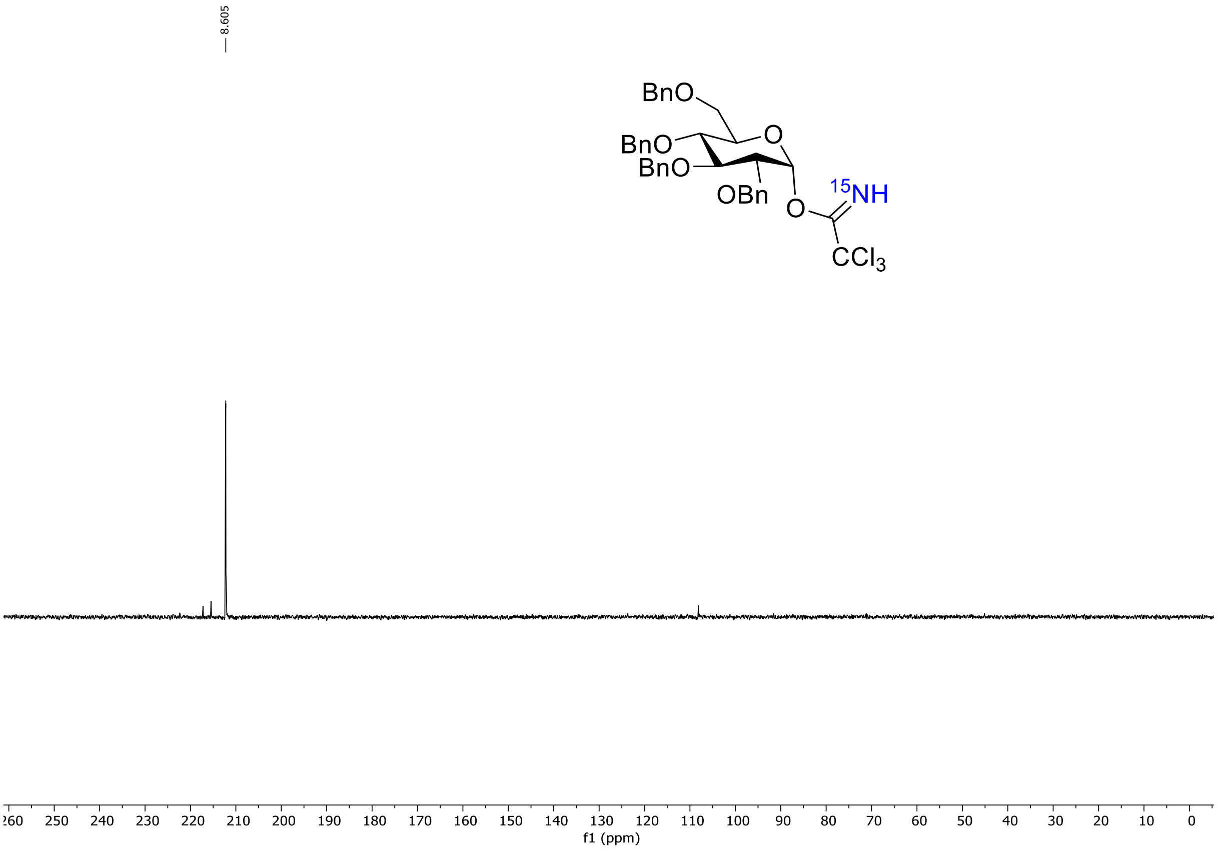

^{1}H NMR of 2,3,4,6-tetra-O-benzyl-β-D-glucopyranosyl trichloroacetimidate (6) (500 MHz, CDCl$_3$)

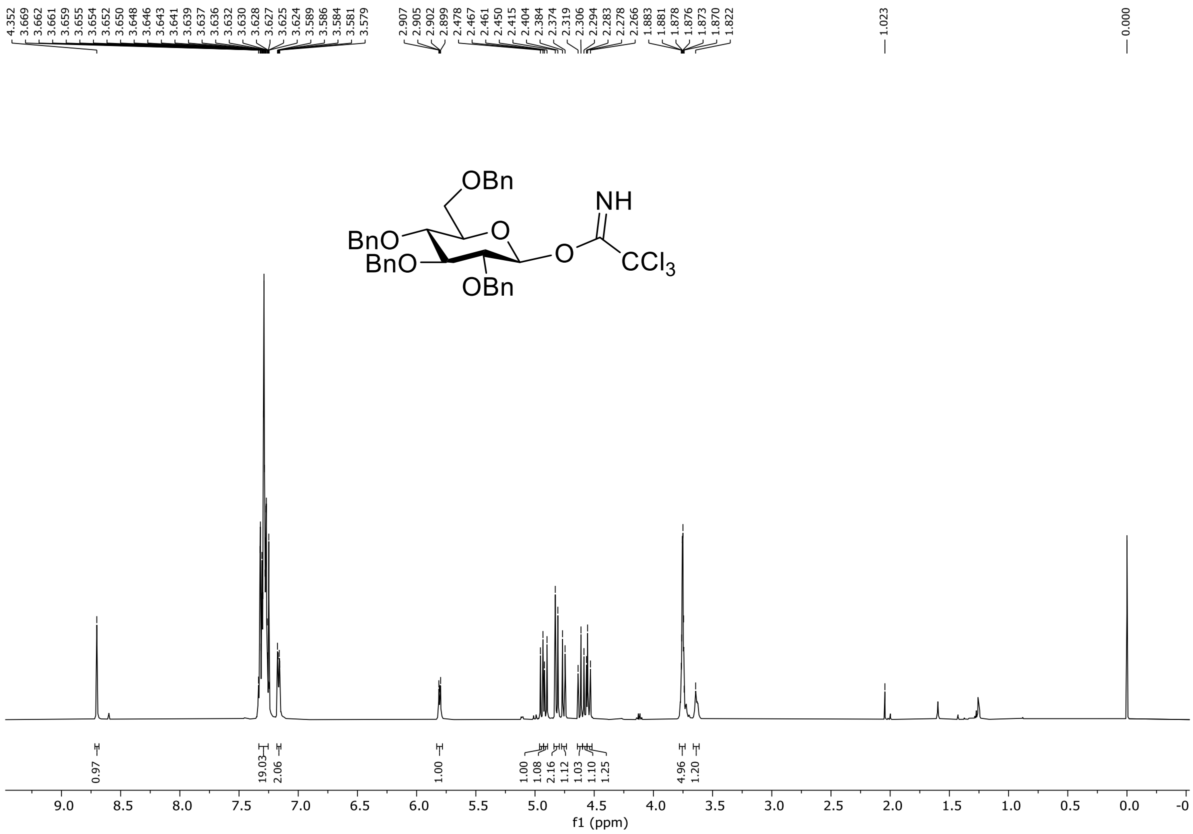

^{13}C NMR of 2,3,4,6-tetra-O-benzyl-β-D-glucopyranosyl trichloroacetimidate (6) (126 MHz, CDCl$_3$)

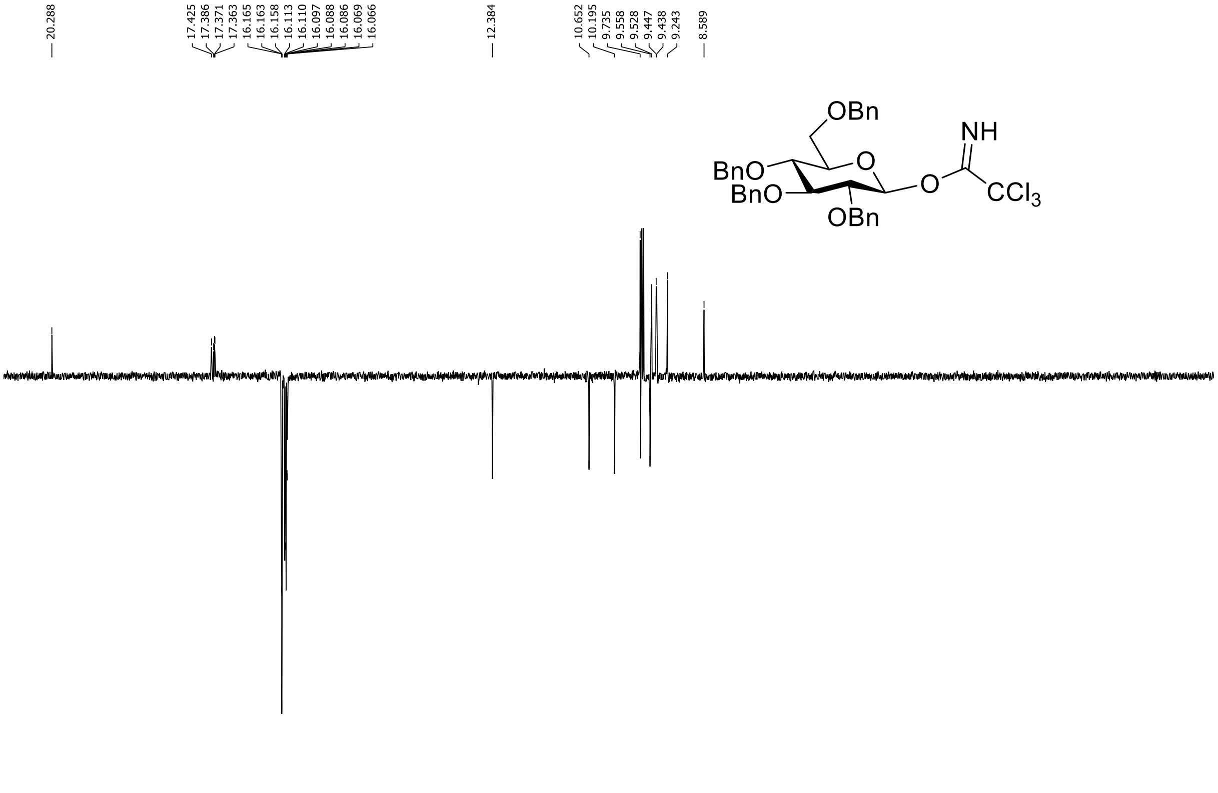

HH COSY of 2,3,4,6-tetra-O-benzyl-β-D-glucopyranosyl trichloroacetimidate (6) (CDCl₃)

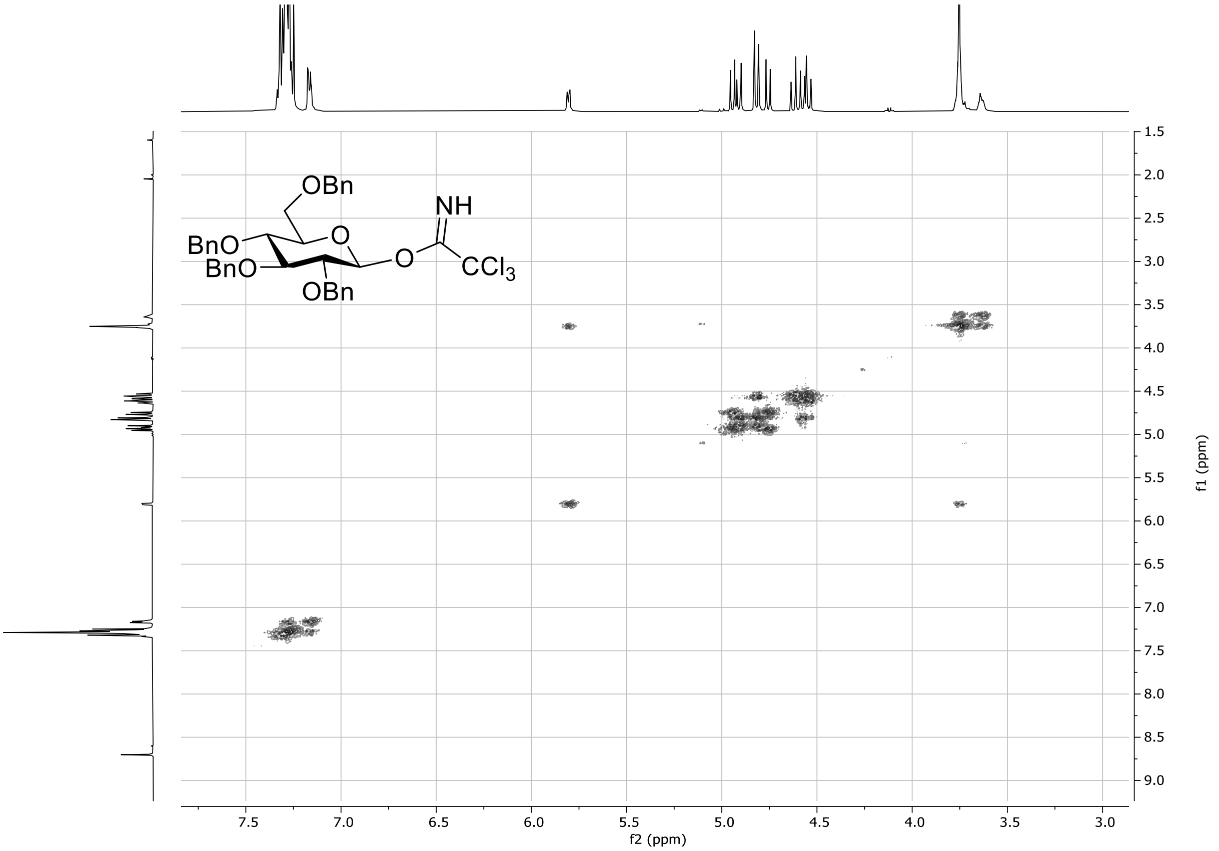

CH HSQC of 2,3,4,6-tetra-O-benzyl-β-D-glucopyranosyl trichloroacetimidate (6) (CDCl₃)

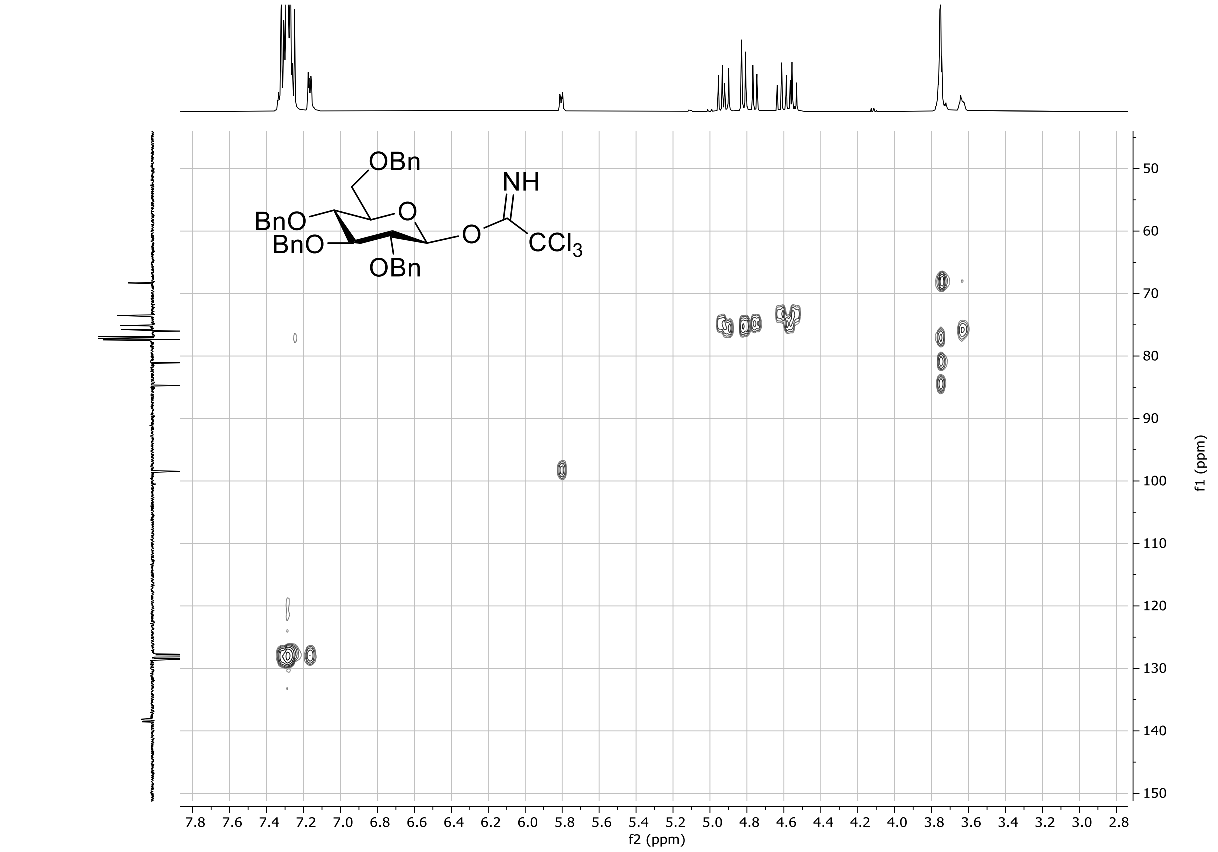

^{1}H NMR of anomeric ^{13}C 2,3,4,6-tetra-O-benzyl-β-D-glucopyranosyl trichloroacetimidate (6) (400 MHz, CDCl$_3$)

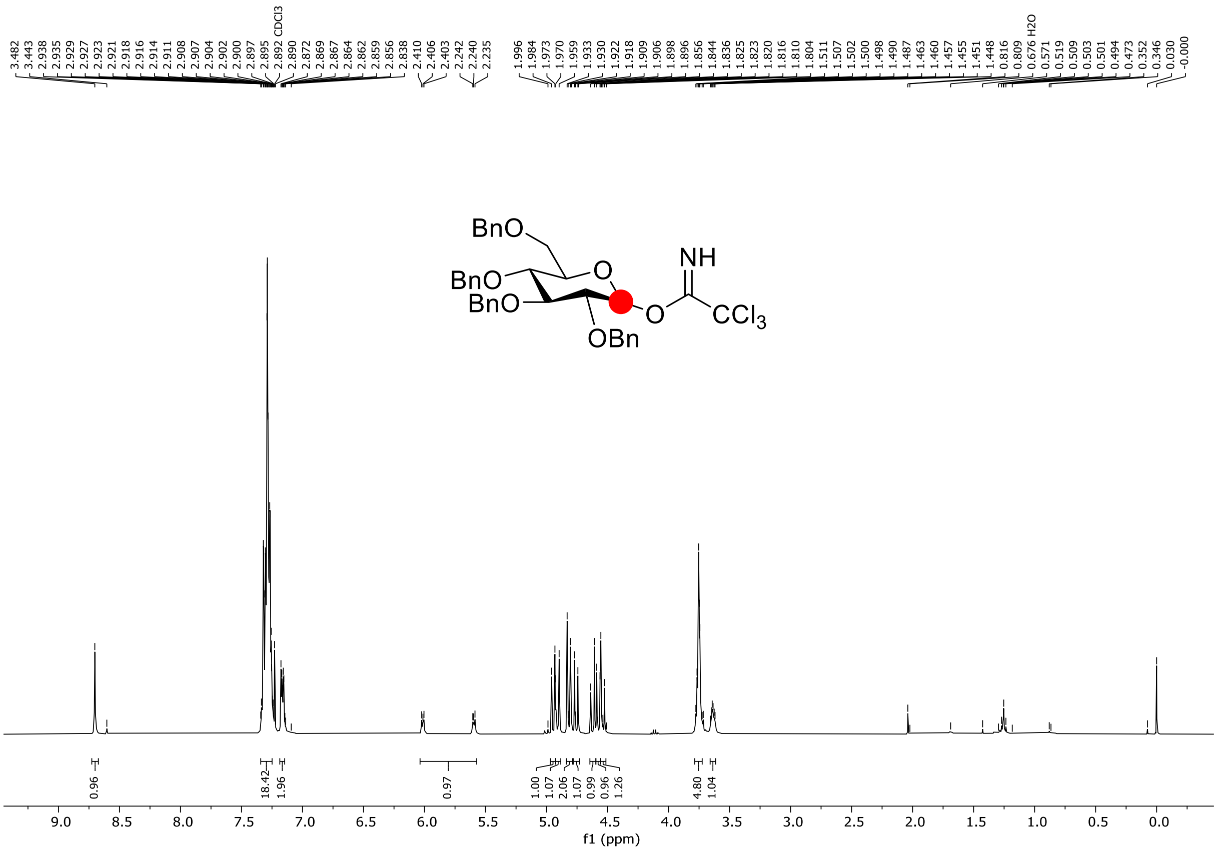

^{13}C NMR of anomeric ^{13}C 2,3,4,6-tetra-O-benzyl-β-D-glucopyranosyl trichloroacetimidate (6) (101 MHz, CDCl$_3$)

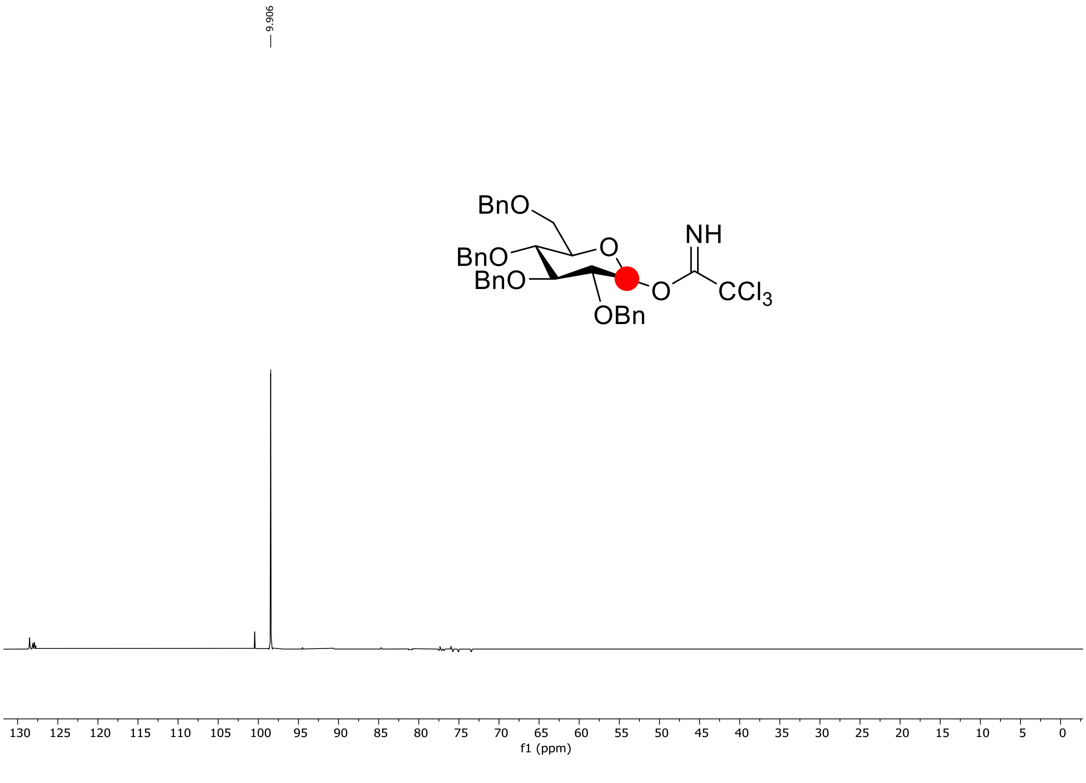

^{1}H NMR of ^{15}N 2,3,4,6-tetra-O-benzyl-β-D-glucopyranosyl trichloroacetimidate (6) (400 MHz, $CDCl_3$)

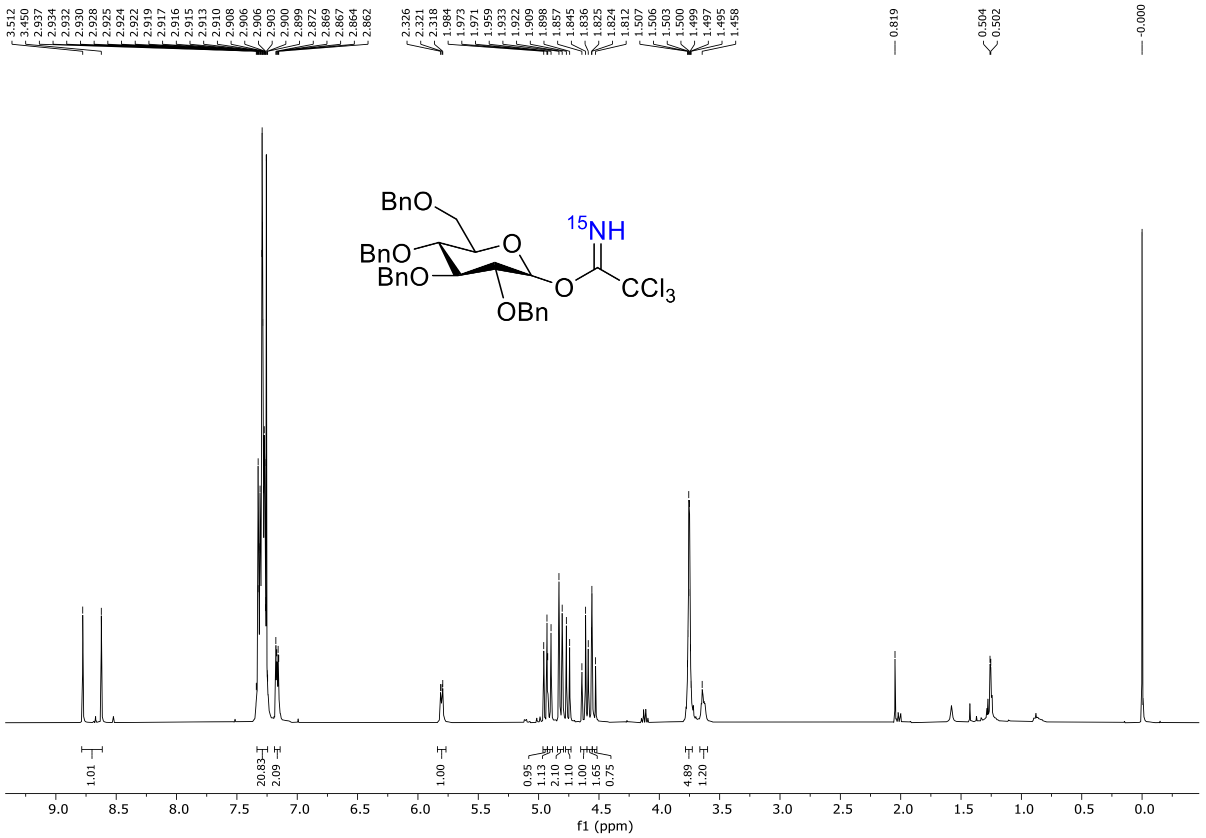

^{13}C NMR of ^{15}N 2,3,4,6-tetra-O-benzyl-β-D-glucopyranosyl trichloroacetimidate (6) (101 MHz, $CDCl_3$)

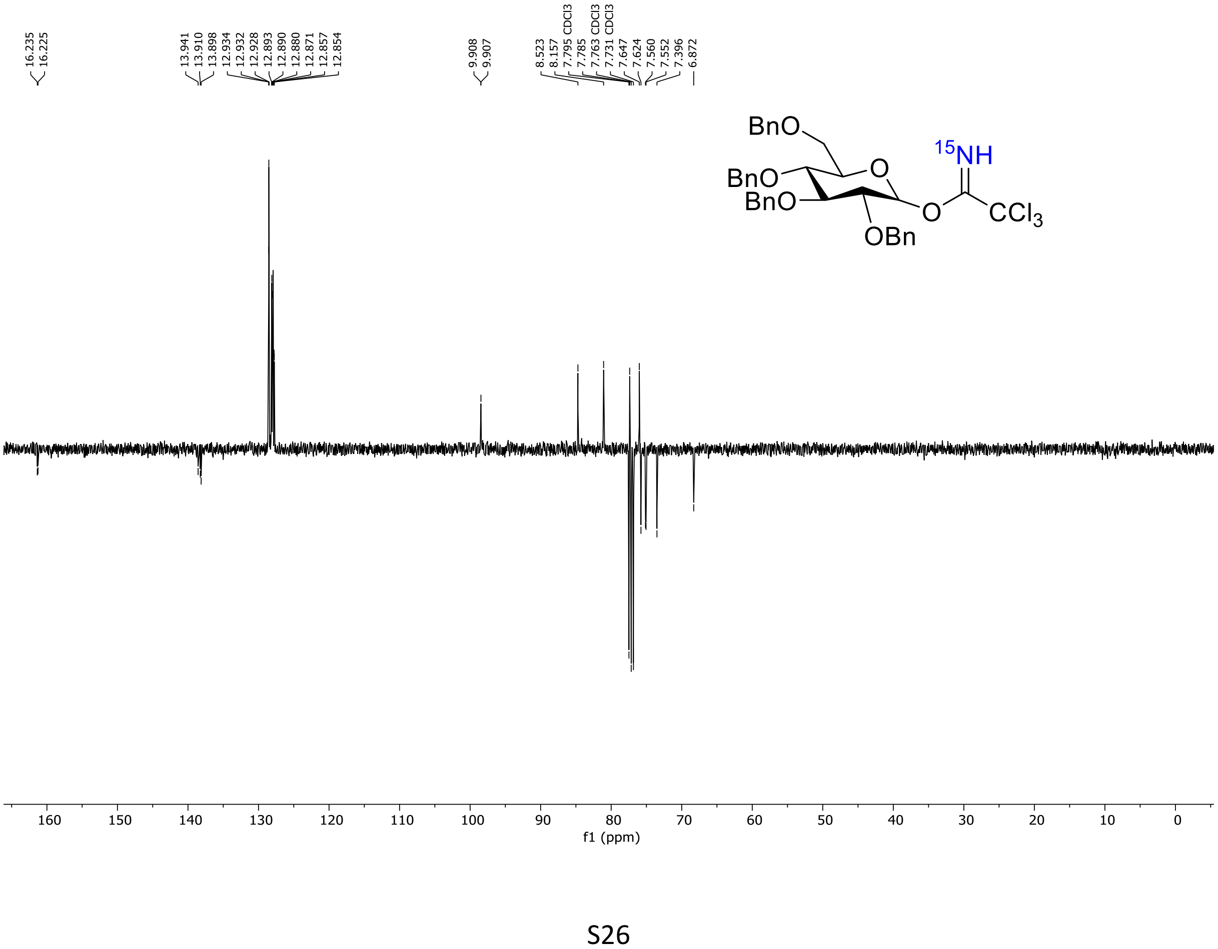

^{15}N NMR of ^{15}N 2,3,4,6-tetra-O-benzyl-β-D-glucopyranosyl trichloroacetimidate (6) (41 MHz, CDCl$_3$)

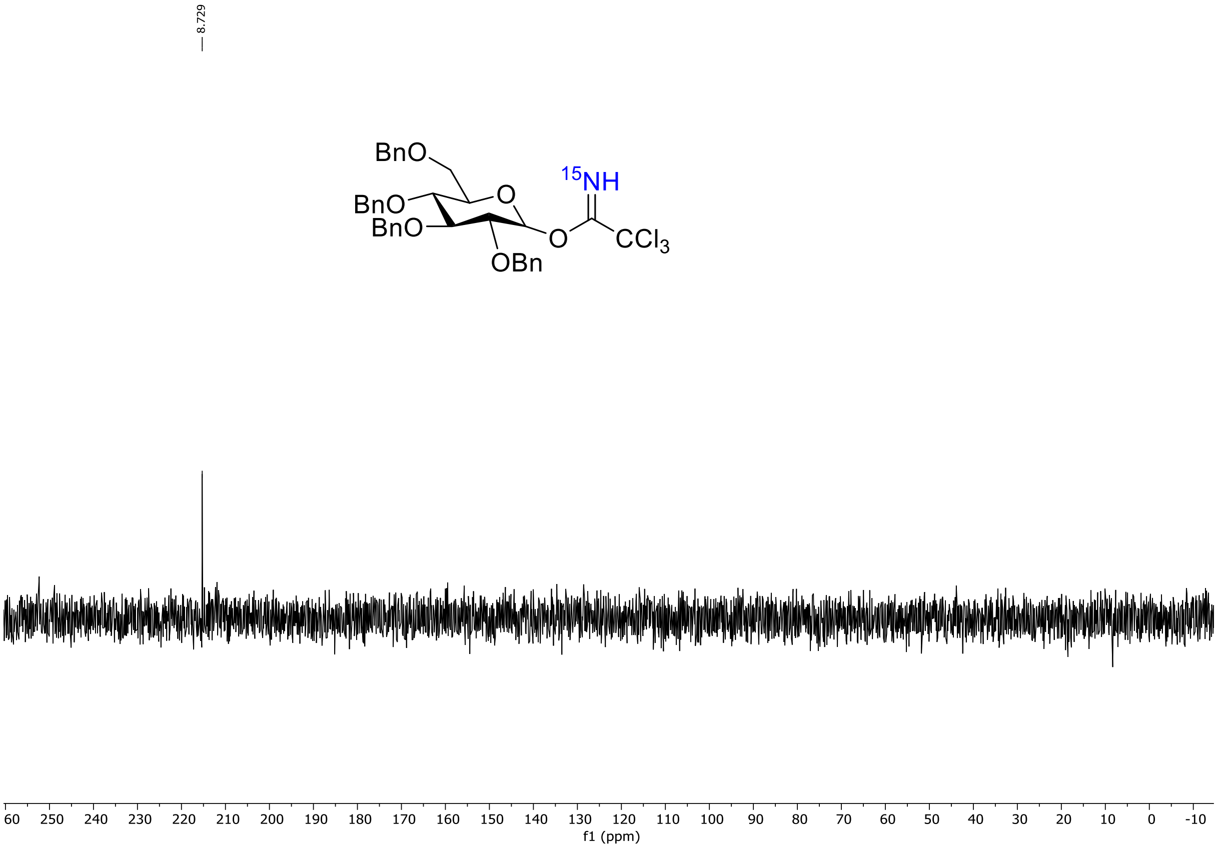

^{1}H NMR of phenyl 2,3,4,6-tetra-O-benzyl-1-thio-α-D-mannopyranoside (S3) (400 MHz, CDCl$_3$)

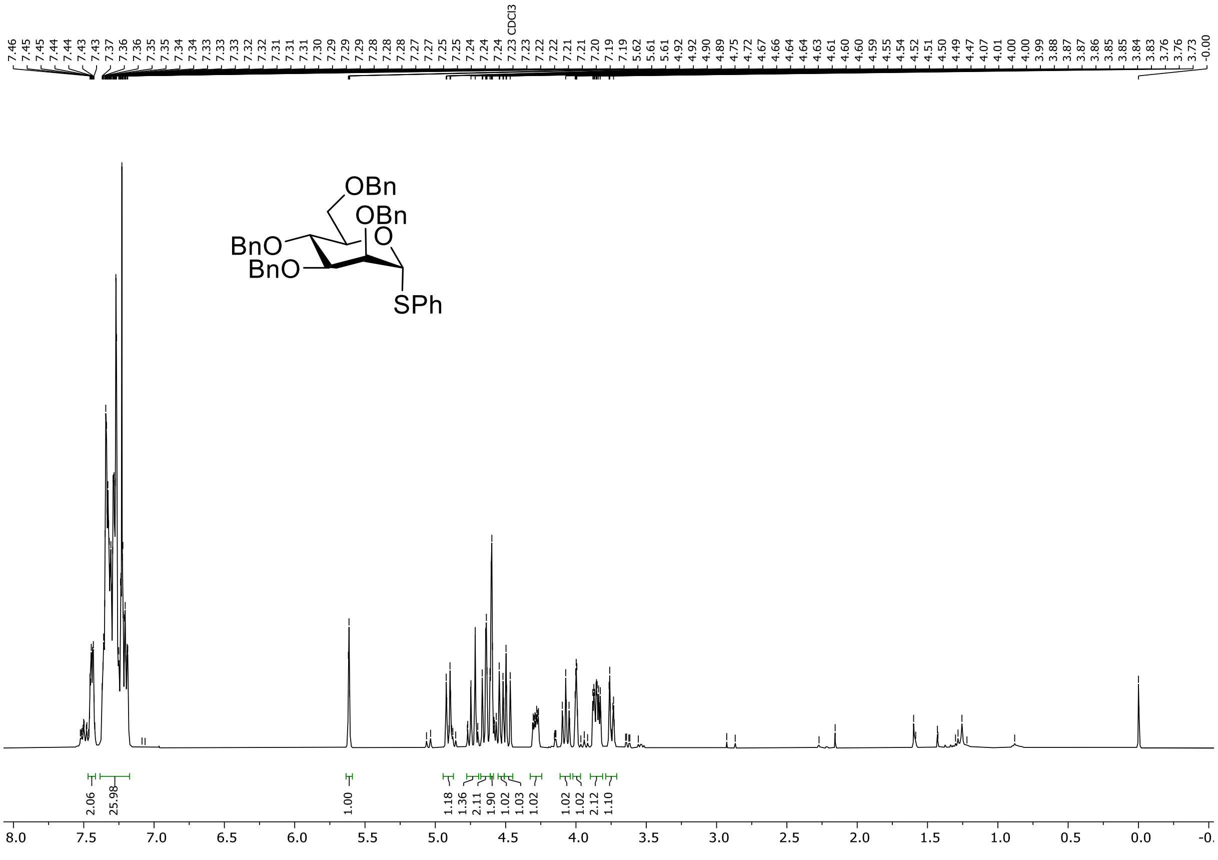

^{13}C NMR of phenyl 2,3,4,6-tetra-O-benzyl-1-thio-α-D-mannopyranoside (S3) (101 MHz, CDCl$_3$)

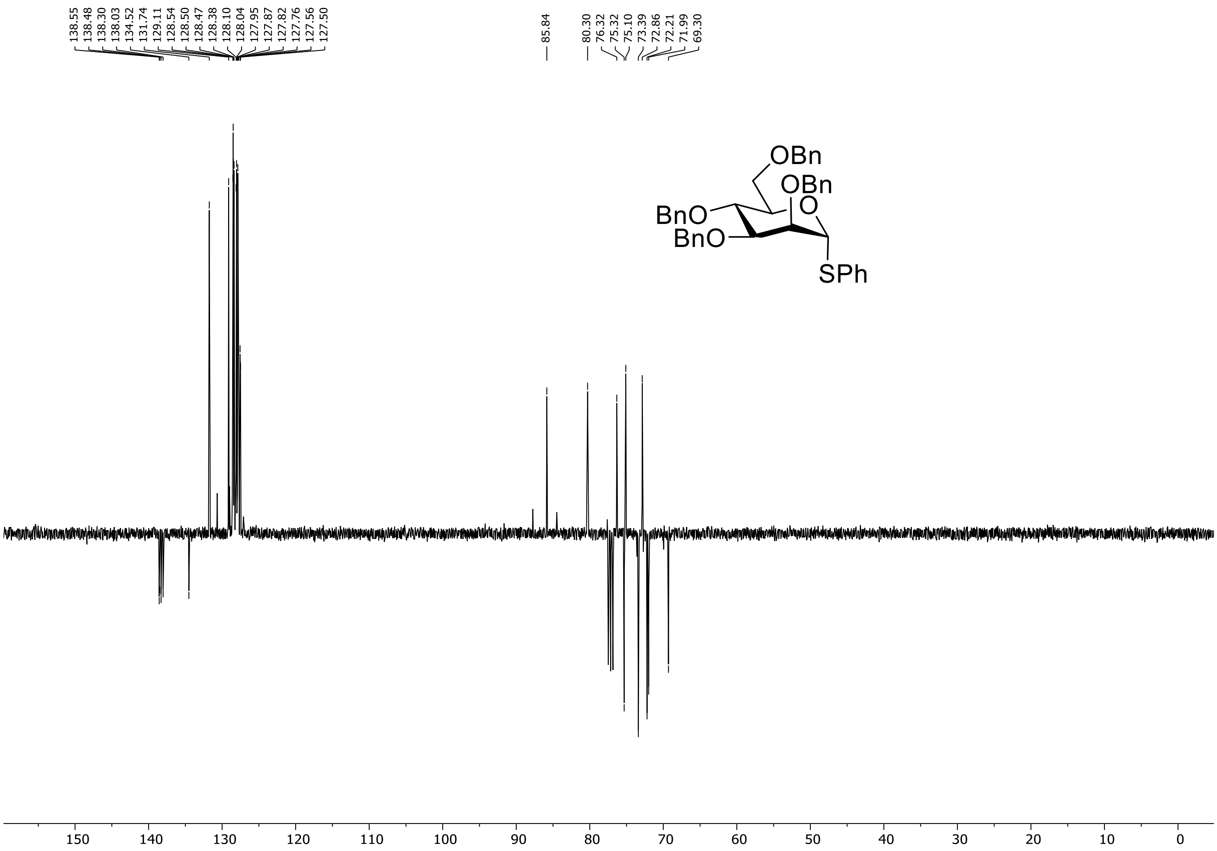

HH COSY of phenyl 2,3,4,6-tetra-O-benzyl-1-thio-α-D-mannopyranoside (S3) (CDCl$_3$)

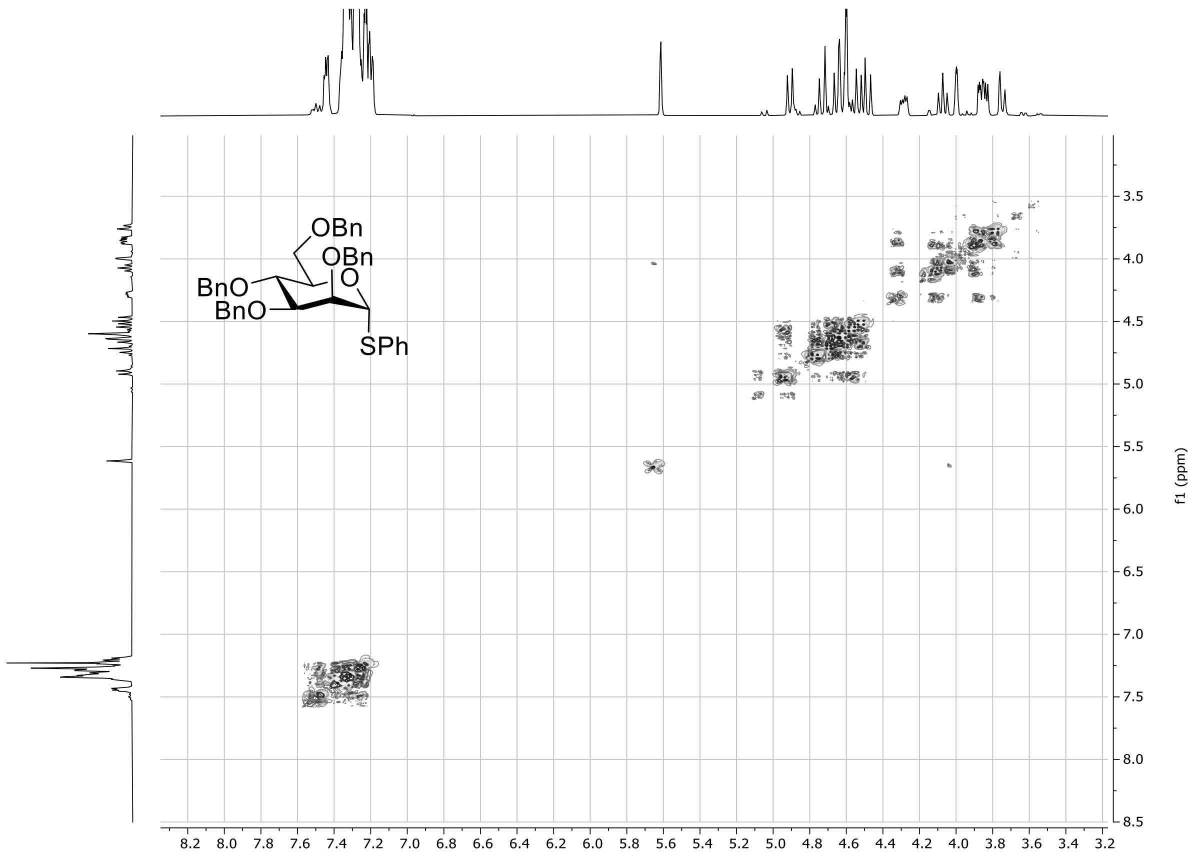

CH HSQC of phenyl 2,3,4,6-tetra-O-benzyl-1-thio-α-D-mannopyranoside (S3) (CDCl$_3$)

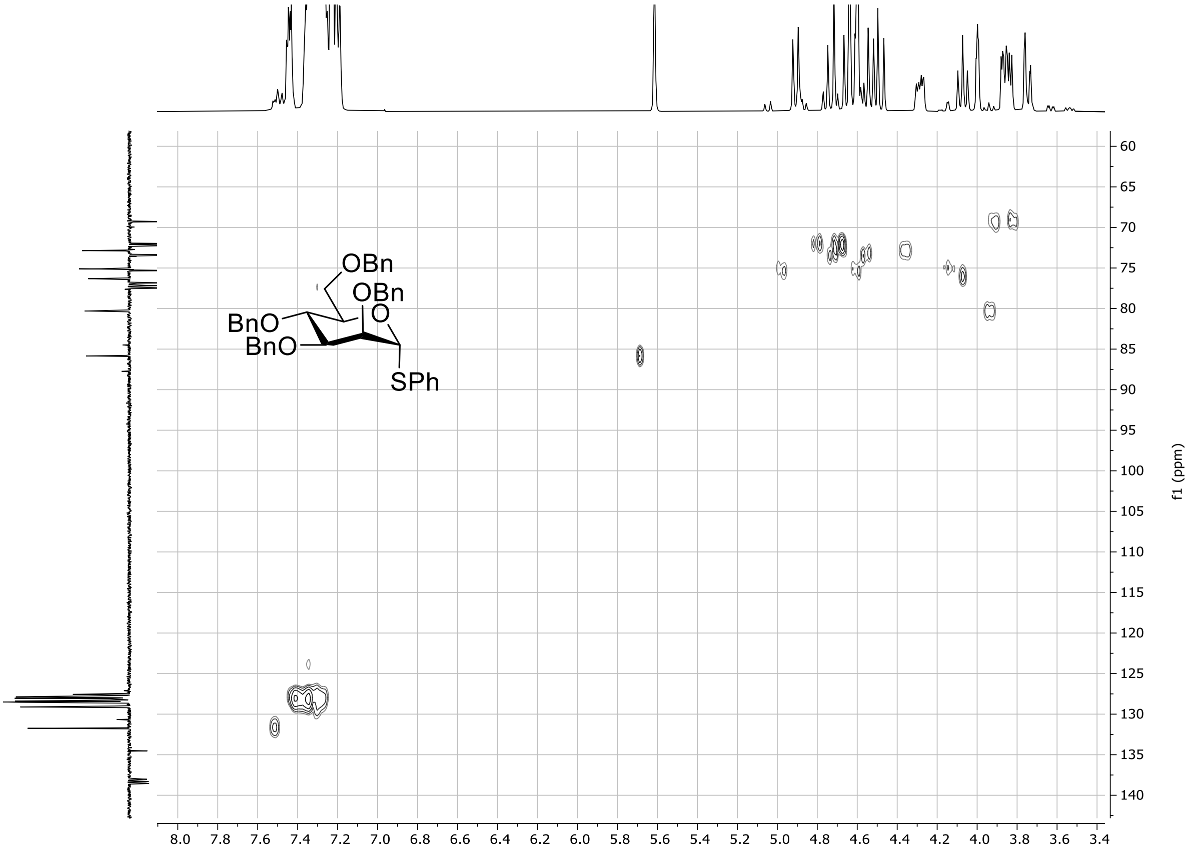

^{1}H NMR of 2,3,4,6-tetra-O-benzyl-D-mannospyranoside (S4) (400 MHz, CDCl$_3$)

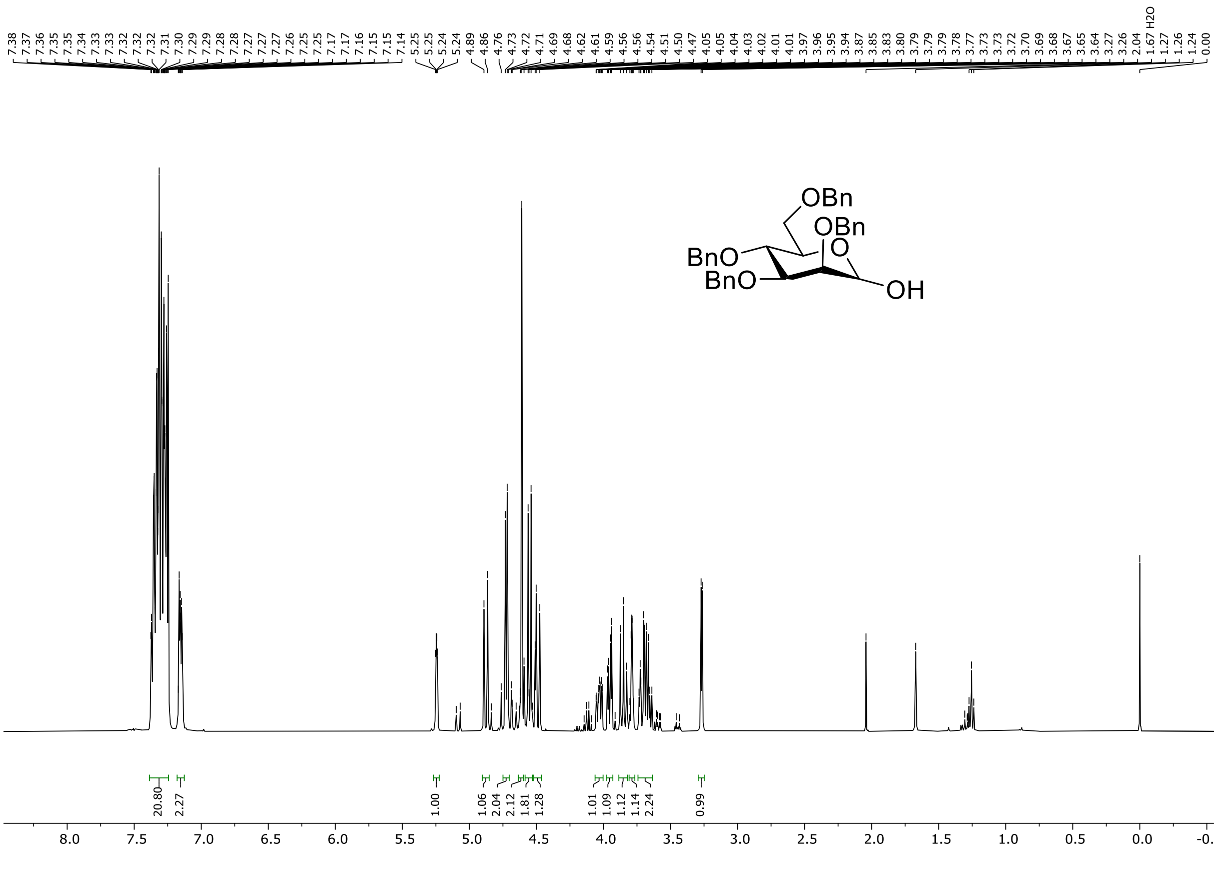

^{13}C NMR of 2,3,4,6-tetra-O-benzyl-D-mannospyranoside (S4) (101 MHz, CDCl$_3$)

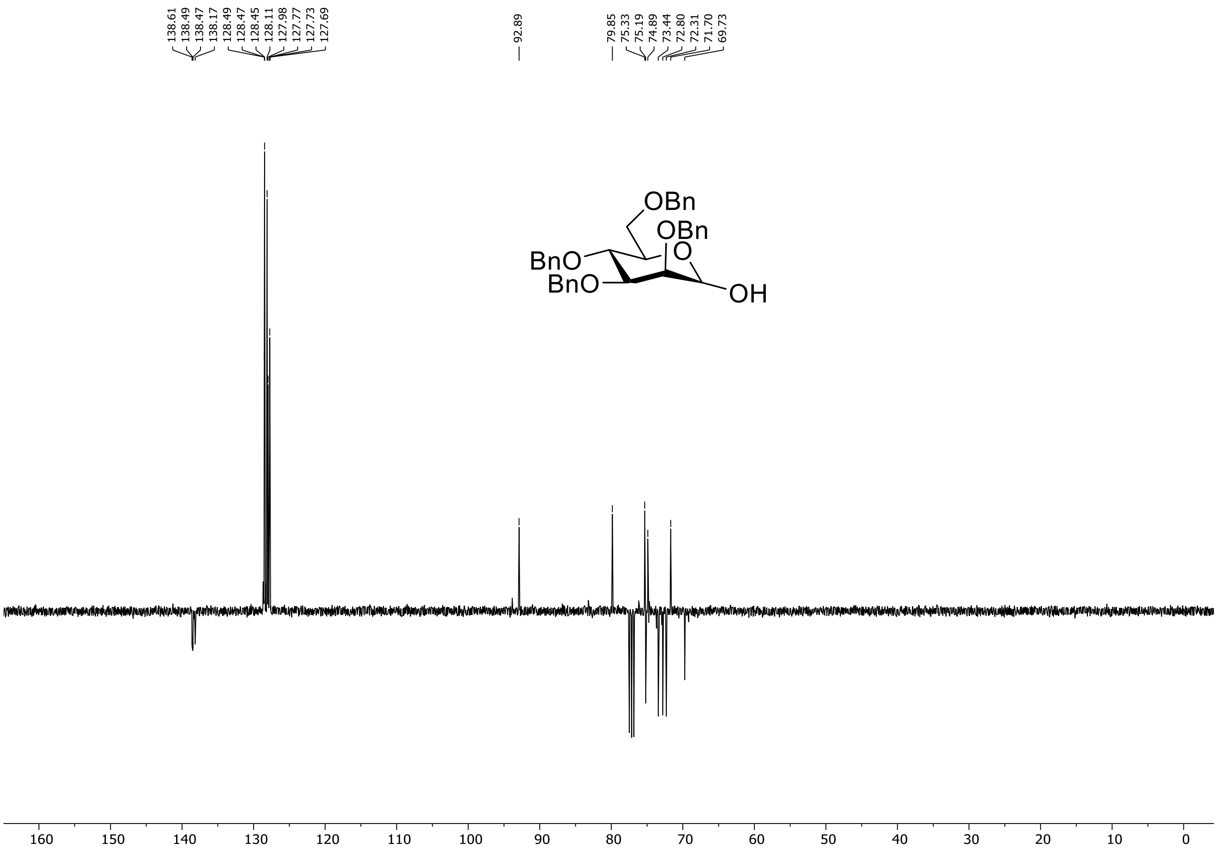

HH COSY of 2,3,4,6-tetra-O-benzyl-D-mannospyranoside (S4) (CDCl$_3$)

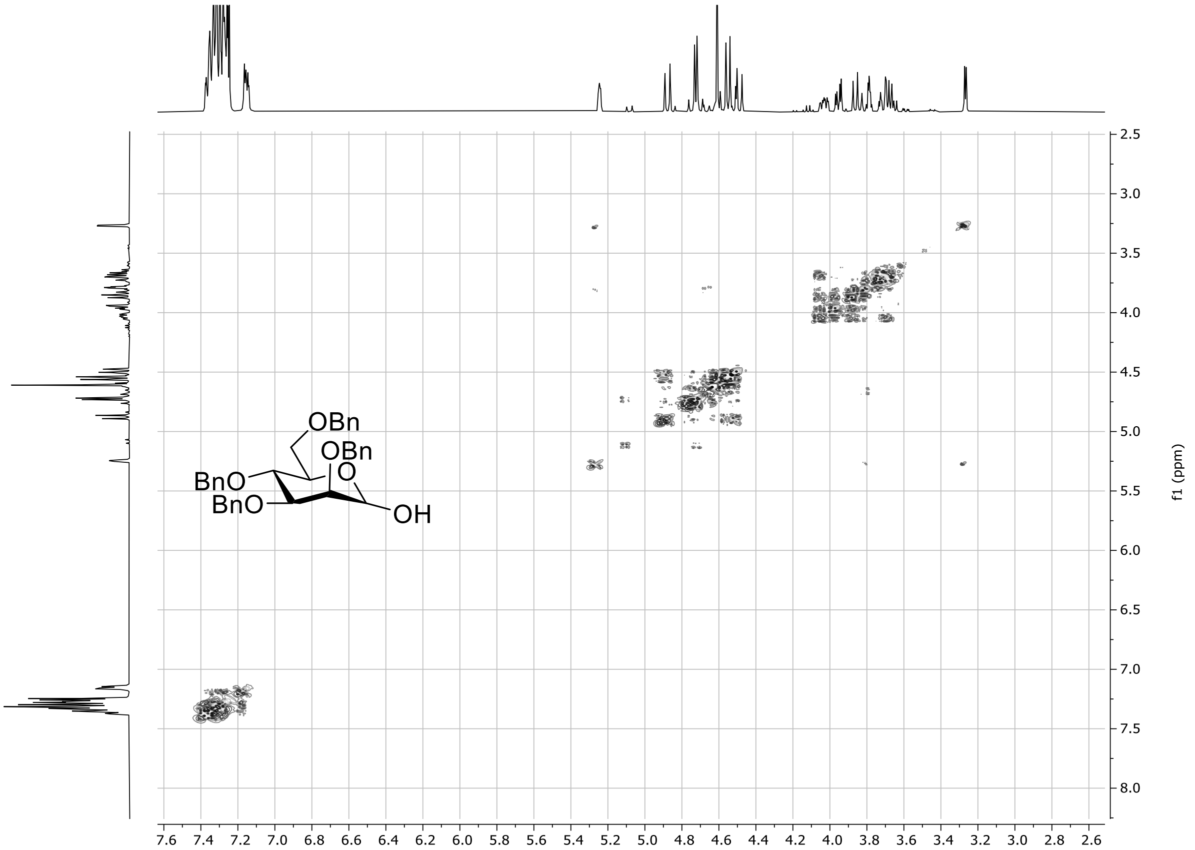

CH HSQC of 2,3,4,6-tetra-O-benzyl-D-mannospyranoside (S4) ($CDCl_3$)

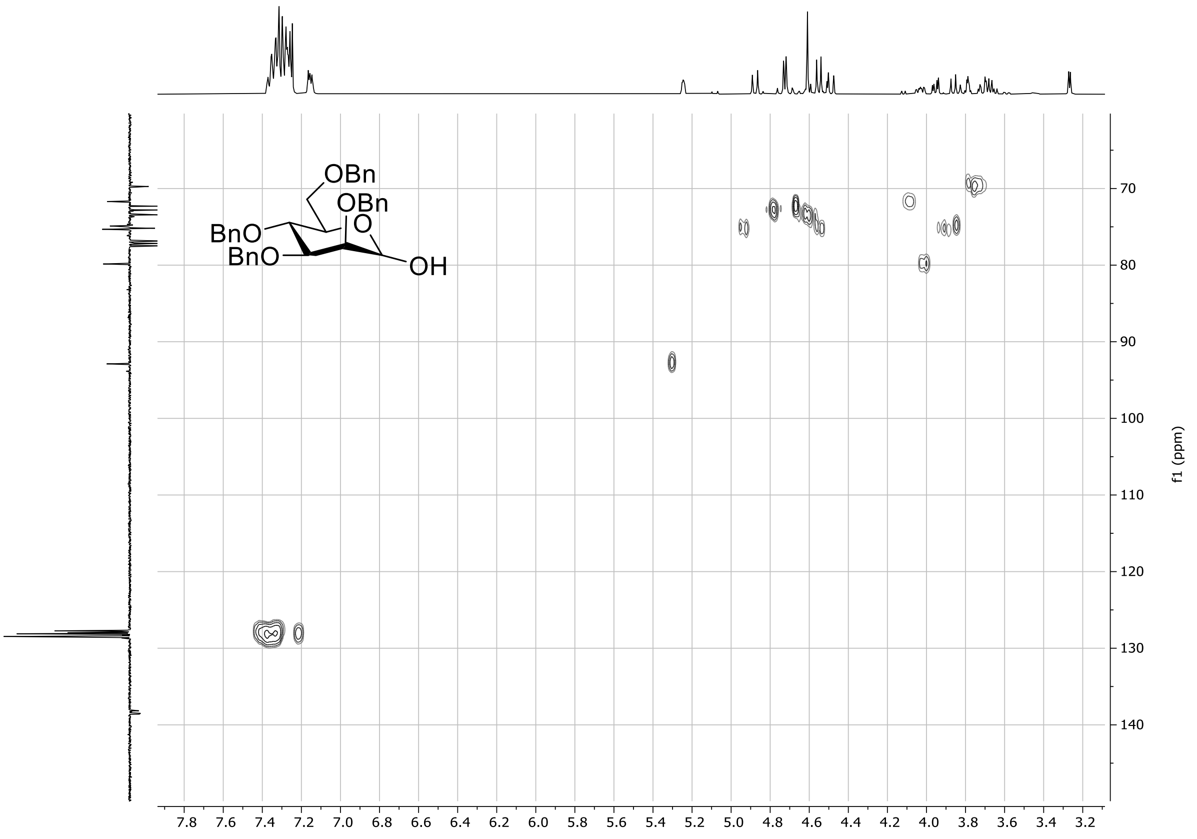

^{1}H NMR of 2,3,4,6-tetra-O-benzyl-α-D-mannopyranosyl trichloroacetimidate (7) (400 MHz, $CDCl_3$)

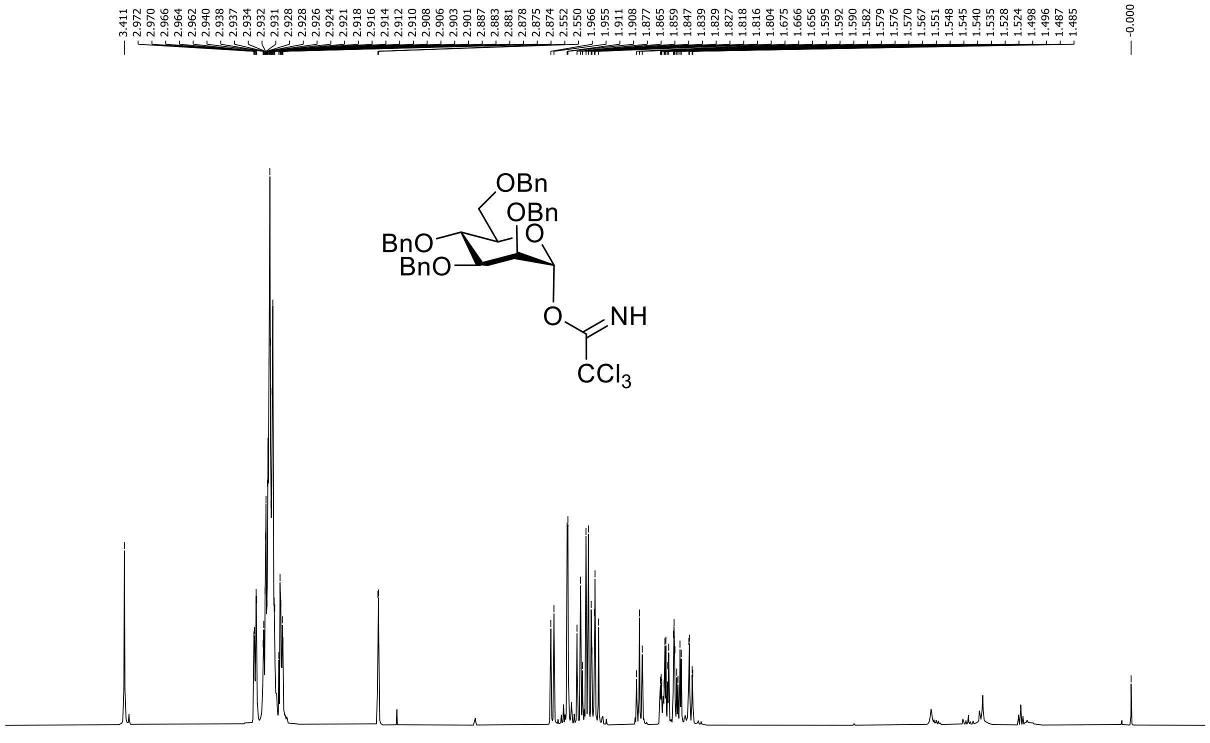

^{13}C NMR of 2,3,4,6-tetra-O-benzyl-α-D-mannopyranosyl trichloroacetimidate (7) (101 MHz, $CDCl_3$)

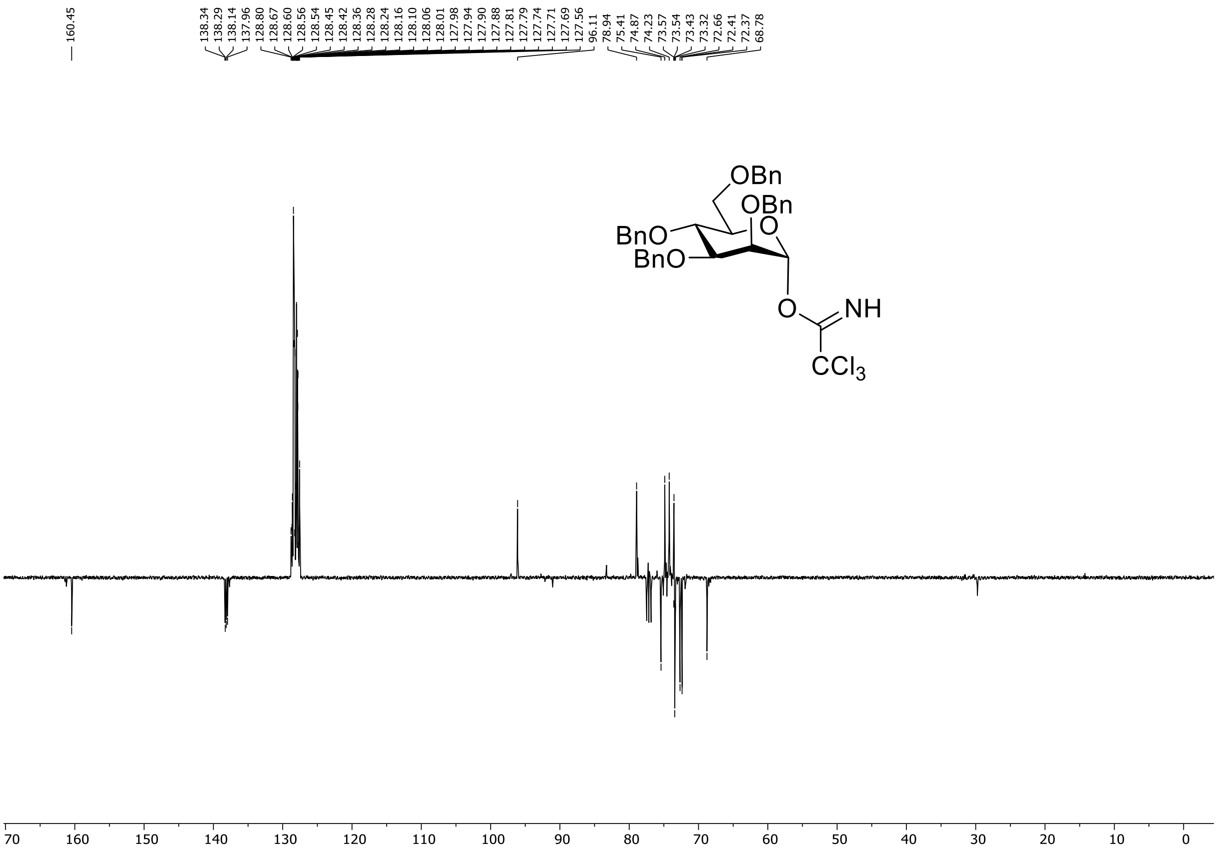

HH COSY of 2,3,4,6-tetra-O-benzyl-α-D-mannopyranosyl trichloroacetimidate (7) ($CDCl_3$)

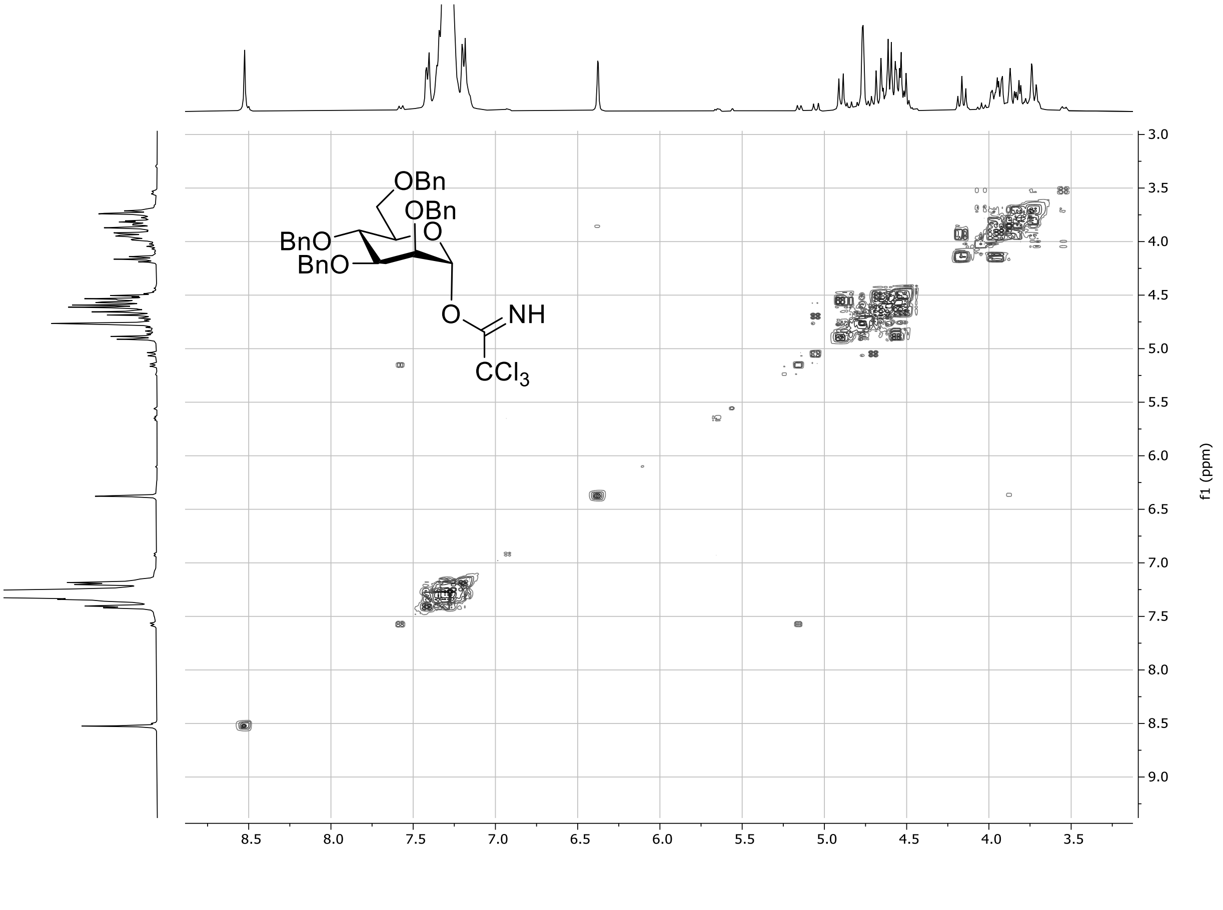

CH HSQC of 2,3,4,6-tetra-O-benzyl-α-D-mannopyranosyl trichloroacetimidate (7) ($CDCl_3$)

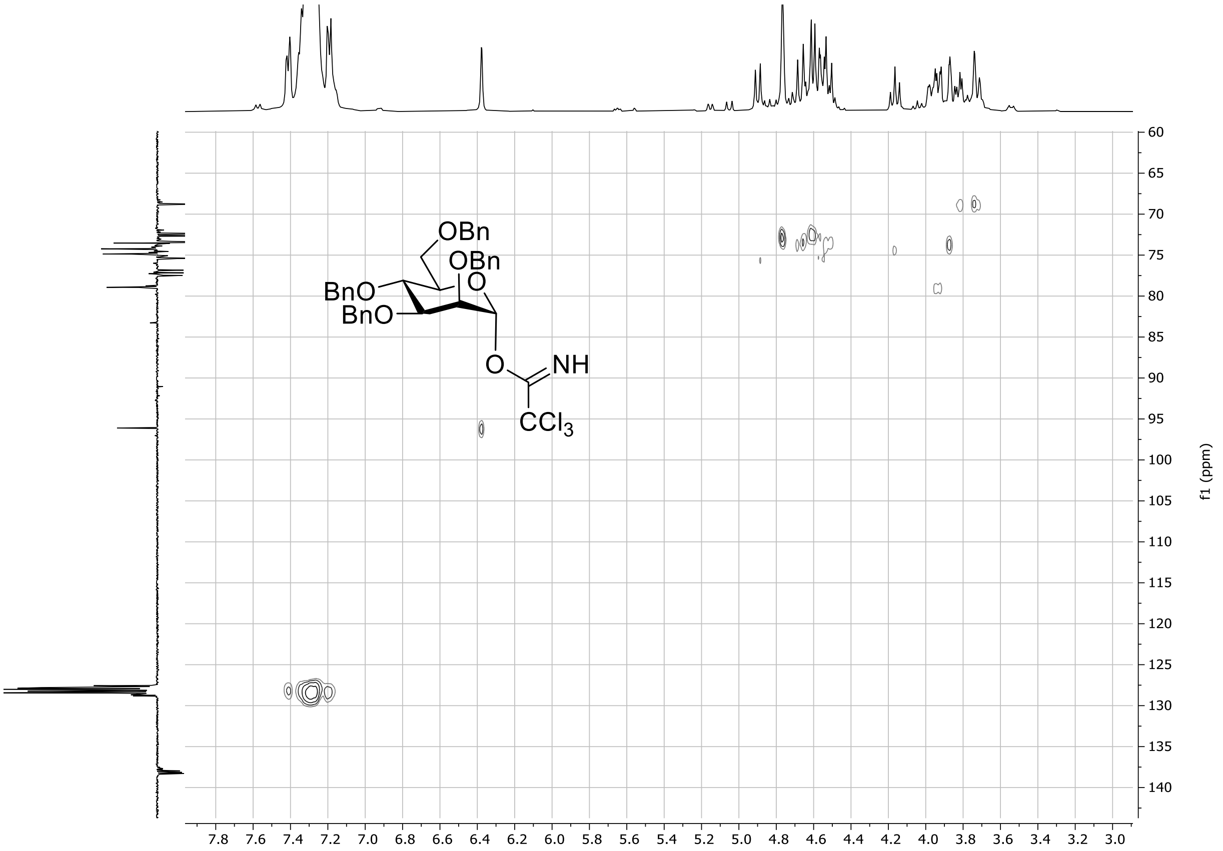

^{1}H NMR of anomeric ^{13}C 2,3,4,6-tetra-O-benzyl-α-D-mannopyranosyl trichloroacetimidate (7) (400 MHz, $CDCl_3$)

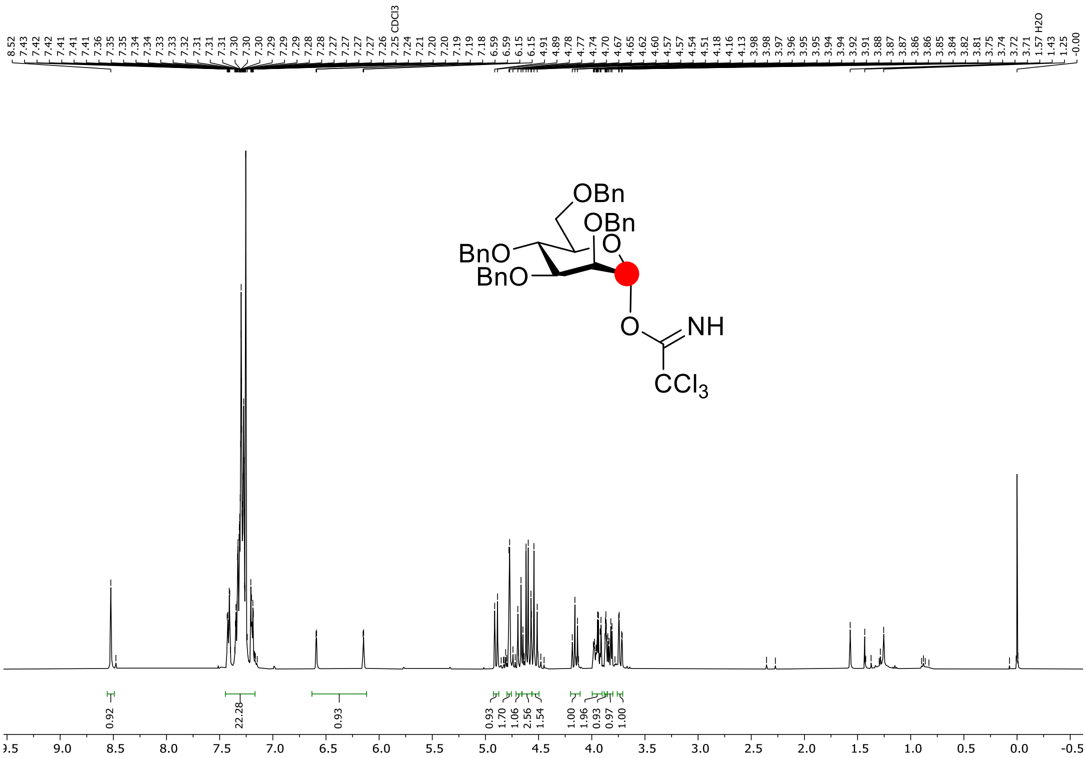

^{13}C NMR of anomeric ^{13}C 2,3,4,6-tetra-O-benzyl-α-D-mannopyranosyl trichloroacetimidate (7) (101 MHz, $CDCl_3$)

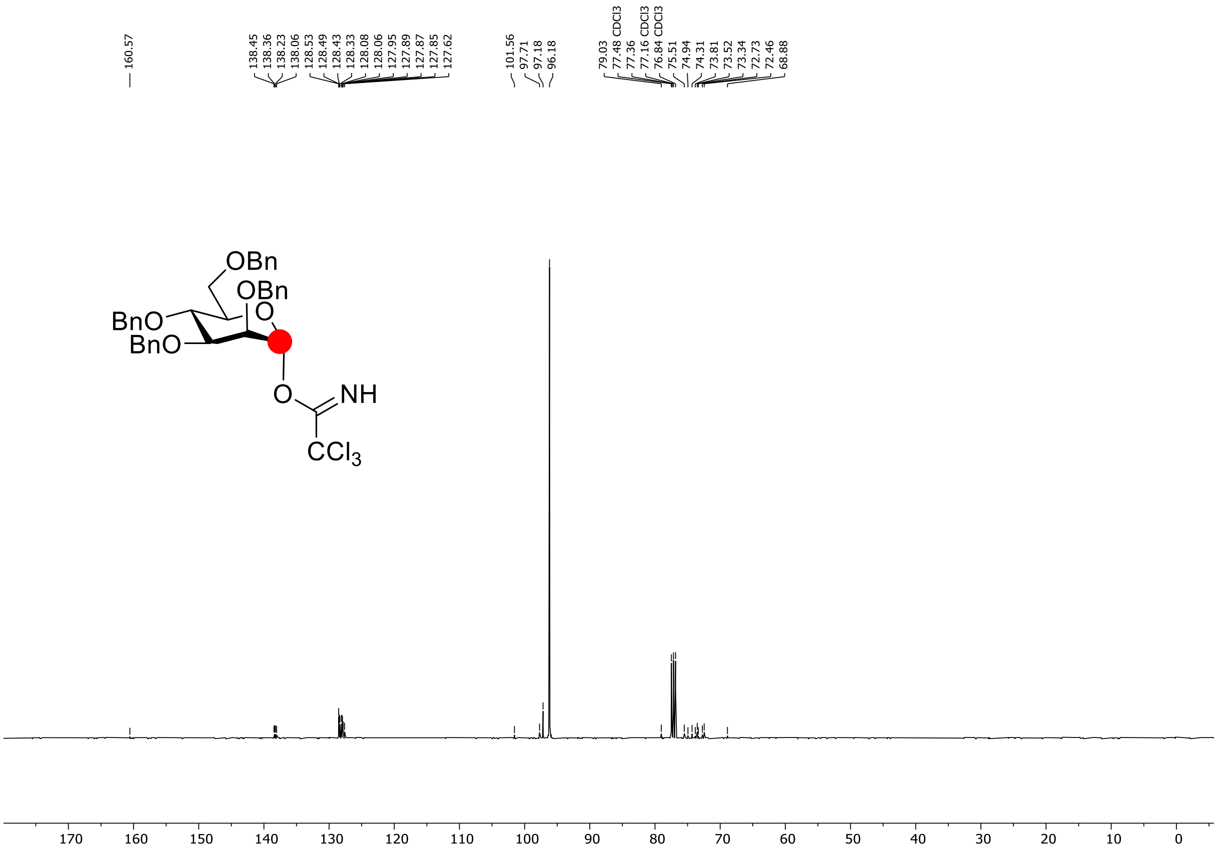

^{1}H NMR of ^{15}N 2,3,4,6-tetra-O-benzyl-α-D-mannopyranosyl trichloroacetimidate (7) (400 MHz, $CDCl_3$)

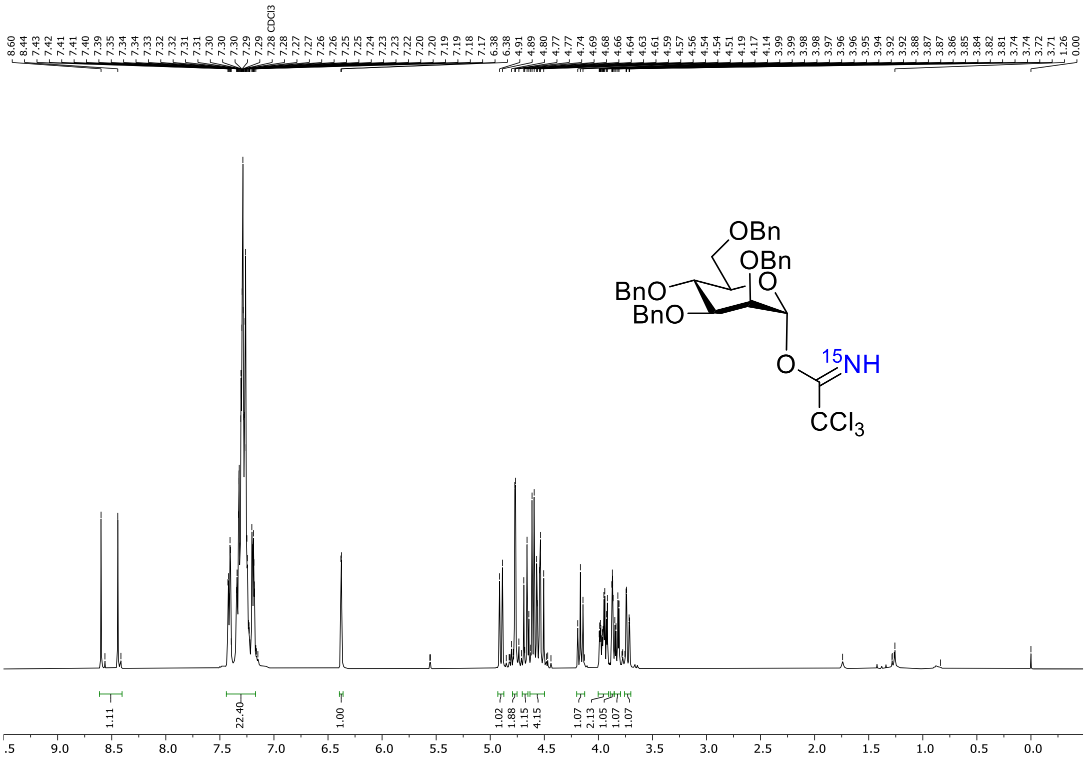

^{13}C NMR of ^{15}N 2,3,4,6-tetra-O-benzyl-α-D-mannopyranosyl trichloroacetimidate (7) (101 MHz, CDCl$_3$)

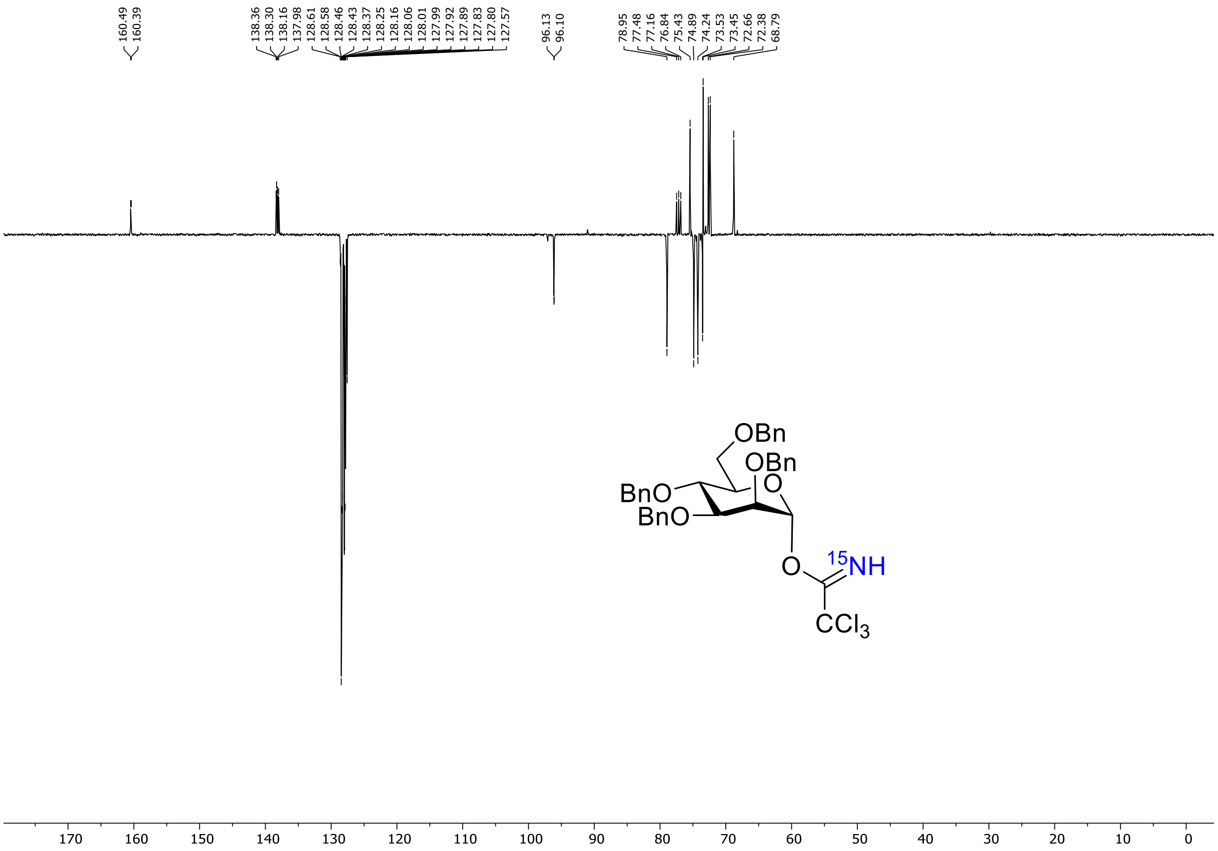

^{15}N NMR of ^{15}N 2,3,4,6-tetra-O-benzyl-α-D-mannopyranosyl trichloroacetimidate (7) (41 MHz, CDCl$_3$)

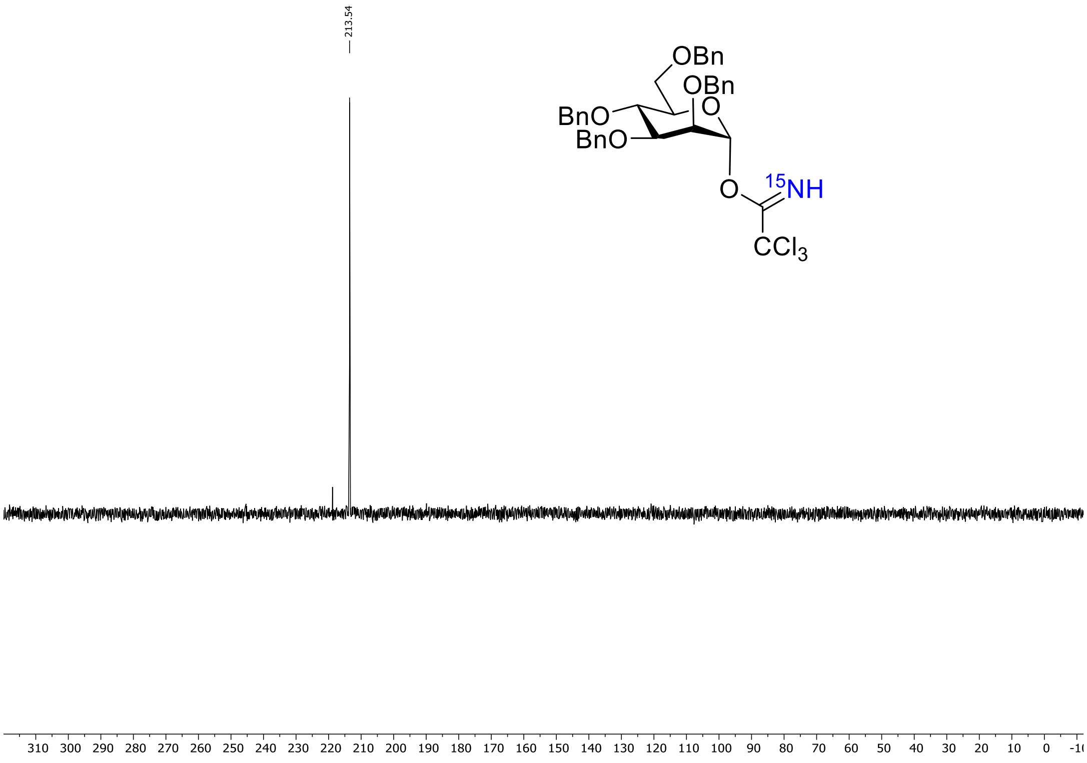

^{1}H NMR of N-trichloroacetyl-2,3,4,6-tetra-O-benzyl-α-D-glucopyranosylamide (8) (400 MHz, Acetone-d6)

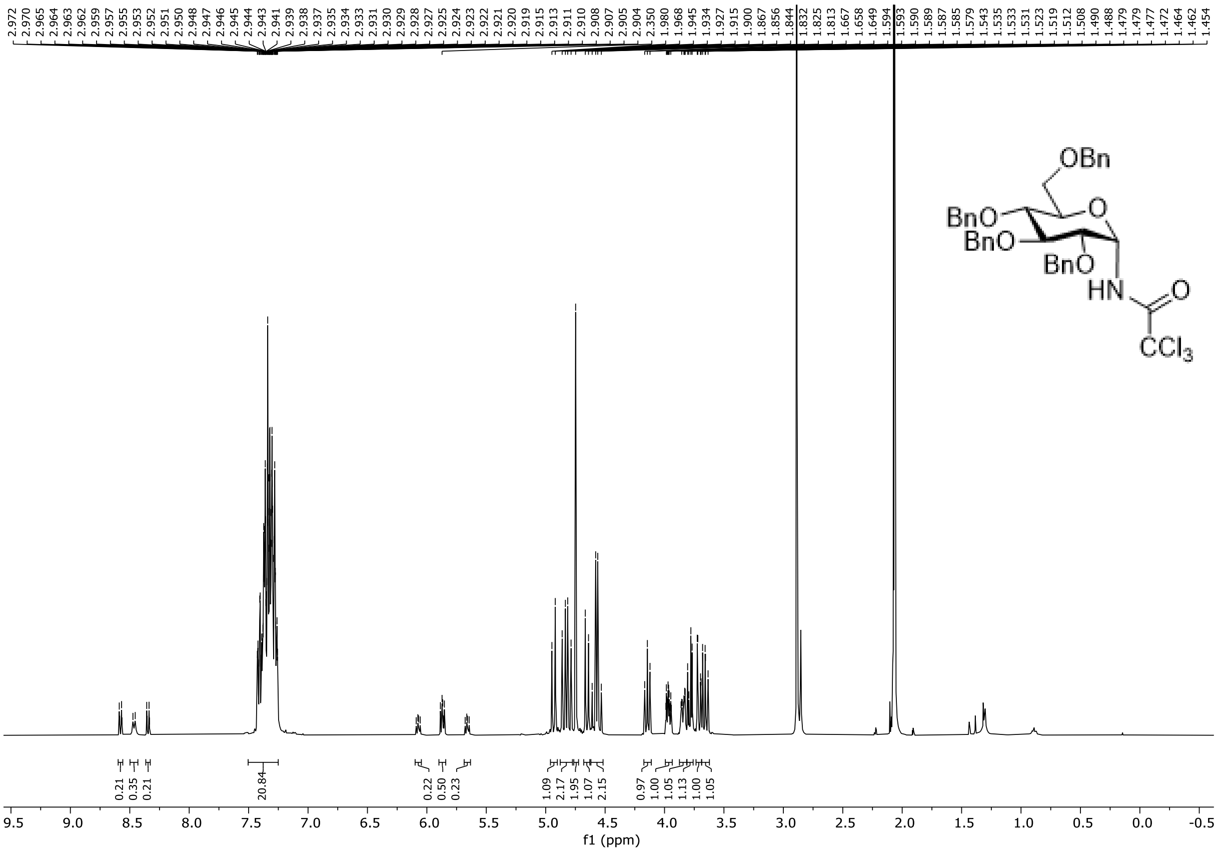

^{13}C NMR of N-trichloroacetyl-2,3,4,6-tetra-O-benzyl-α-D-glucopyranosylamide (8) (101 MHz, Acetone-d6)

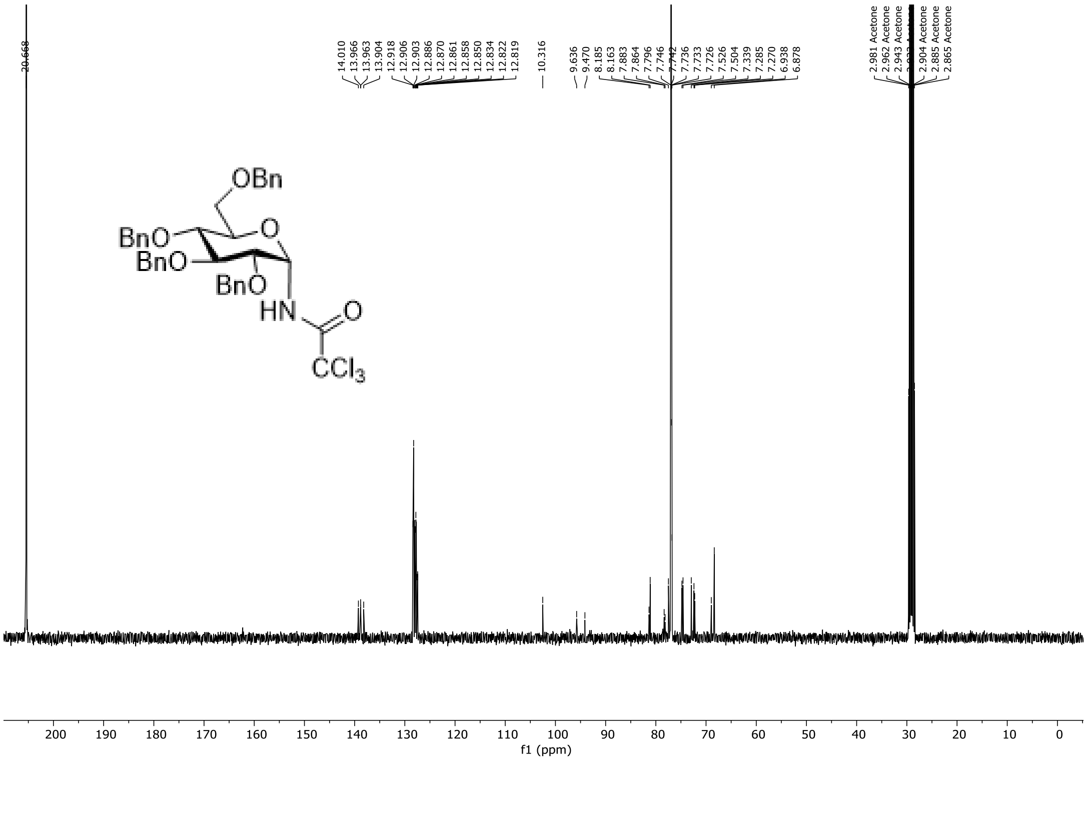

^{13}C NMR (Inverse Gated) of N-trichloroacetyl-2,3,4,6-tetra-O-benzyl-α-D-glucopyranosylamide (8) (101 MHz, Toluene-d8)

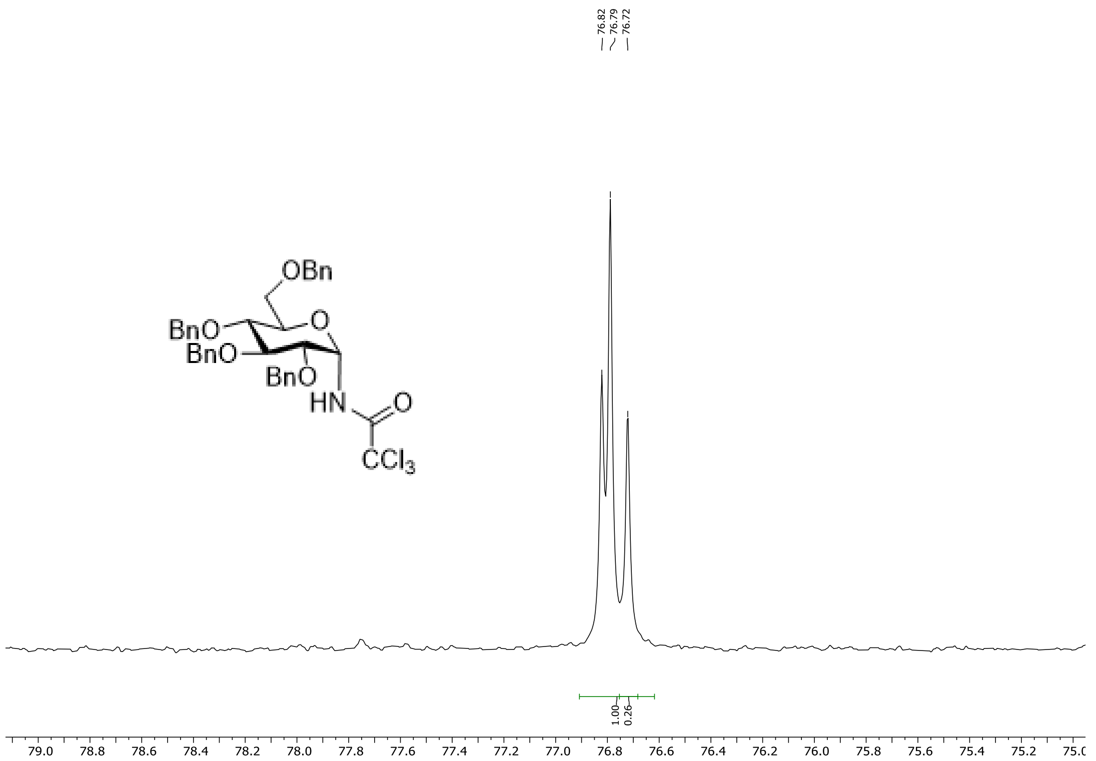

^{15}N NMR of N-trichloroacetyl-2,3,4,6-tetra-O-benzyl-α-D-glucopyranosylamide (8) (51 MHz, Acetone-d6)

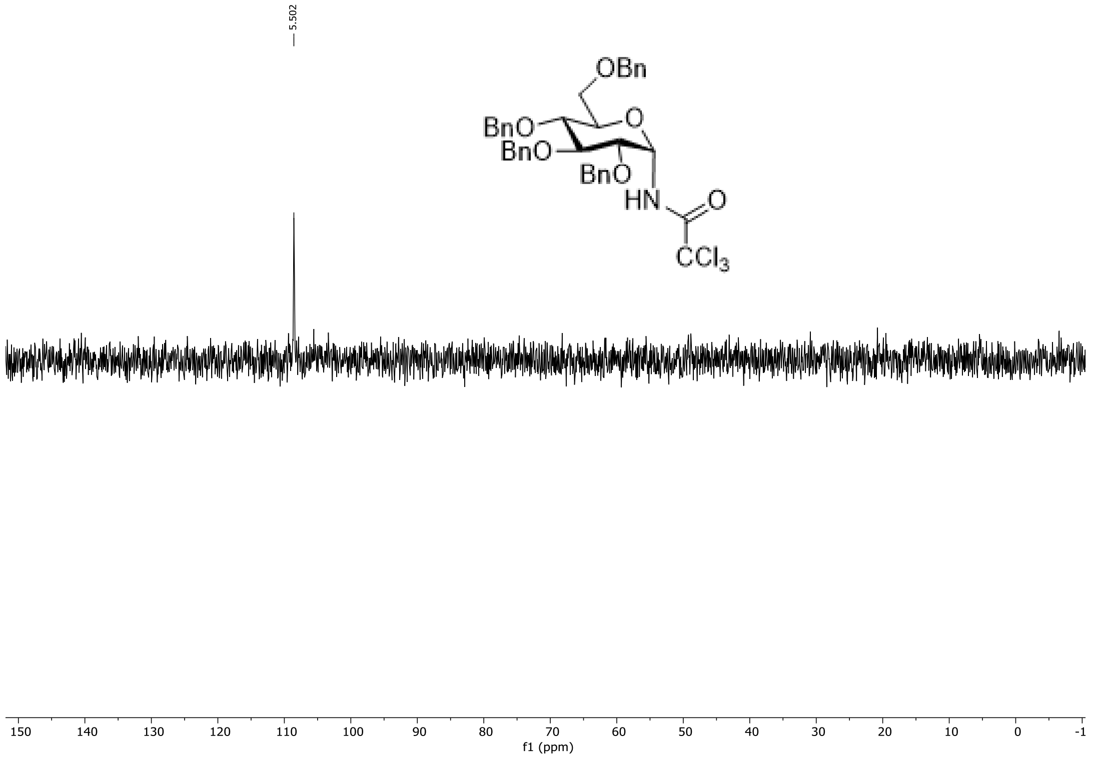

HH-COSY of N-trichloroacetyl-2,3,4,6-tetra-O-benzyl-α-D-glucopyranosylamide (8) (Acetone-d6)

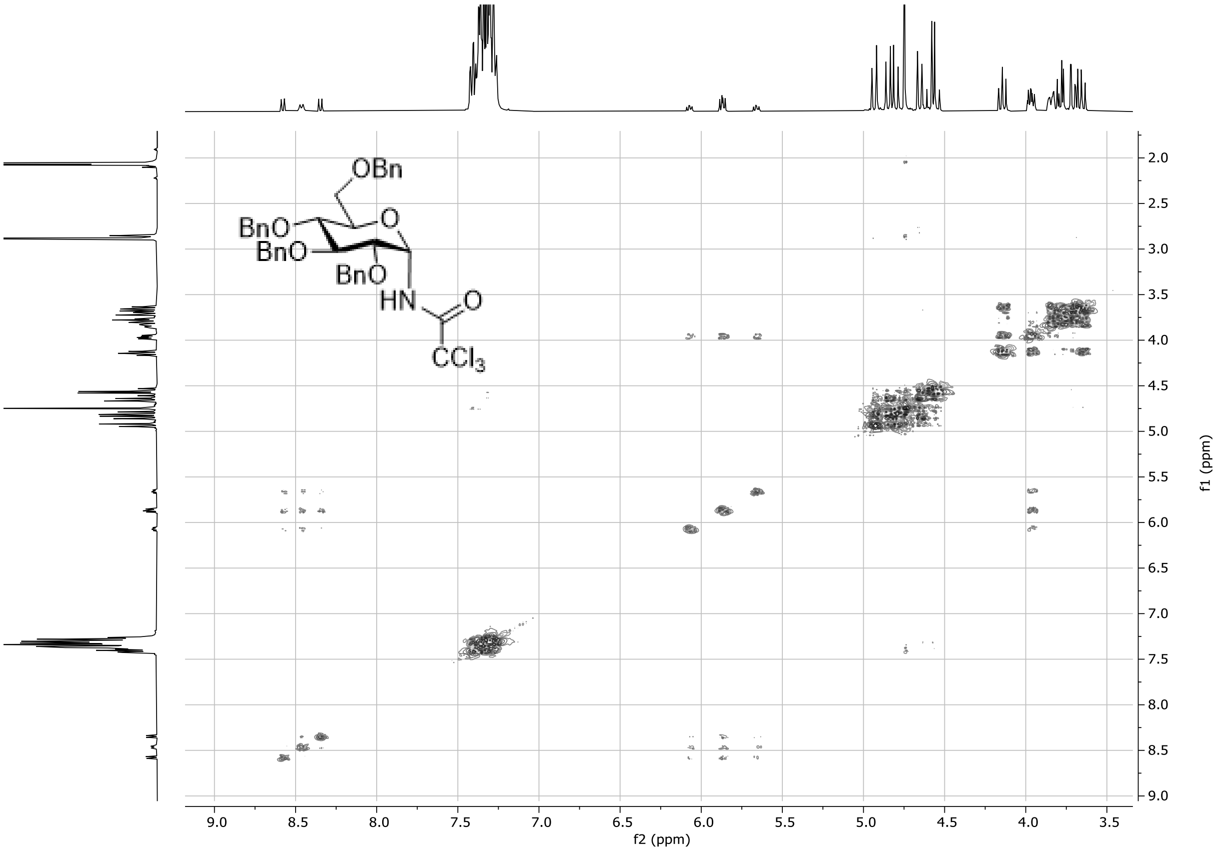

CH HSQC of N-trichloroacetyl-2,3,4,6-tetra-O-benzyl-α-D-glucopyranosylamide (8) (Acetone-d6)

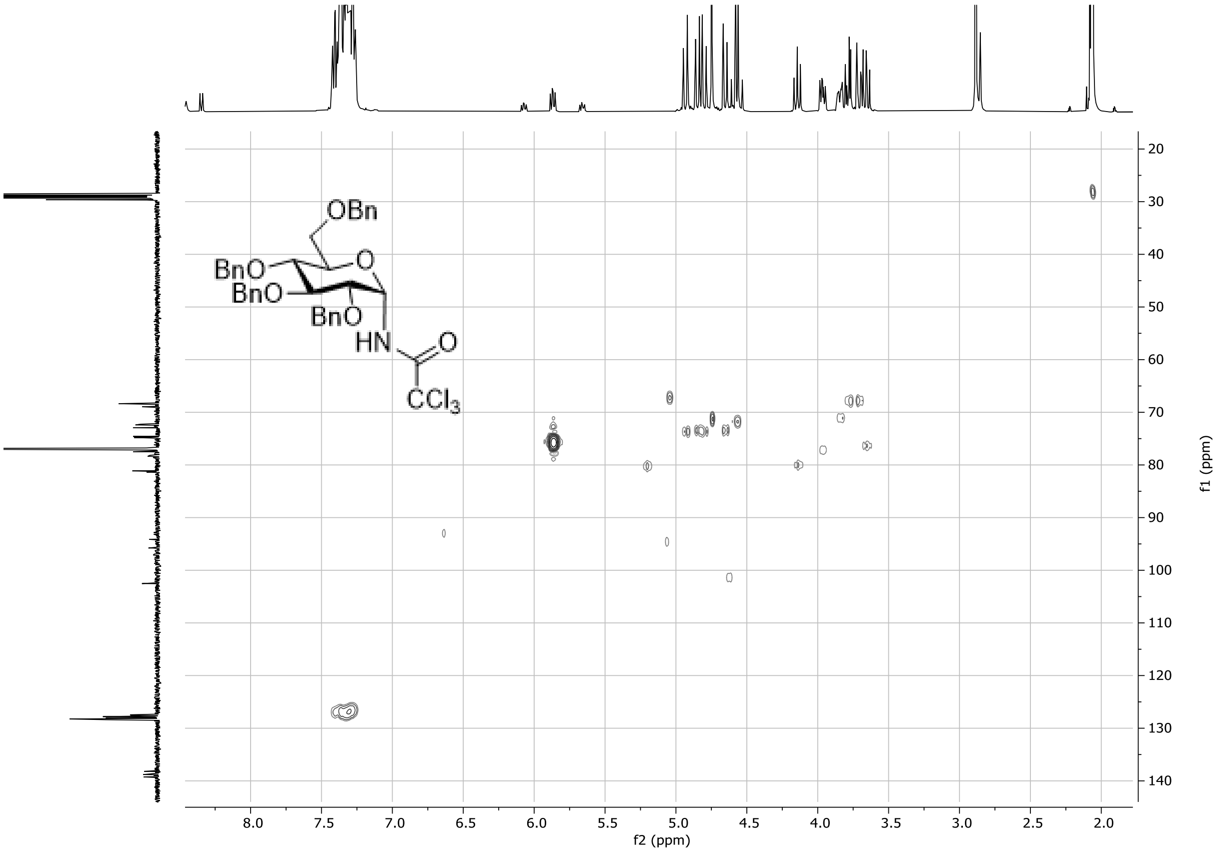

NH HSQC of N-trichloroacetyl-2,3,4,6-tetra-O-benzyl-α-D-glucopyranosylamide (8) (Acetone-d6)

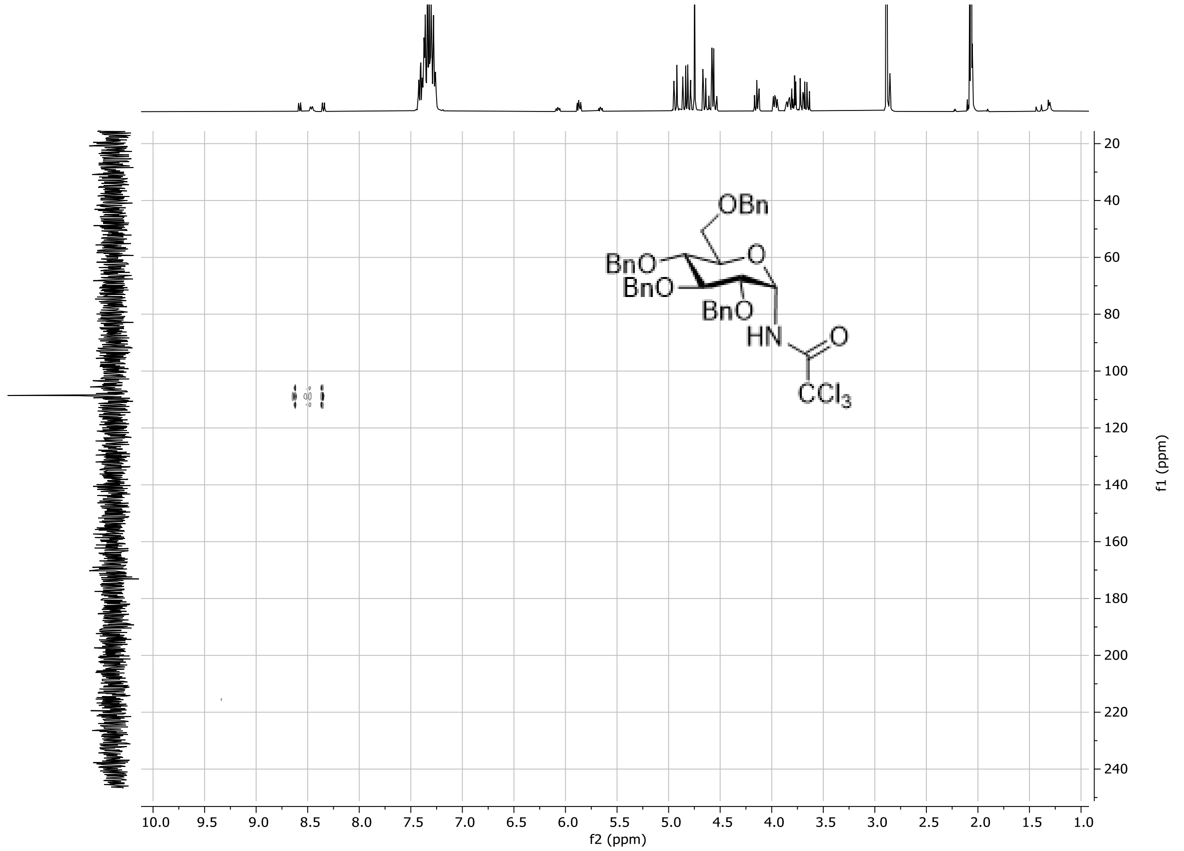

NH HMBC of N-trichloroacetyl-2,3,4,6-tetra-O-benzyl-α-D-glucopyranosylamide (8) (Acetone-d6)

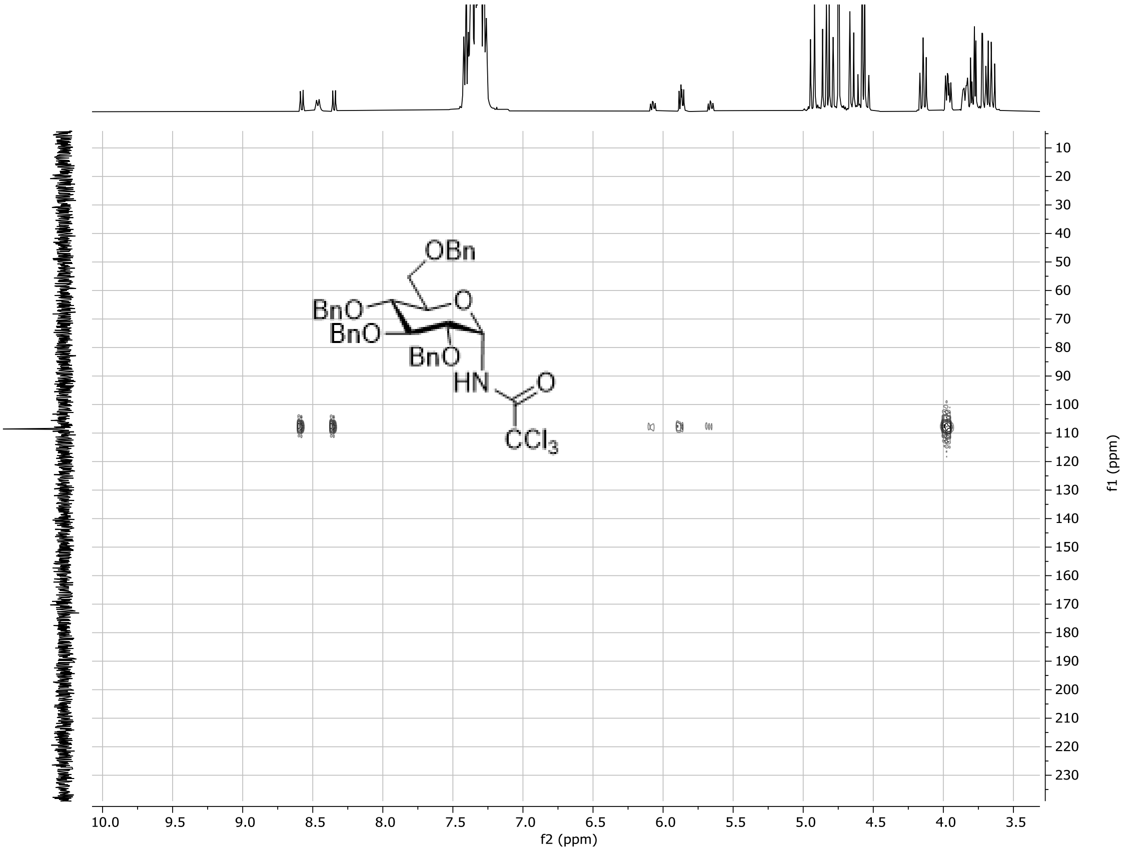

^{1}H NMR (^{13}C decoupled) of N-trichloroacetyl-2,3,4,6-tetra-O-benzyl-α-D-glucopyranosylamide (8) (Acetone-d6)

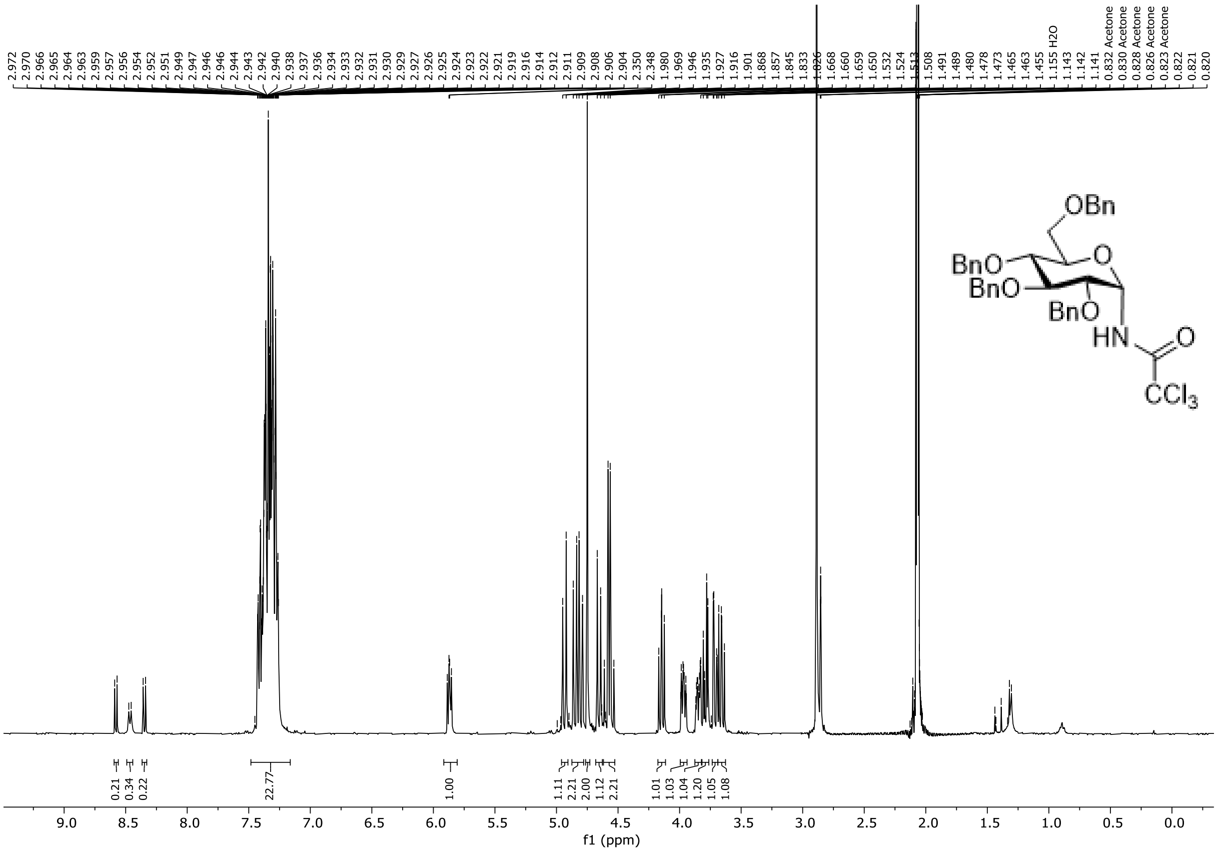

^{1}H NMR (^{15}N decoupled) of N-trichloroacetyl-2,3,4,6-tetra-O-benzyl-α-D-glucopyranosylamide (8) (Acetone-d6)

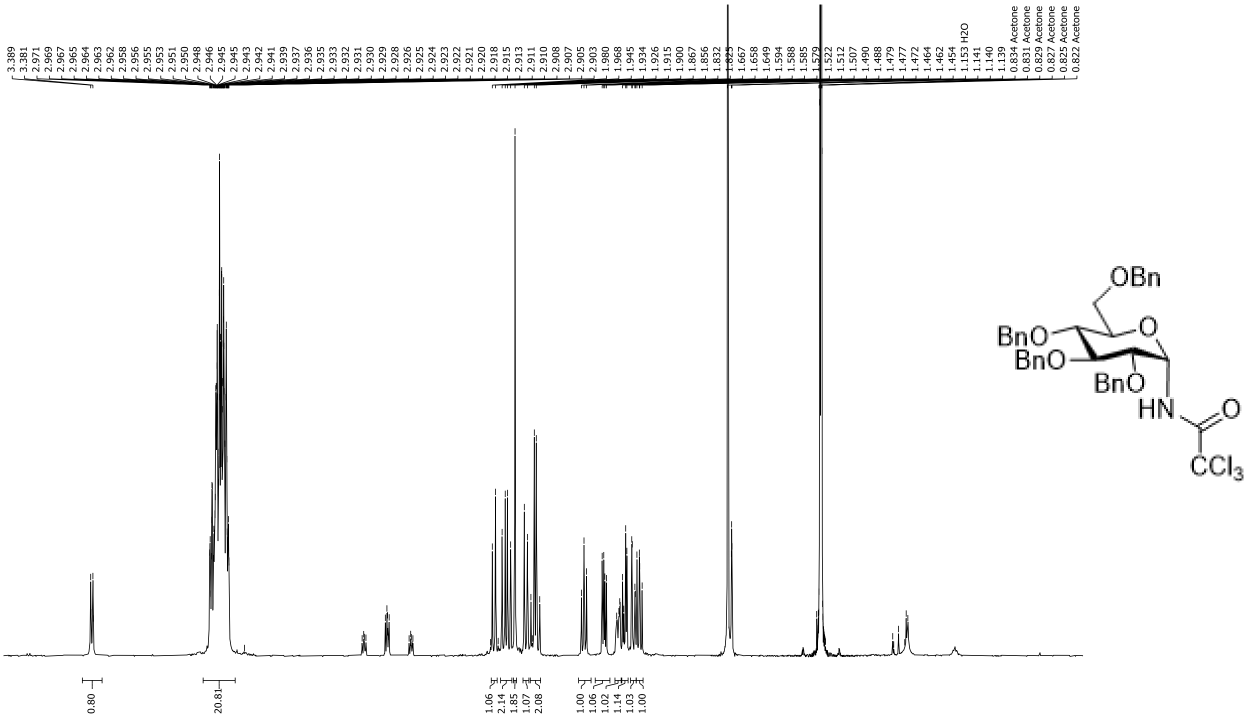

^{1}H NMR crude of N-trichloroacetyl-2,3,4,6-tetra-O-benzyl-α-D-glucopyranosylamide (8) (400 MHz, $CDCl_3$)

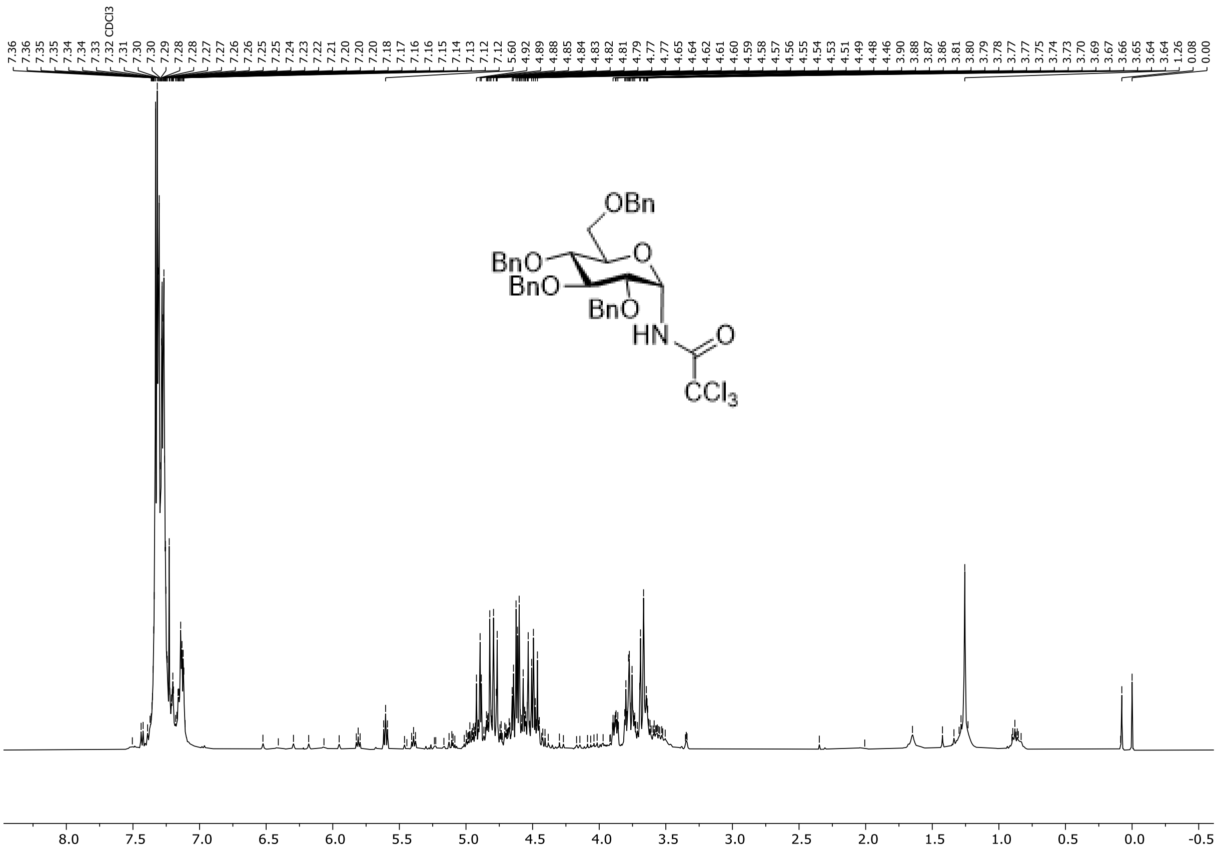

^{13}C NMR of crude N-trichloroacetyl-2,3,4,6-tetra-O-benzyl-α-D-glucopyranosylamide (8) (101 MHz, $CDCl_3$)

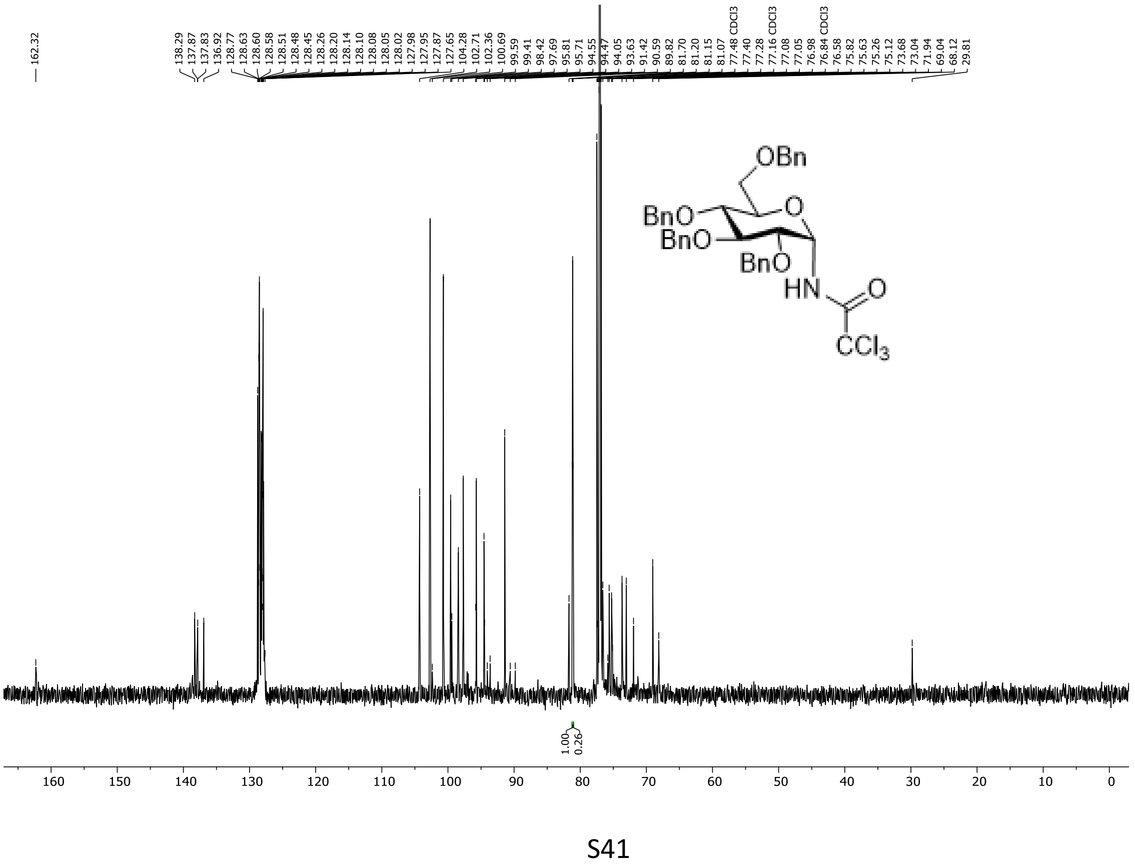

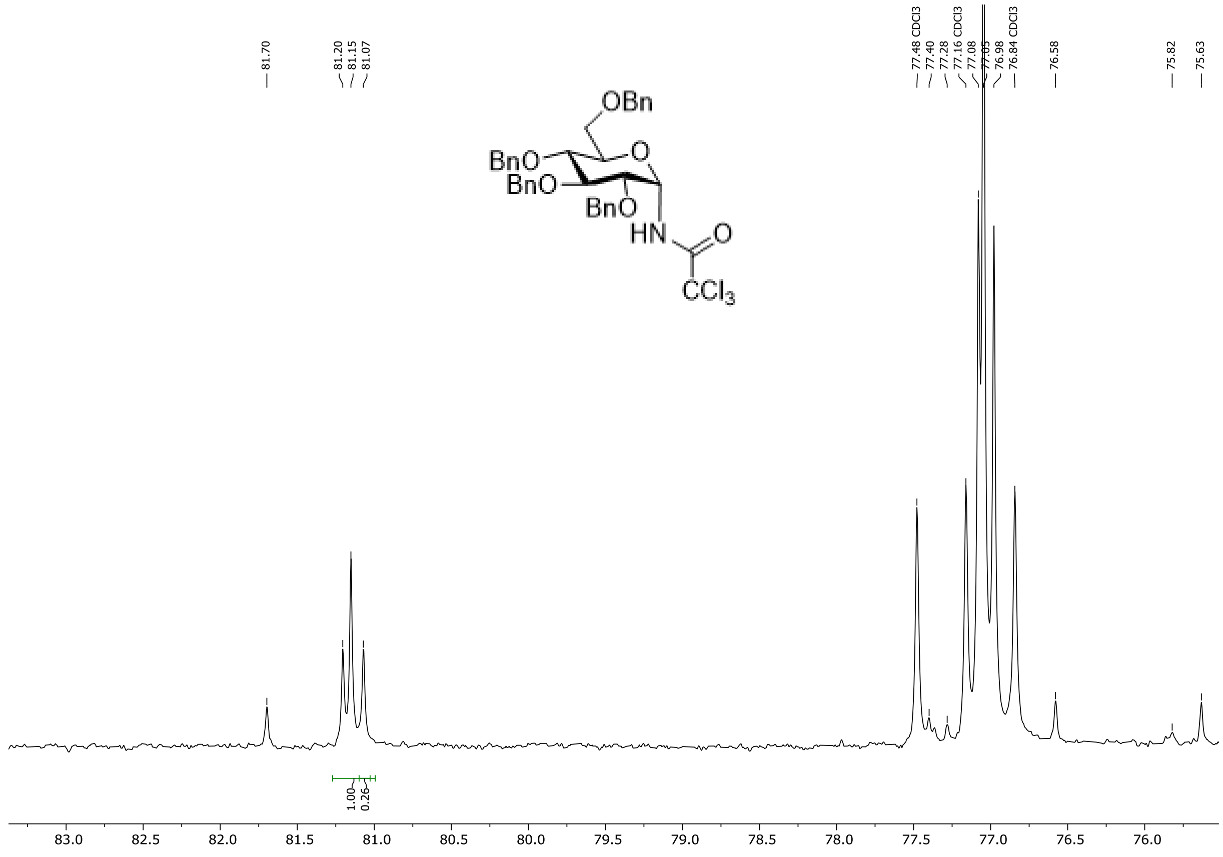

^{1}H NMR of N-trichloroacetyl-2,3,4,6-tetra-O-benzyl-α/β-D-mannopyranosylamide (9) (400 MHz, Acetone-d6)

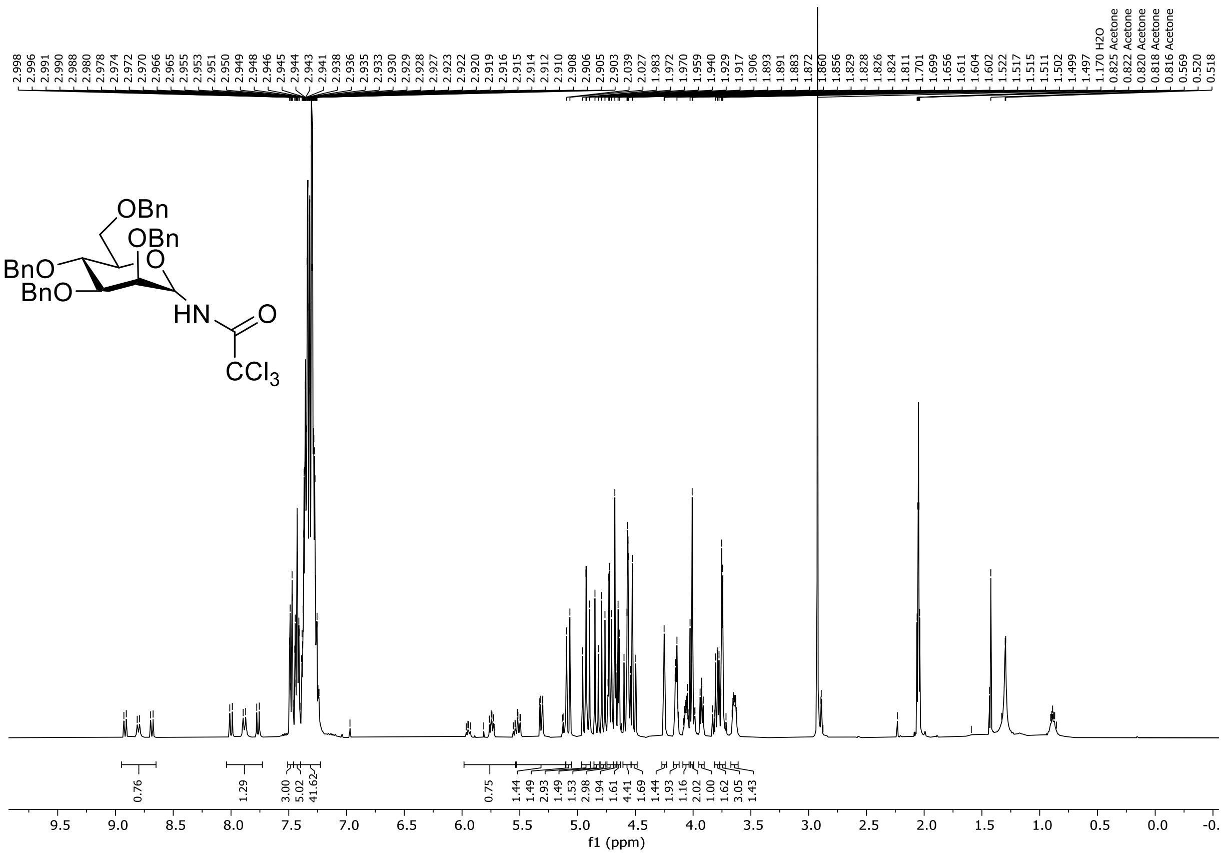

^{13}C NMR of N-trichloroacetyl-2,3,4,6-tetra-O-benzyl-α/β-D-mannopyranosylamide (9) (101 MHz, Acetone-d6)

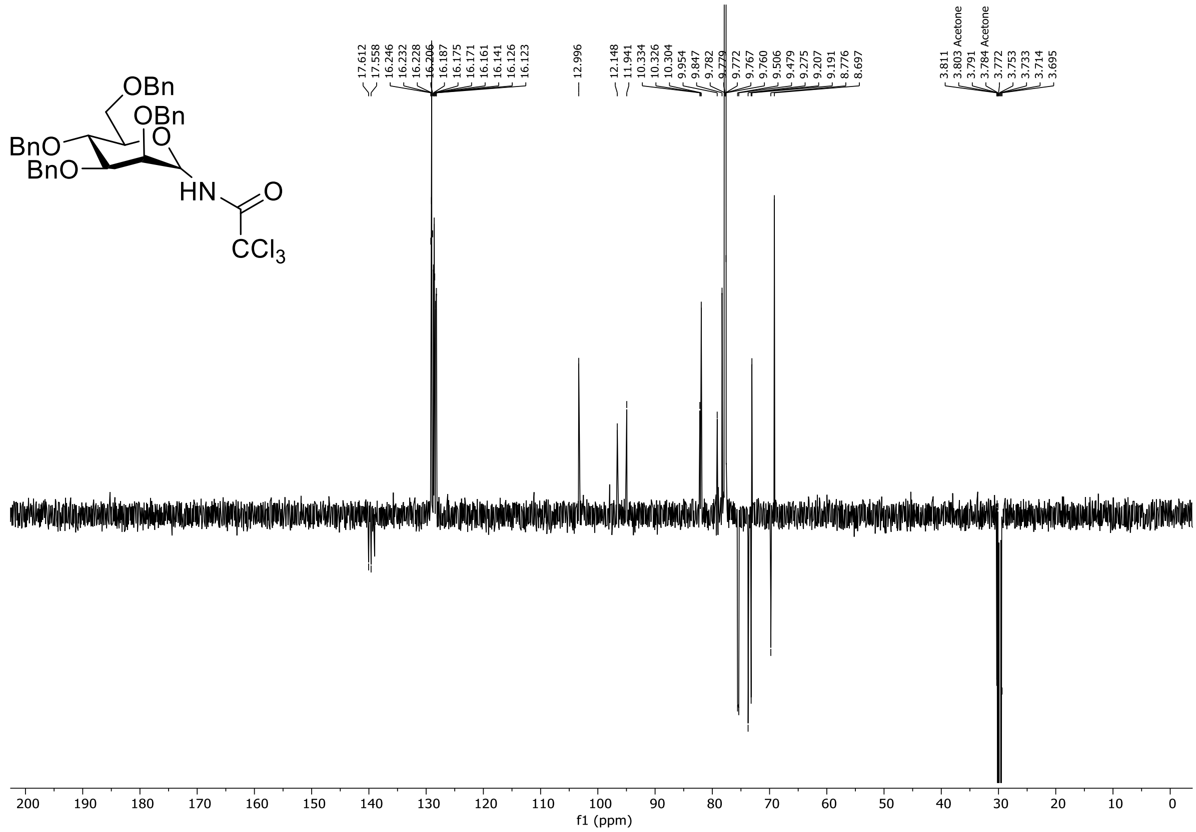

^{13}C NMR (Inverse Gated) of N-trichloroacetyl-2,3,4,6-tetra-O-benzyl-α/β-D-mannopyranosylamide (9) (101 MHz, Toluene-d8)

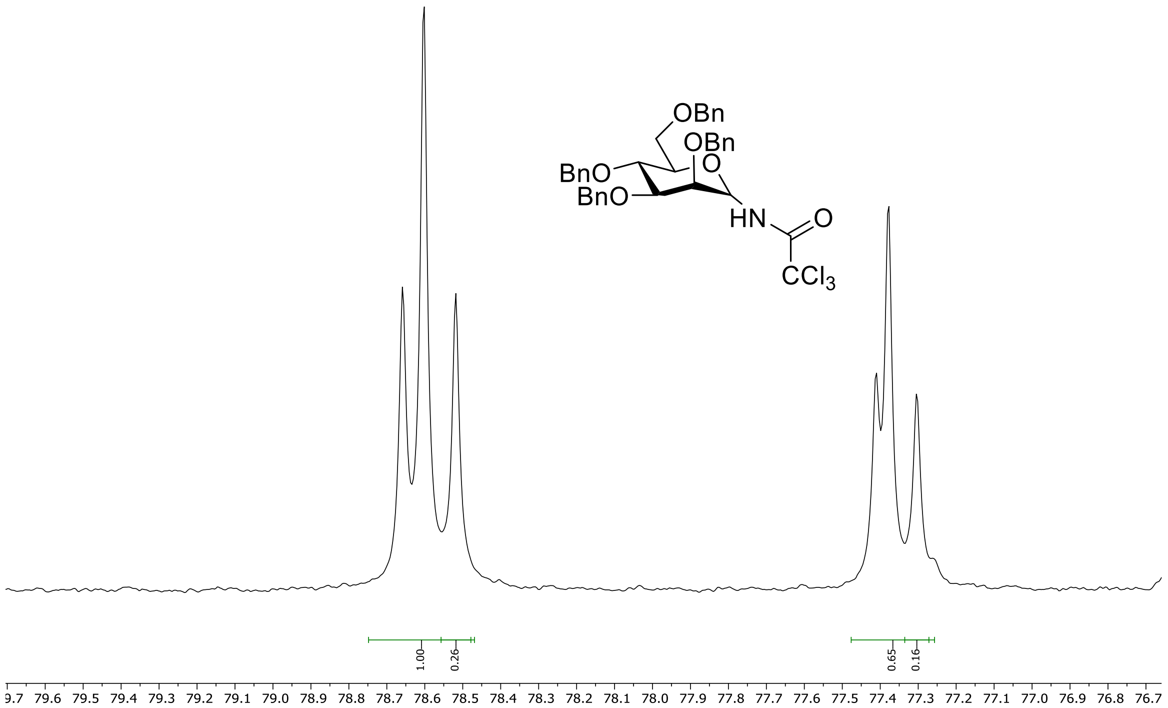

^{15}N NMR of N-trichloroacetyl-2,3,4,6-tetra-O-benzyl-α/β-D-mannopyranosylamide (9) (41 MHz, Acetone-d6)

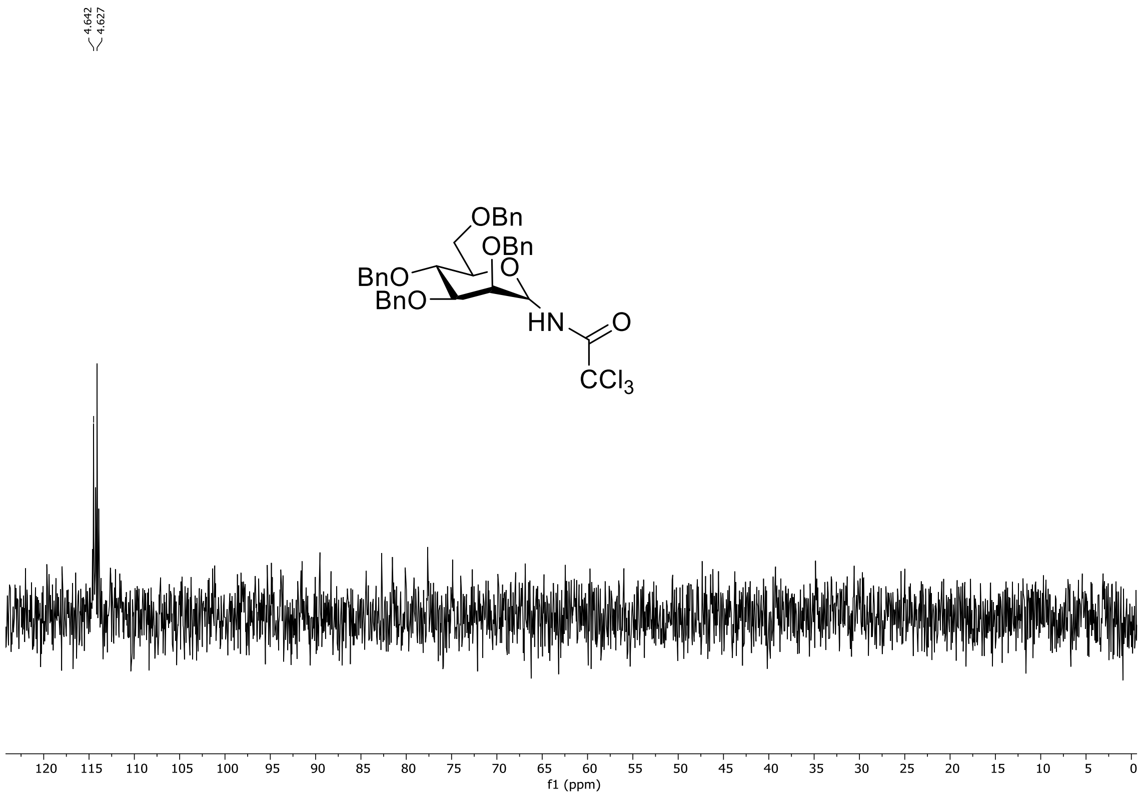

HH COSY of N-trichloroacetyl-2,3,4,6-tetra-O-benzyl-α/β-D-mannopyranosylamide (9) (Acetone-d6)

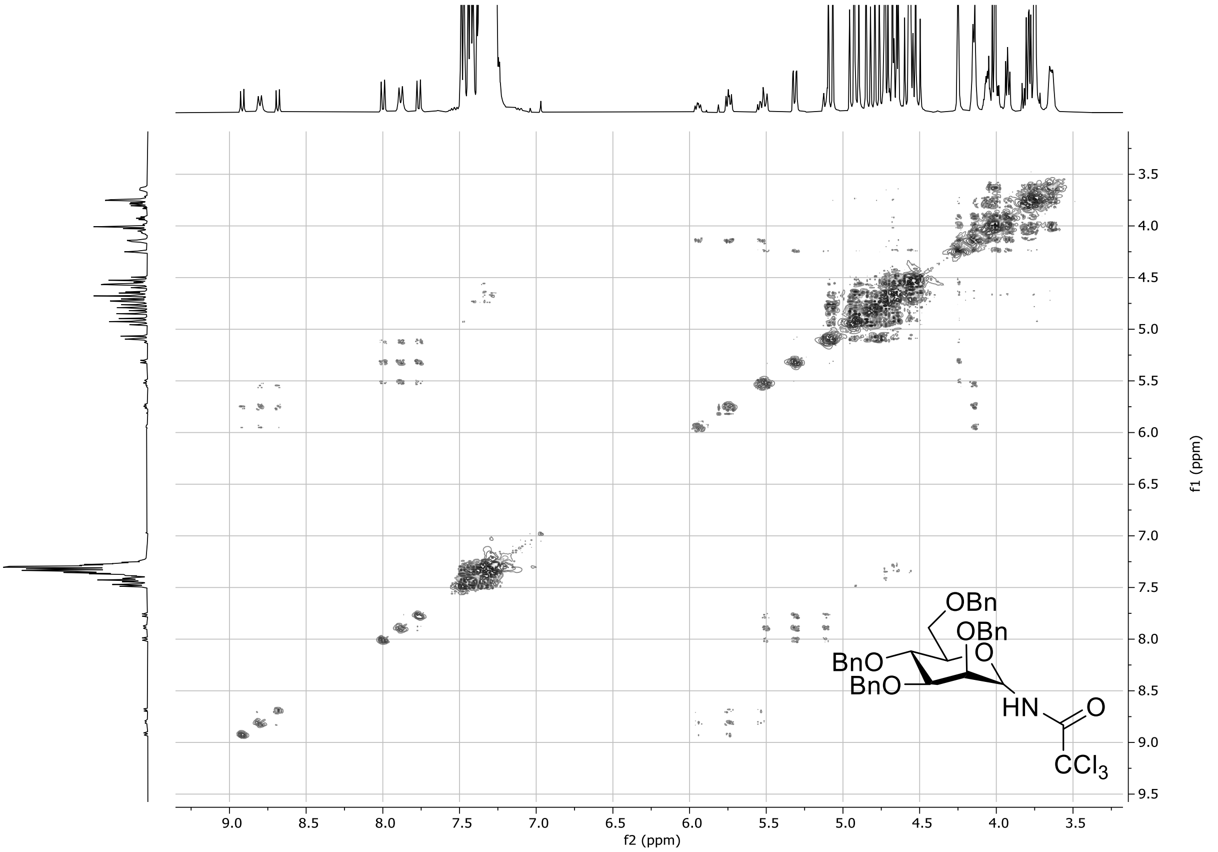

CH HSQC of N-trichloroacetyl-2,3,4,6-tetra-O-benzyl-α/β-D-mannopyranosylamide (9) (Acetone-d6)

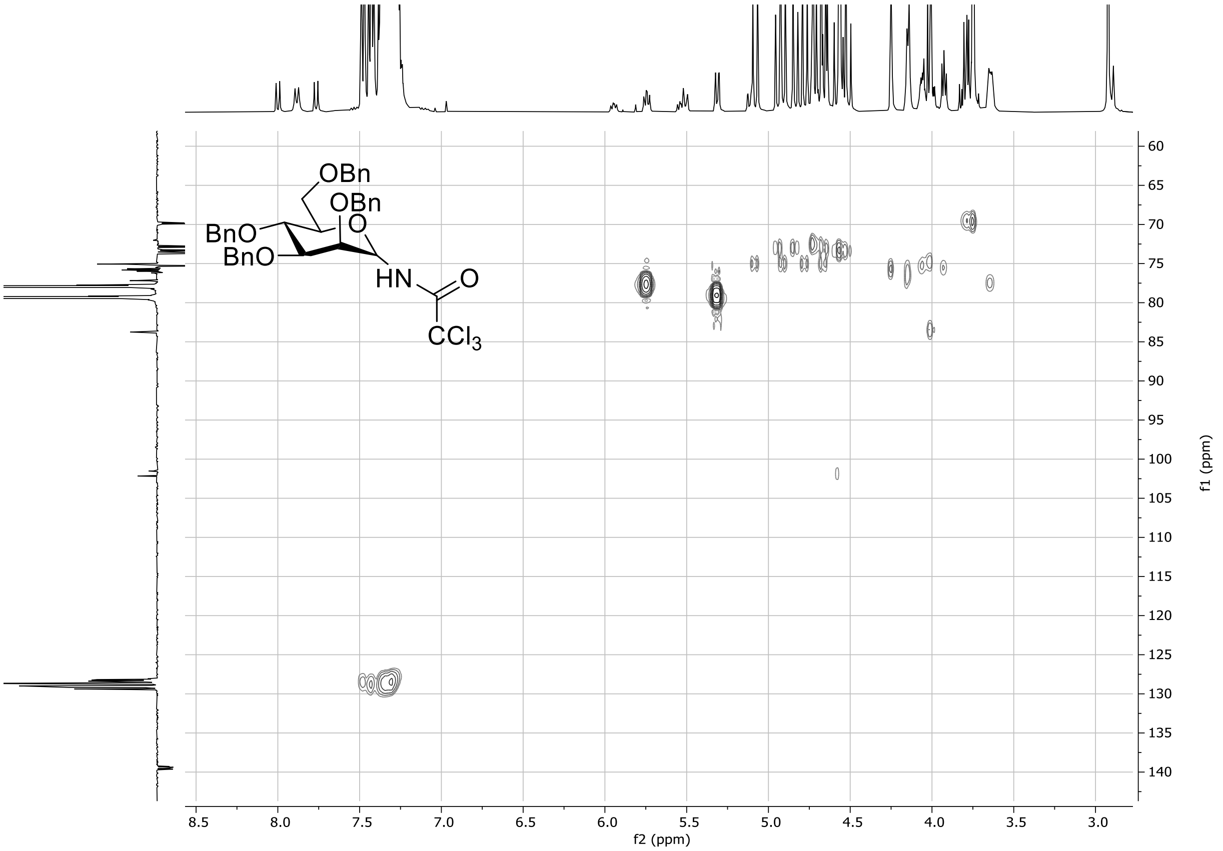

NH HSQC of N-trichloroacetyl-2,3,4,6-tetra-O-benzyl-α/β-D-mannopyranosylamide (9) (Acetone-d6)

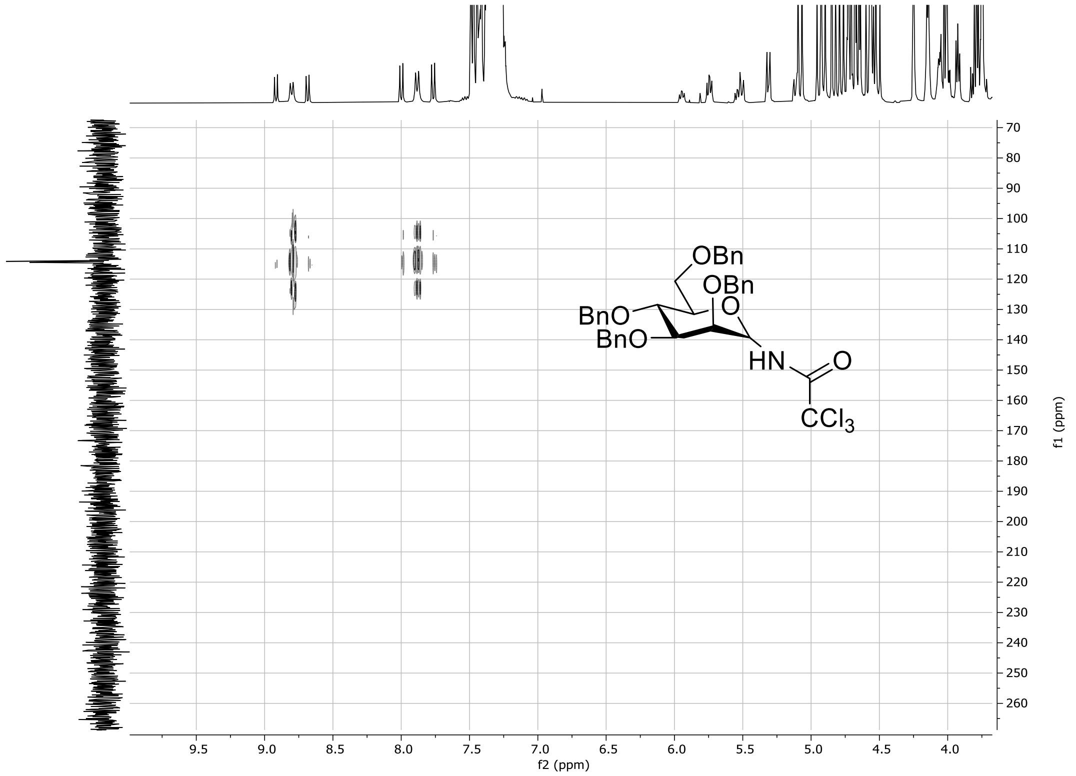

NH HMBC of N-trichloroacetyl-2,3,4,6-tetra-O-benzyl-α/β-D-mannopyranosylamide (9) (Acetone-d6)

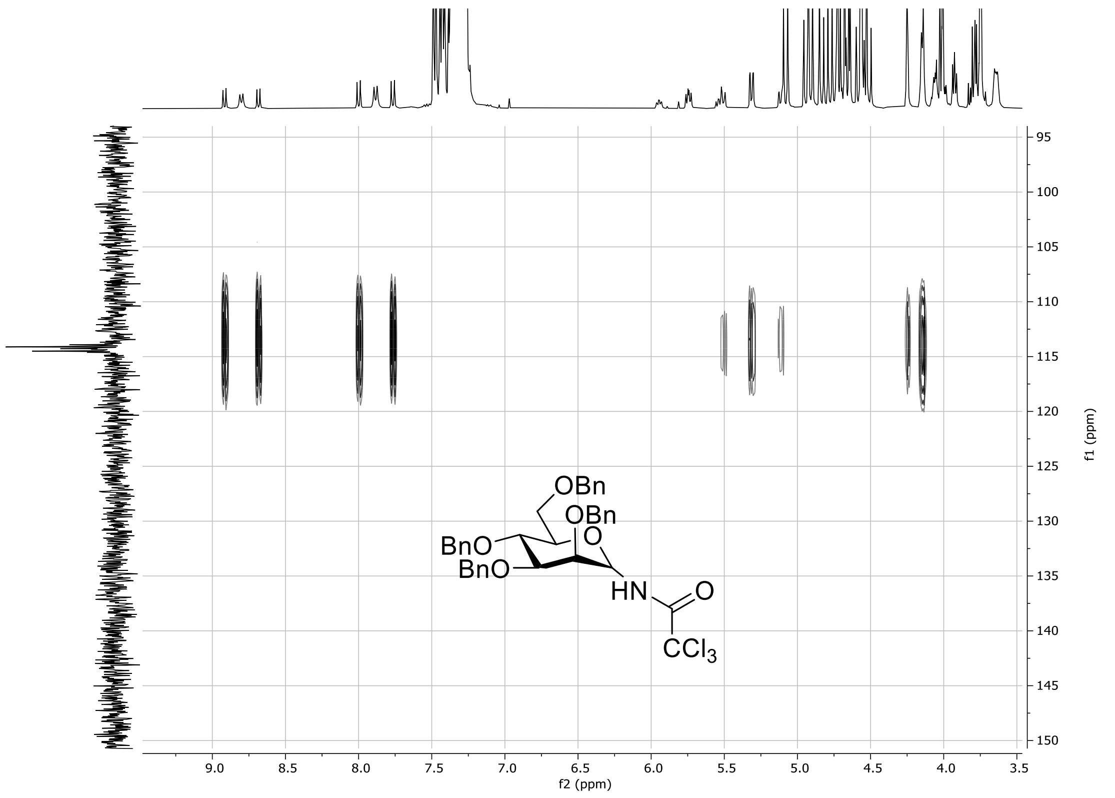

^1H NMR (^{13}C decoupled) of N-trichloroacetyl-2,3,4,6-tetra-O-benzyl-α/β-D-mannopyranosylamide (9) (Acetone-d6)

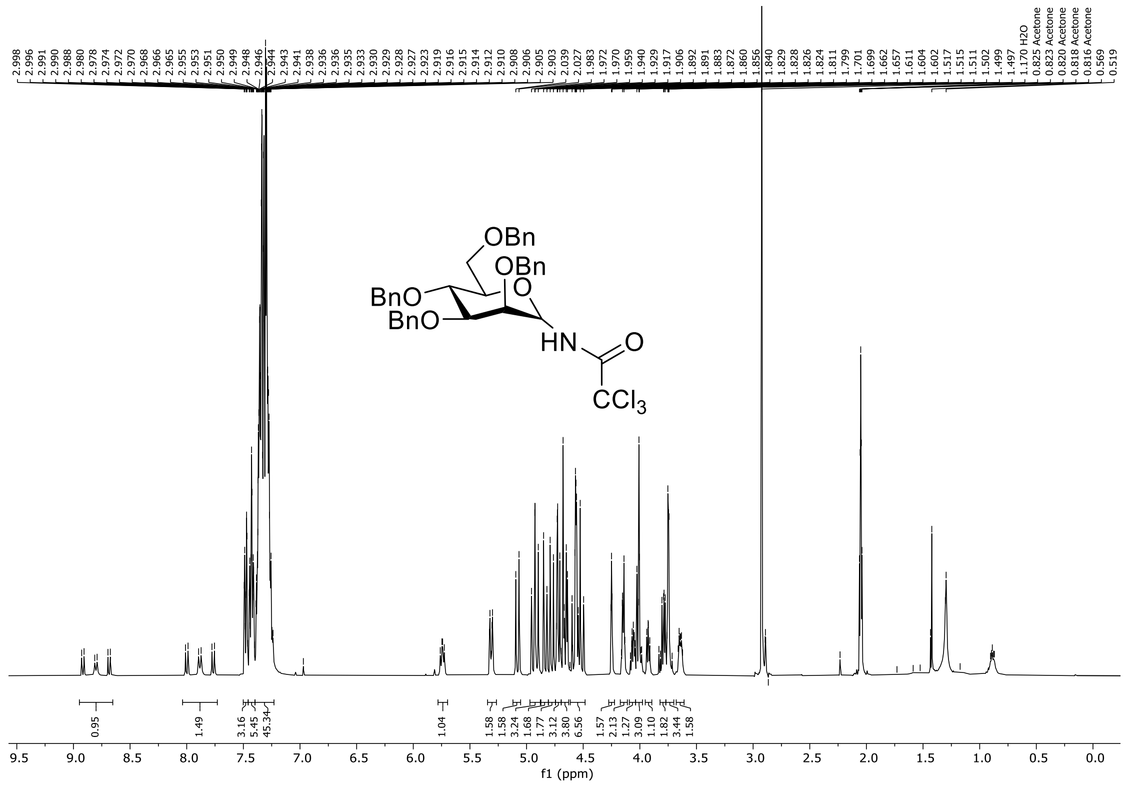

^1H NMR (^{15}N decoupled) of N-trichloroacetyl-2,3,4,6-tetra-O-benzyl-α/β-D-mannopyranosylamide (9) (Acetone-d6)

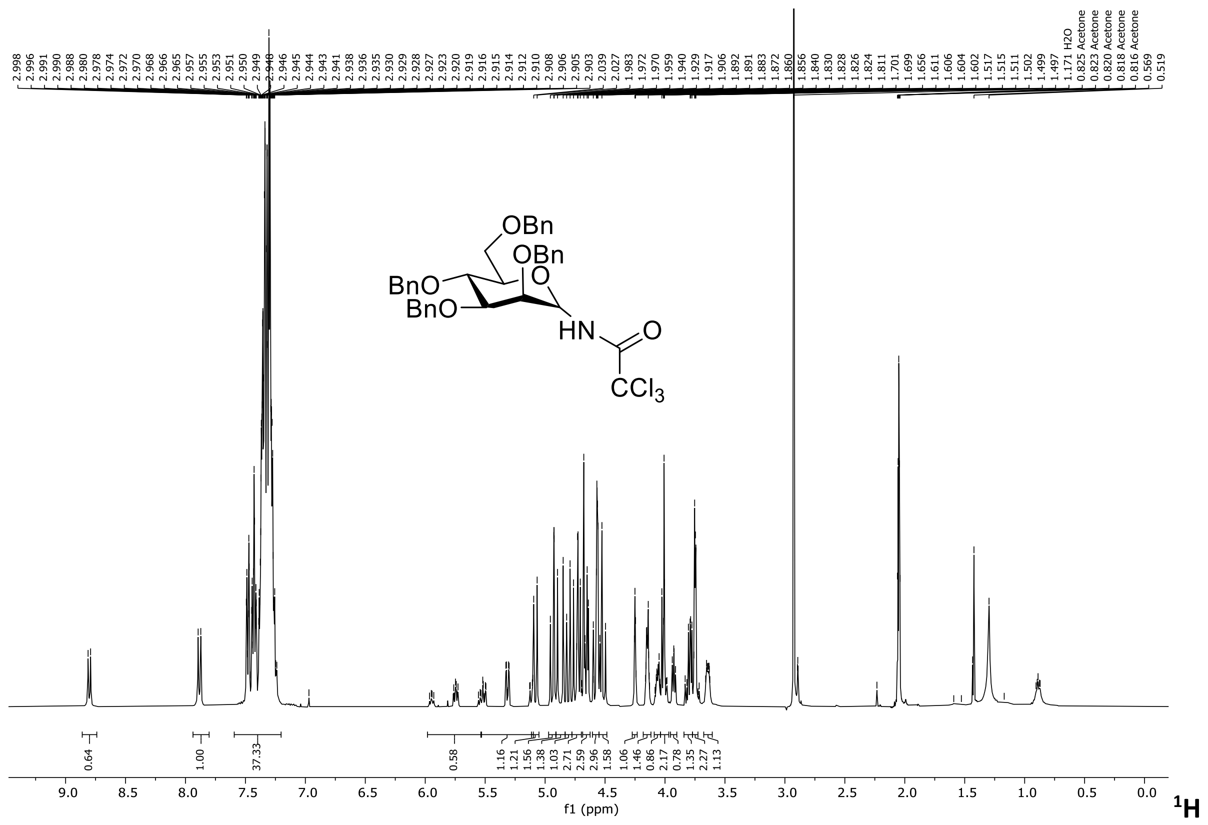

^{1}H NMR of crude N-trichloroacetyl-2,3,4,6-tetra-O-benzyl-α/β-D-mannopyranosylamide (9) (400 MHz, Acetone-d6)

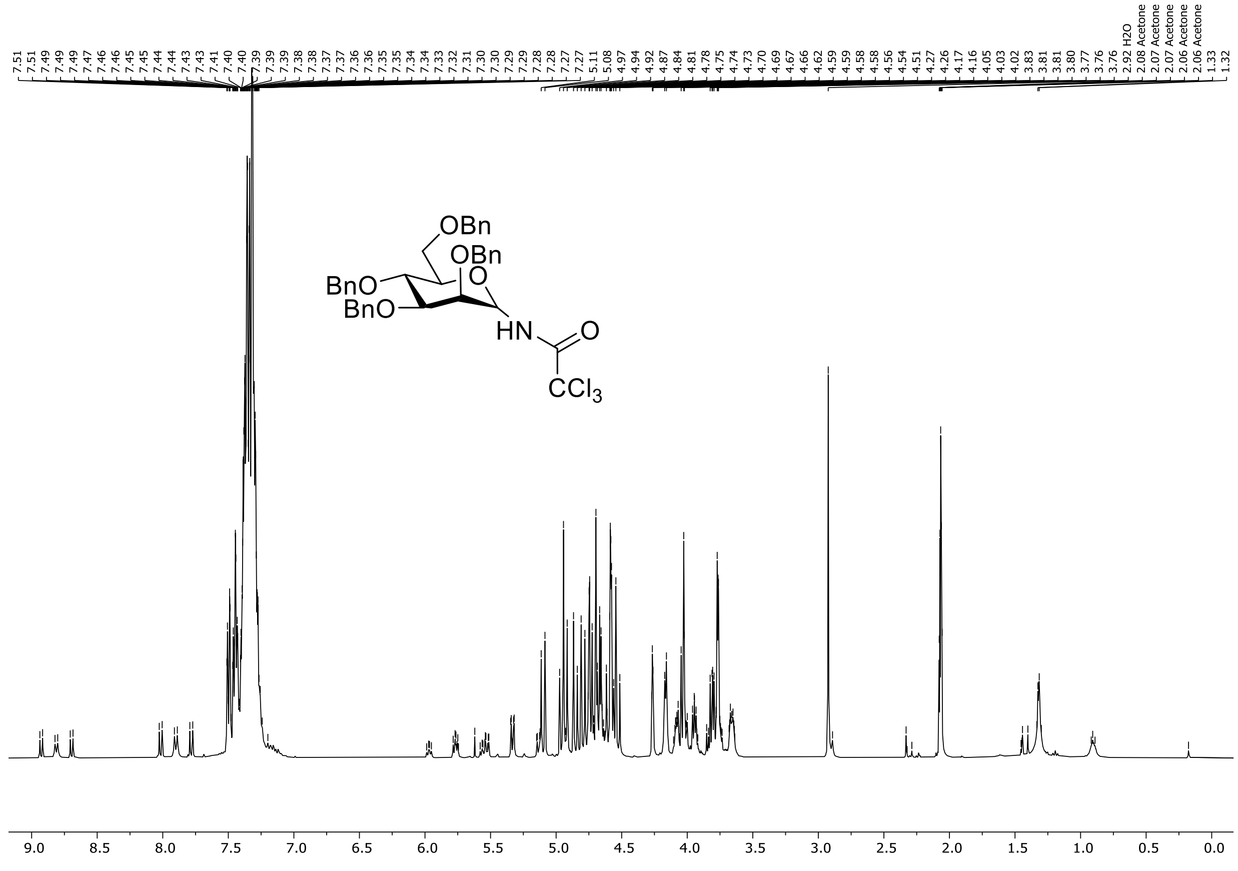

^{13}C NMR of crude N-trichloroacetyl-2,3,4,6-tetra-O-benzyl-α/β-D-mannopyranosylamide (9) (101 MHz, Acetone-d6)

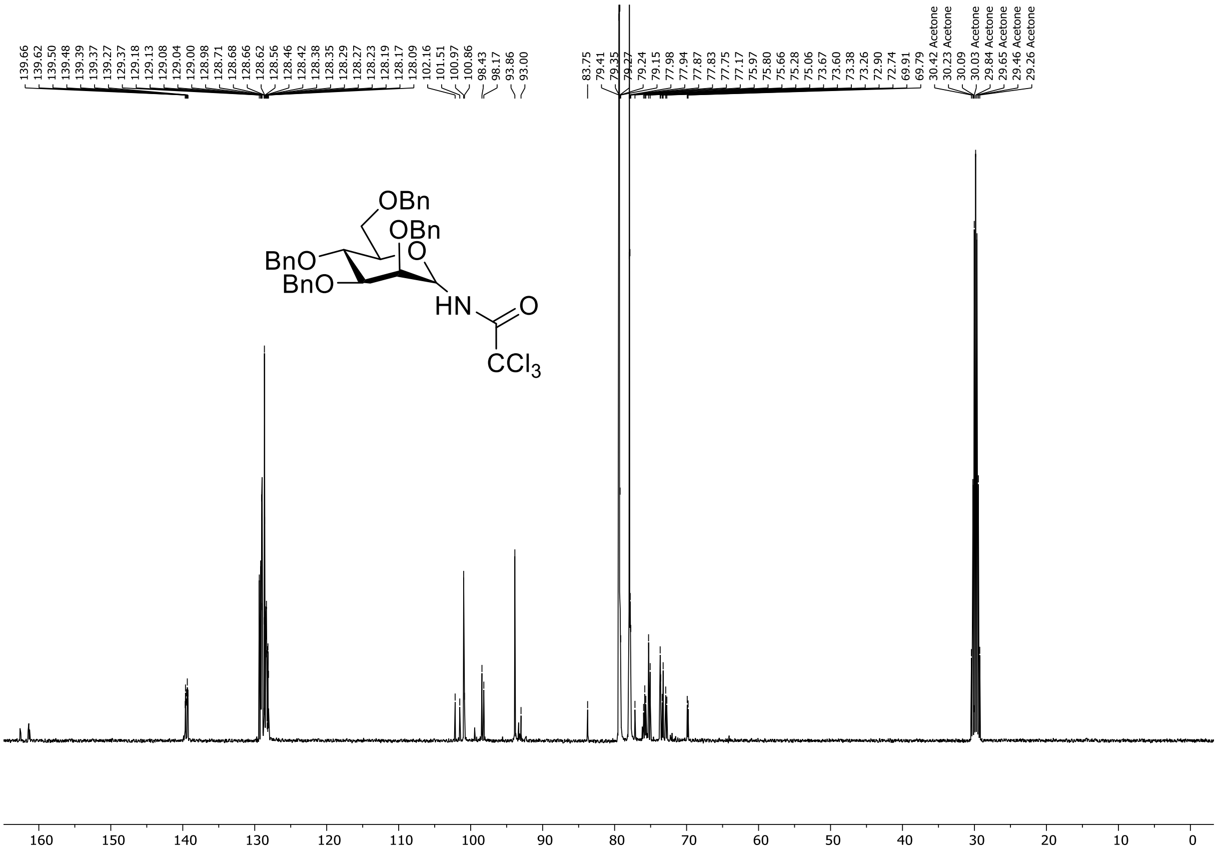

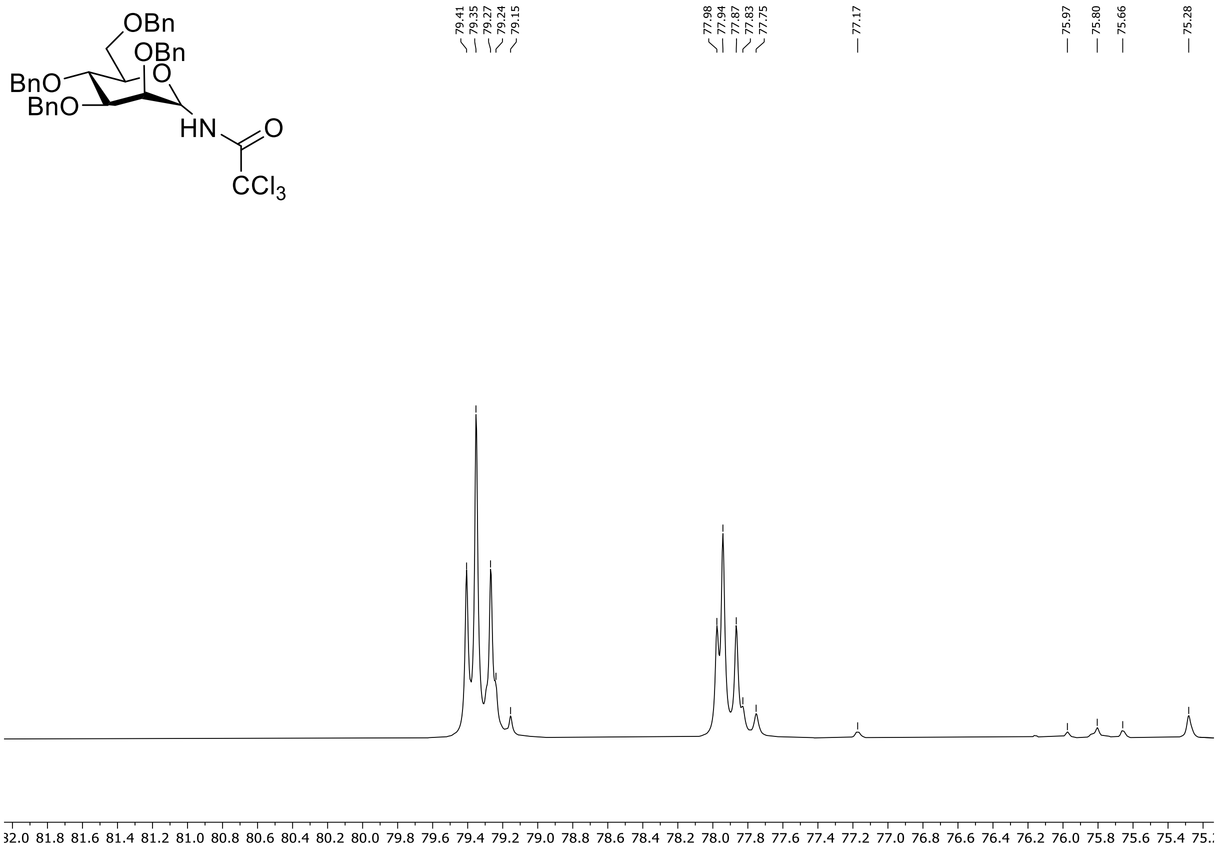

^{1}H NMR of N-trichloroacetyl-2,3,4,6-tetra-O-benzyl-α-D-glucopyranosylamide (8) (1 eq Cl_3CCONH_2) (400 MHz, Acetone-d6)

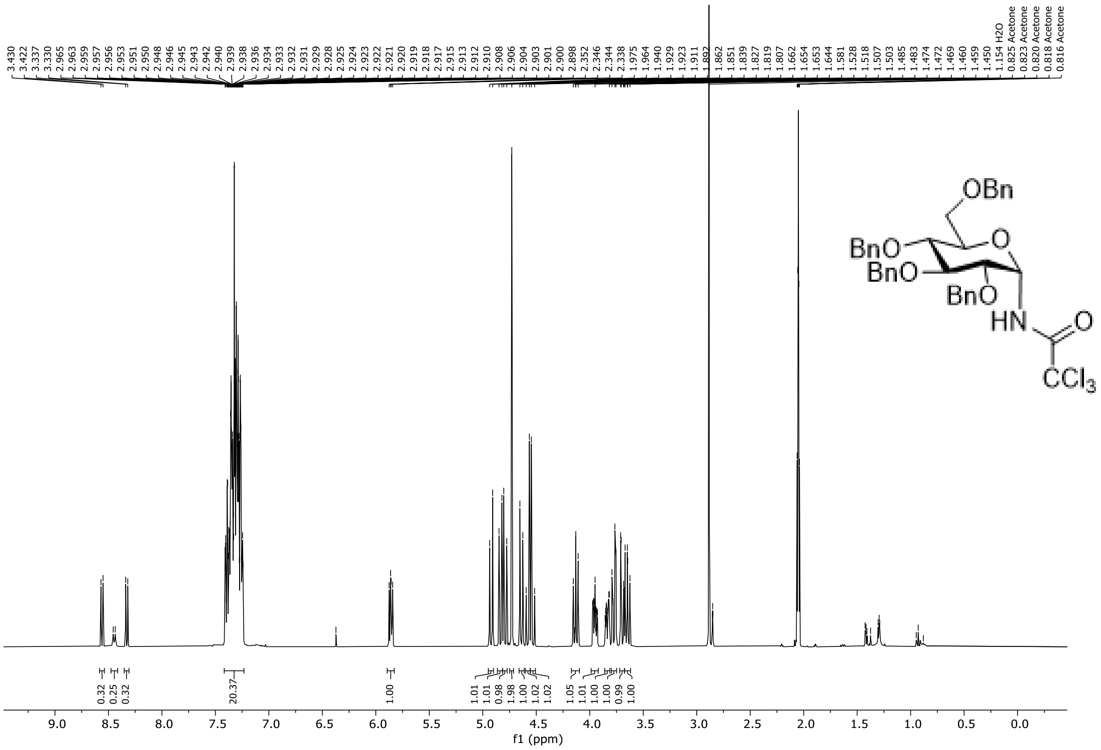

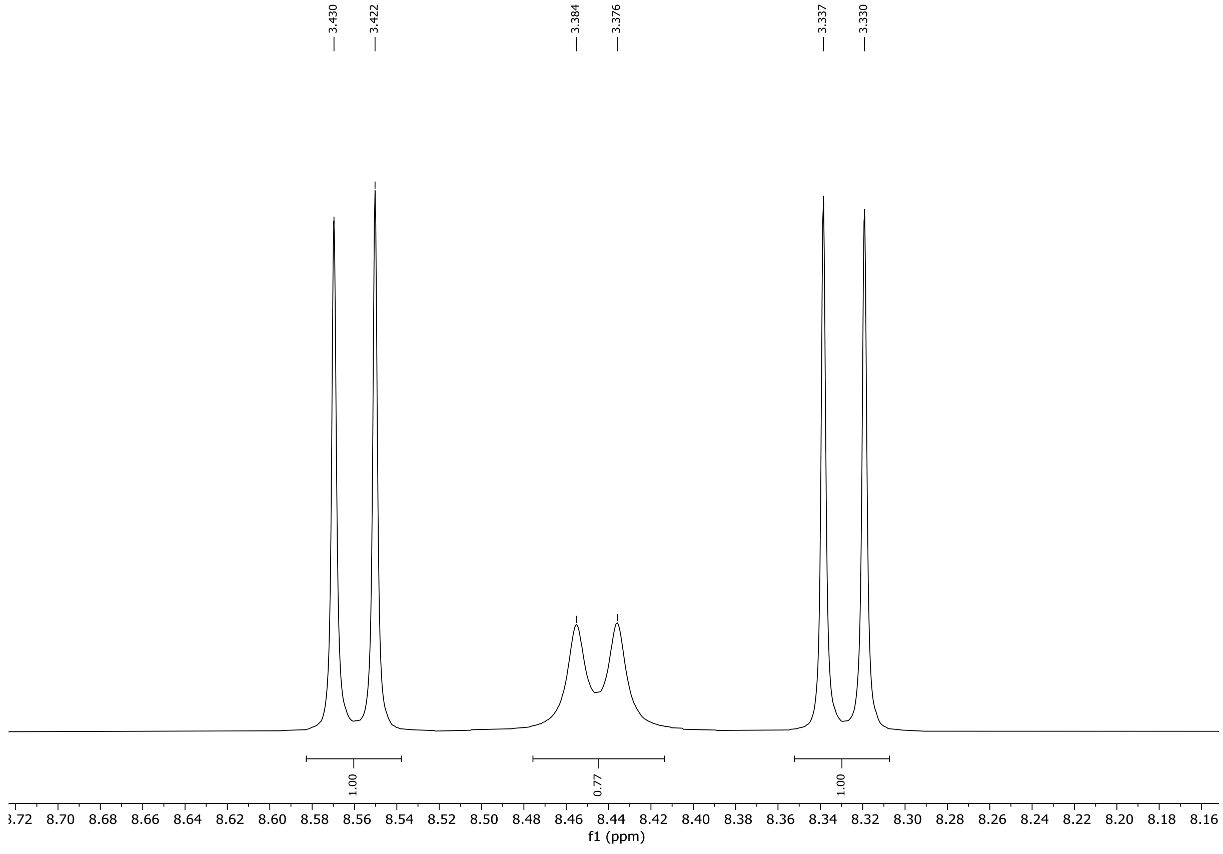

^{13}C NMR of N-trichloroacetyl-2,3,4,6-tetra-O-benzyl-α-D-glucopyranosylamide (8) (1 eq Cl_3CCONH_2) (101 MHz, Acetone-d6)

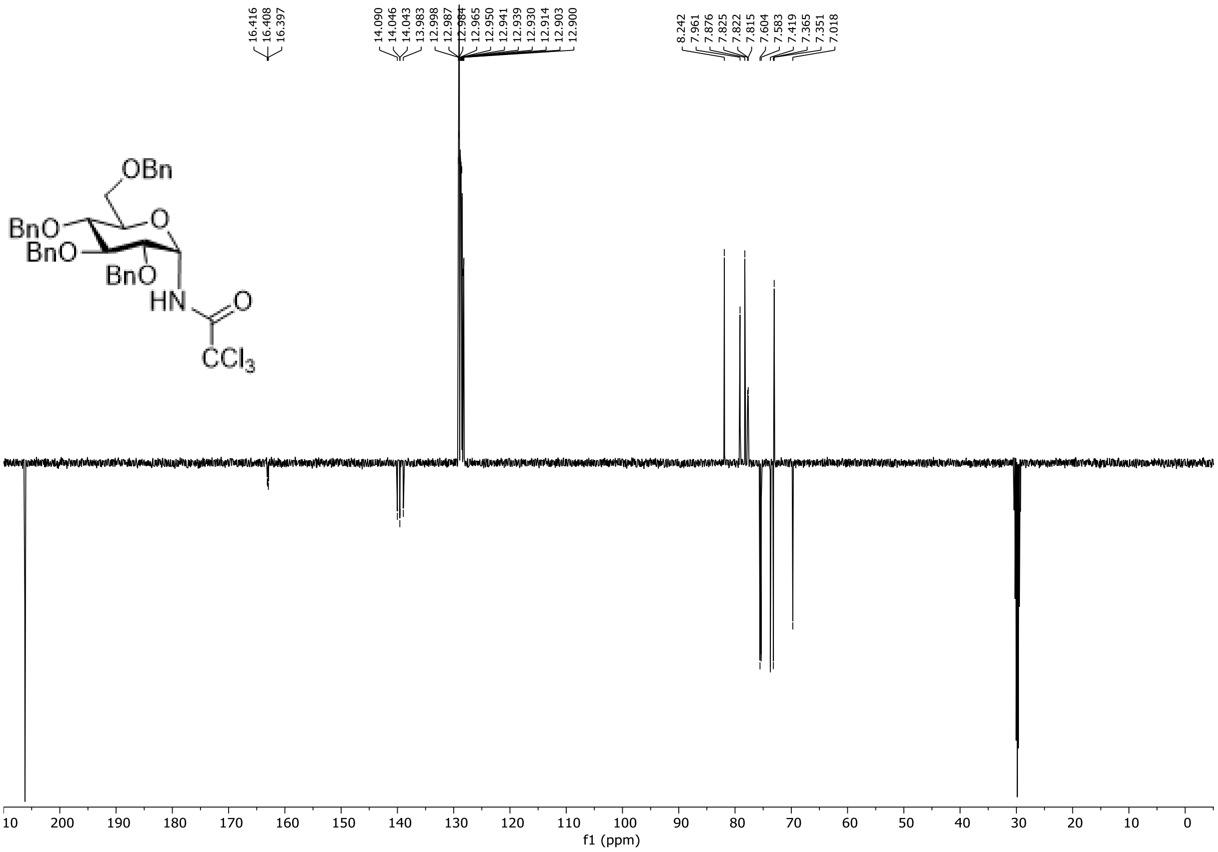

^{15}N NMR of N-trichloroacetyl-2,3,4,6-tetra-O-benzyl-α-D-glucopyranosylamide (8) (1 eq Cl_3CCONH_2) (41 MHz, Acetone-d6)

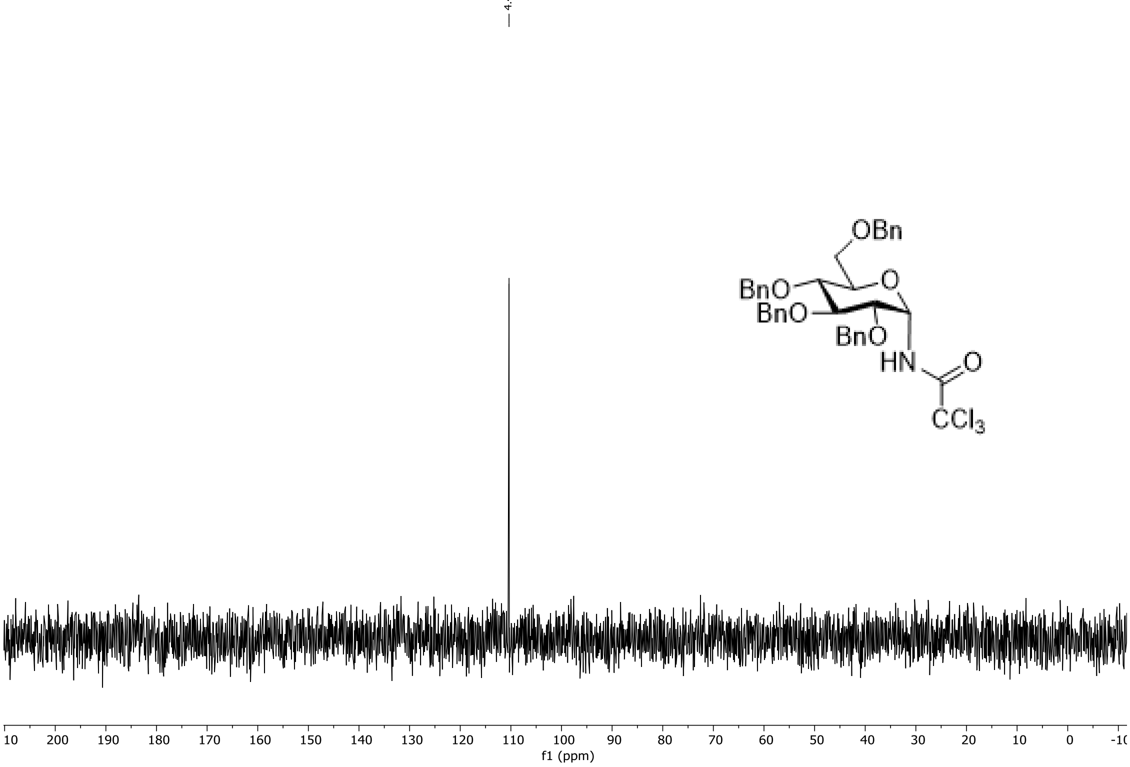

HH COSY of N-trichloroacetyl-2,3,4,6-tetra-O-benzyl-α-D-glucopyranosylamide (8) (1 eq Cl_3CCONH_2) (Acetone-d6)

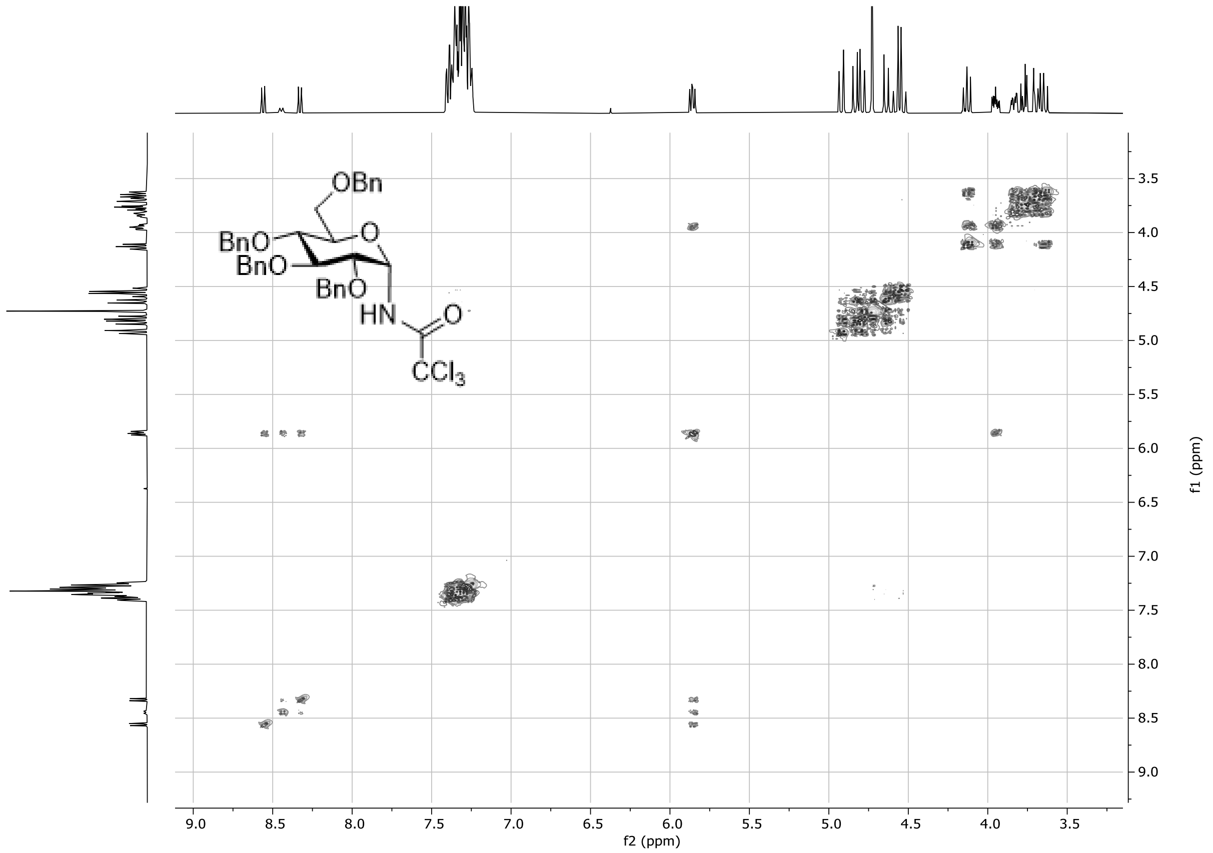

CH HSQC of N-trichloroacetyl-2,3,4,6-tetra-O-benzyl-α-D-glucopyranosylamide (8) (1 eq Cl_3CCONH_2) (Acetone-d6)

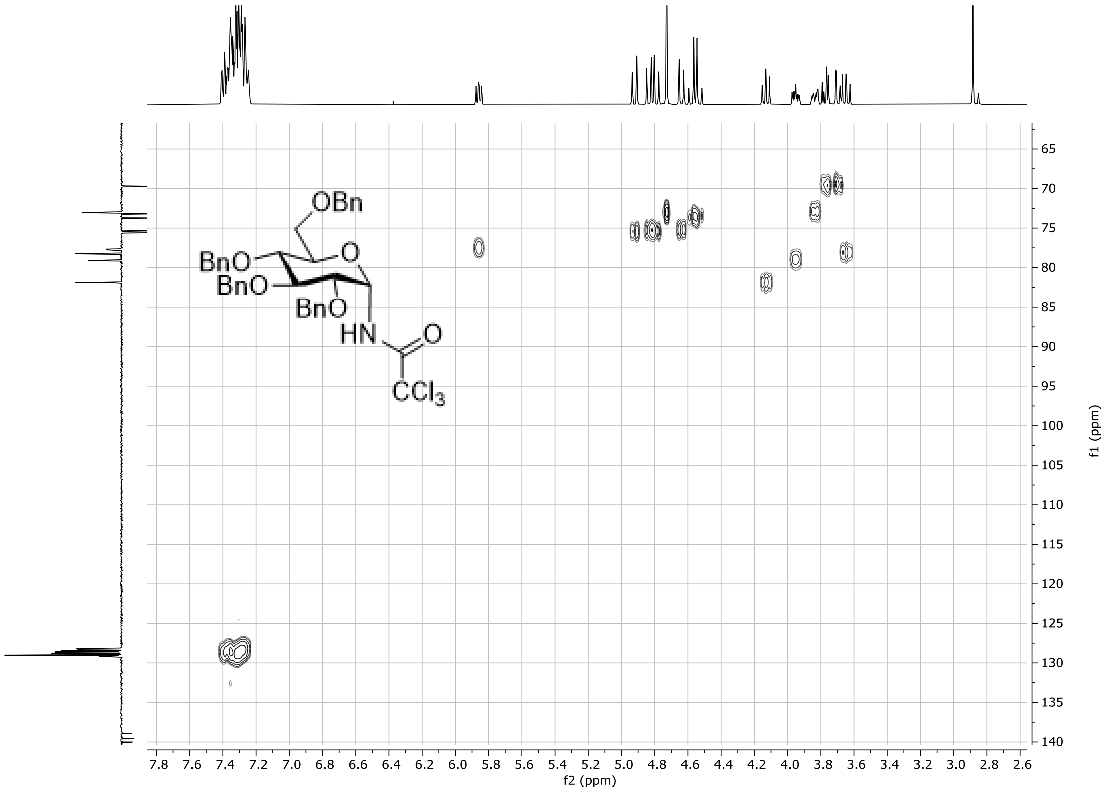

^{1}H NMR of N-trichloroacetyl-2,3,4,6-tetra-O-benzyl-α-D-glucopyranosylamide (8) (3 eq Cl_3CCONH_2) (400 MHz, Acetone-d6)

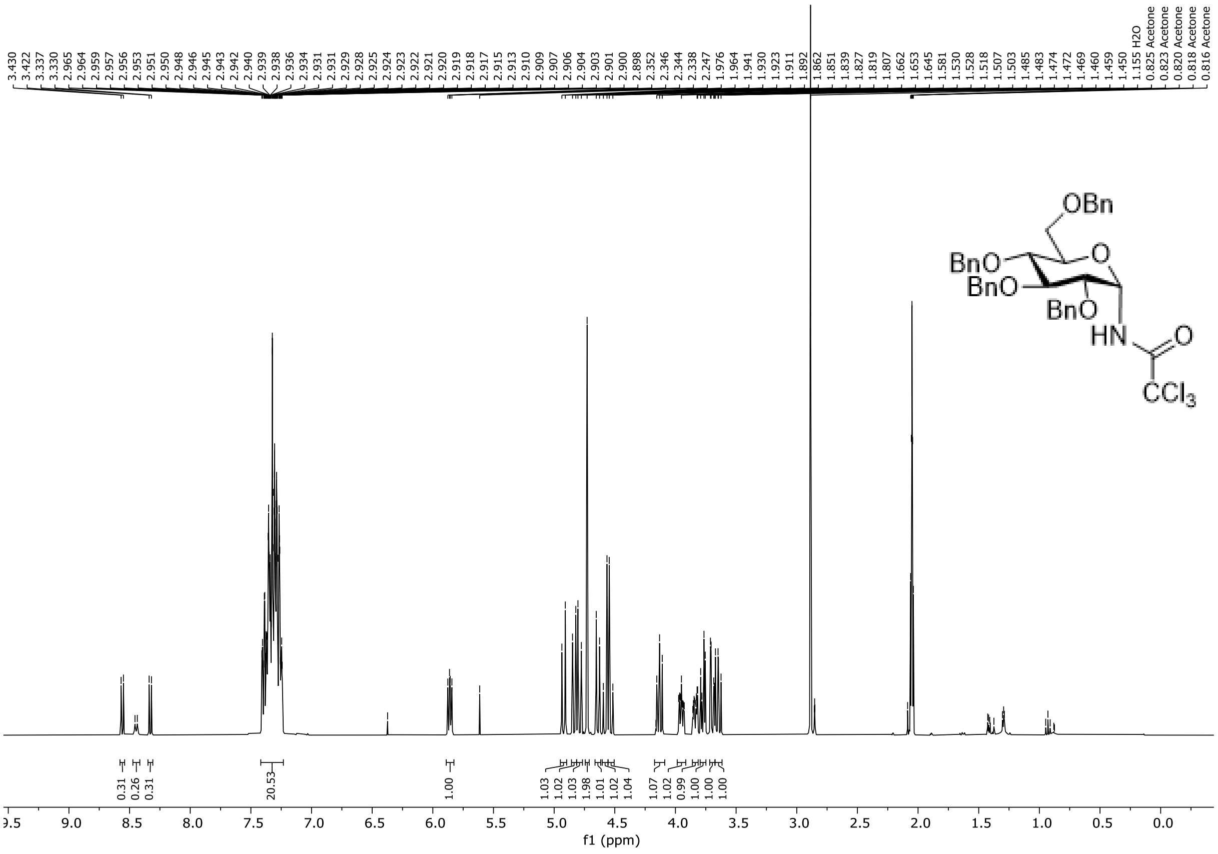

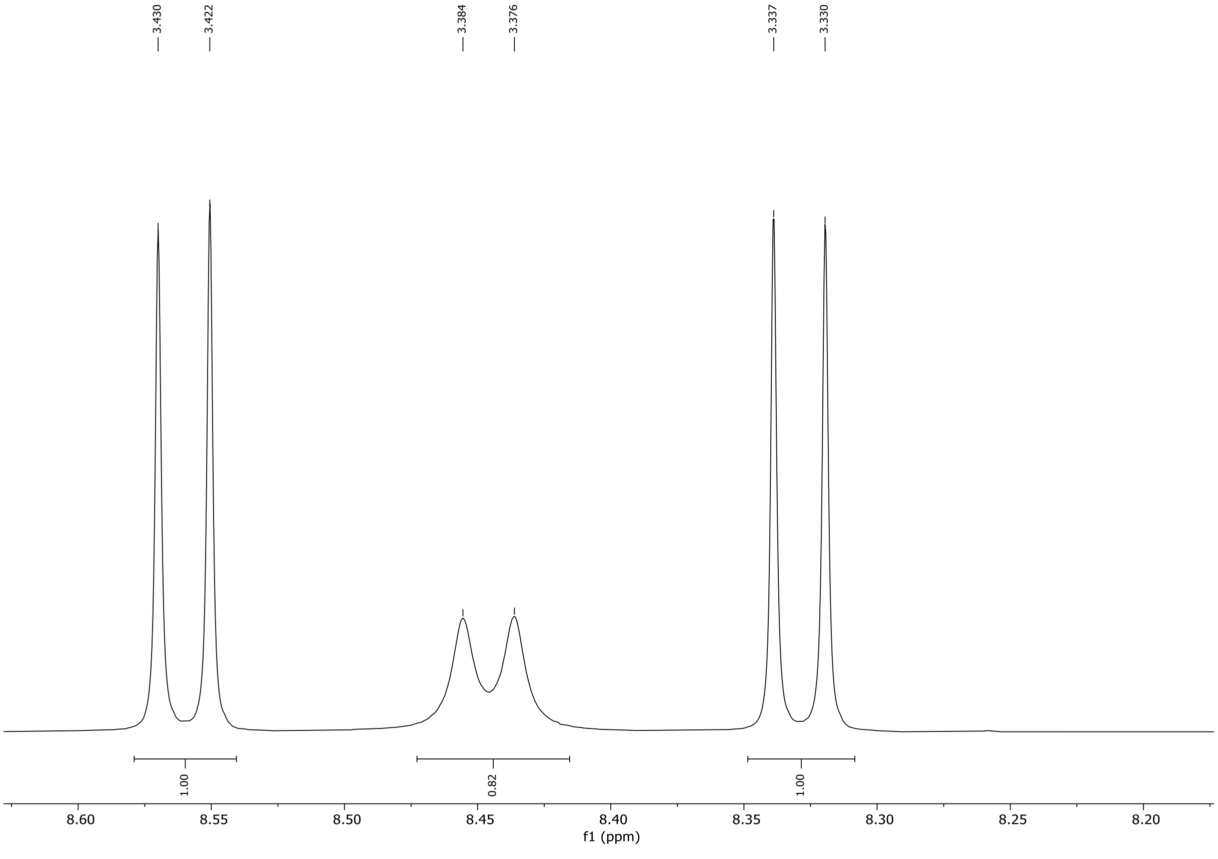

^{13}C NMR of N-trichloroacetyl-2,3,4,6-tetra-O-benzyl-α-D-glucopyranosylamide (8) (3 eq Cl_3CCONH_2) (101 MHz, Acetone-d6)

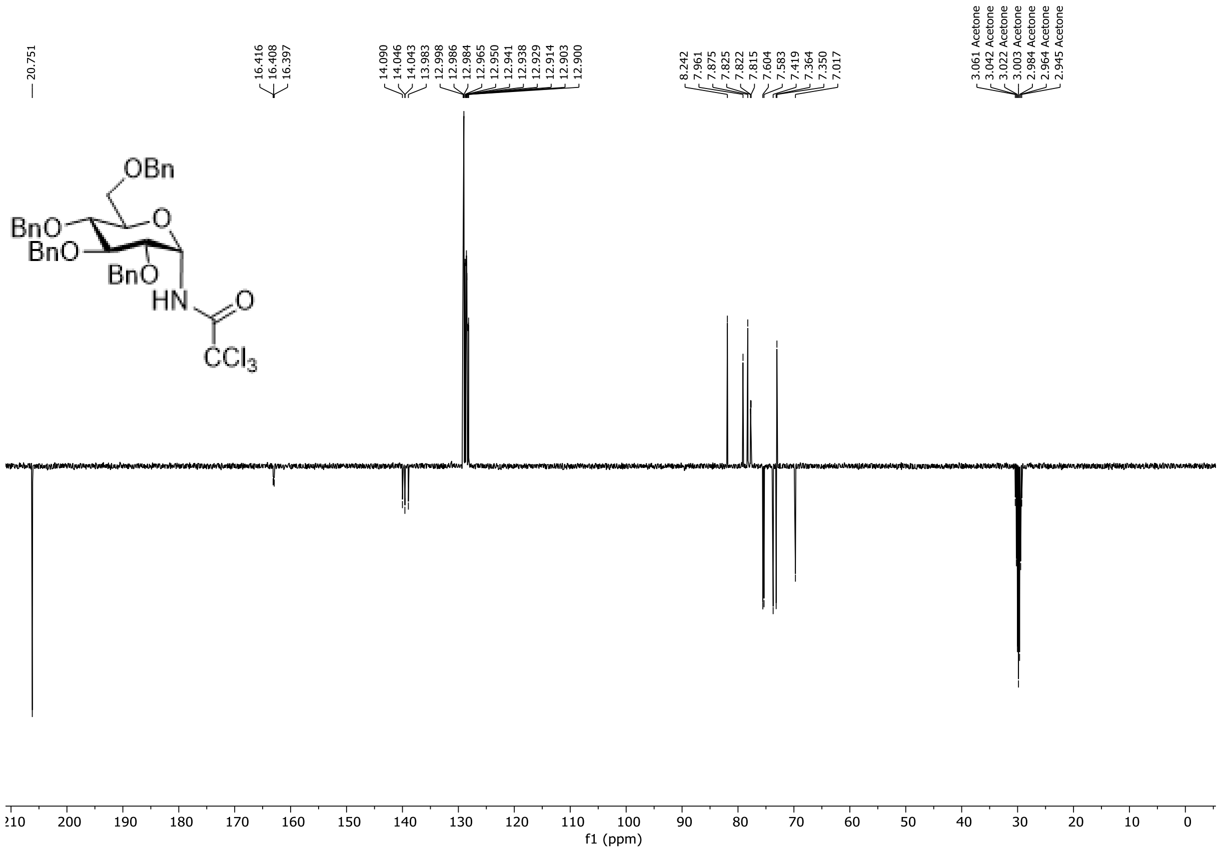

^{15}N NMR of N-trichloroacetyl-2,3,4,6-tetra-O-benzyl-α-D-glucopyranosylamide (8) (3 eq Cl_3CCONH_2) (41 MHz, Acetone-d6)

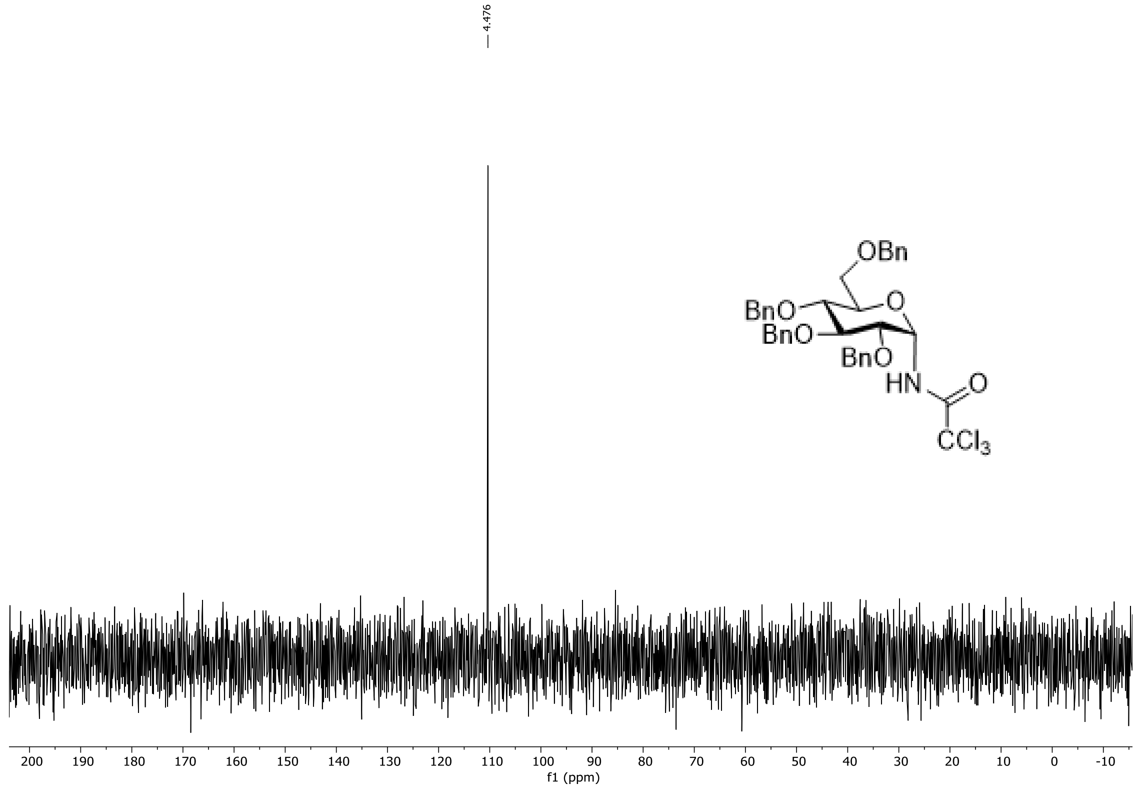

HH COSY of N-trichloroacetyl-2,3,4,6-tetra-O-benzyl-α-D-glucopyranosylamide (8) (3 eq Cl_3CCONH_2) (Acetone-d6)

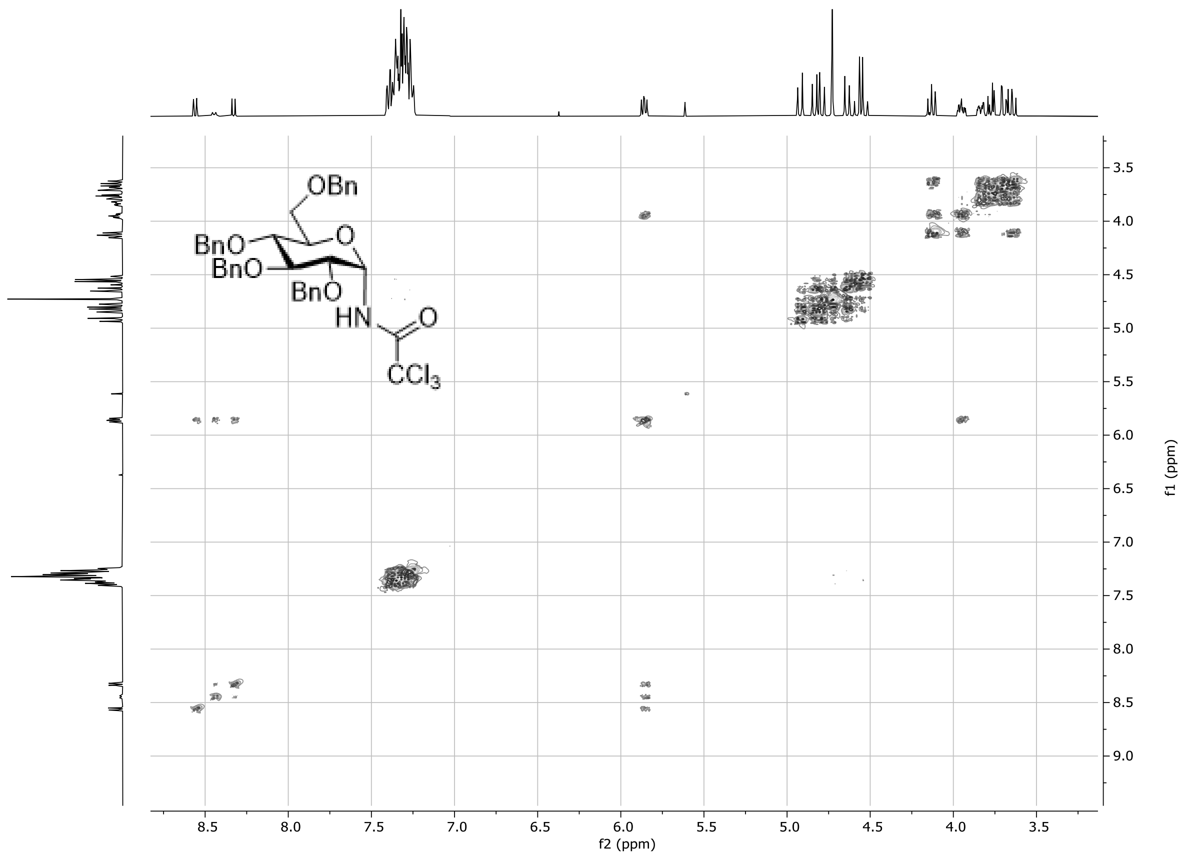

CH HSQC of N-trichloroacetyl-2,3,4,6-tetra-O-benzyl-α-D-glucopyranosylamide (8) (3 eq Cl_3CCONH_2) (Acetone-d6)

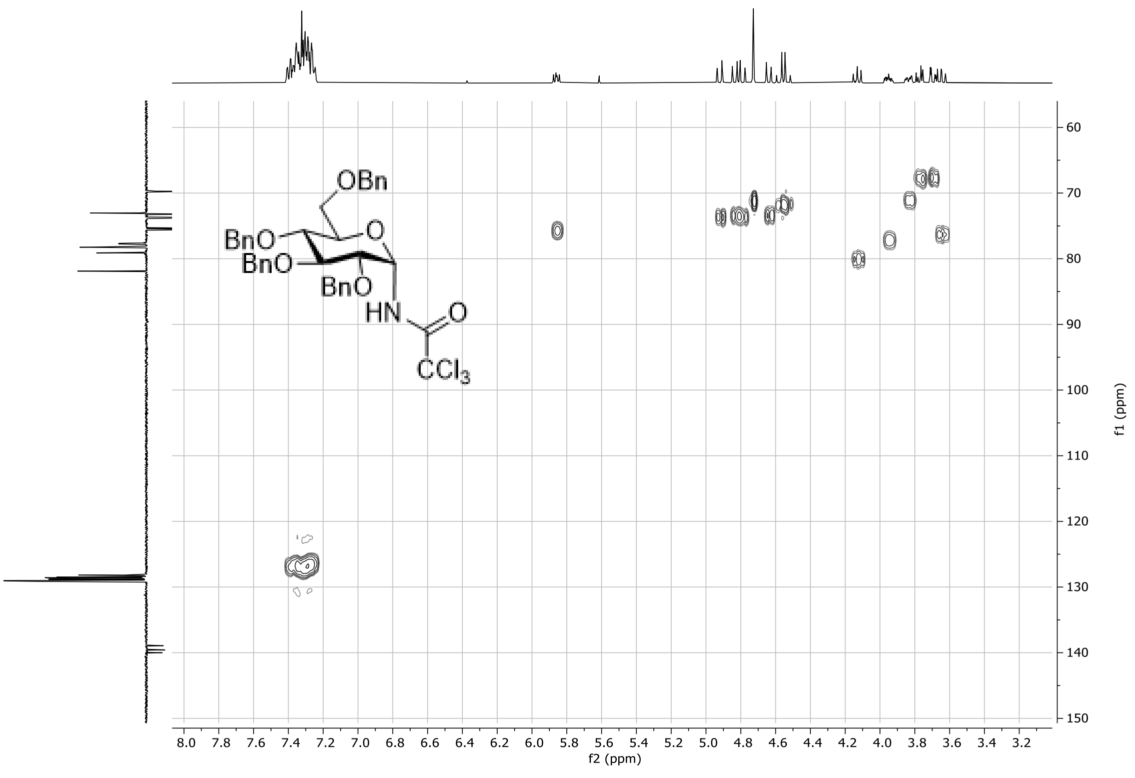

^{1}H NMR of N-trichloroacetyl-2,3,4,6-tetra-O-benzyl-α-D-glucopyranosylamide (8) (Reversibility) (400 MHz, Acetone-d6)

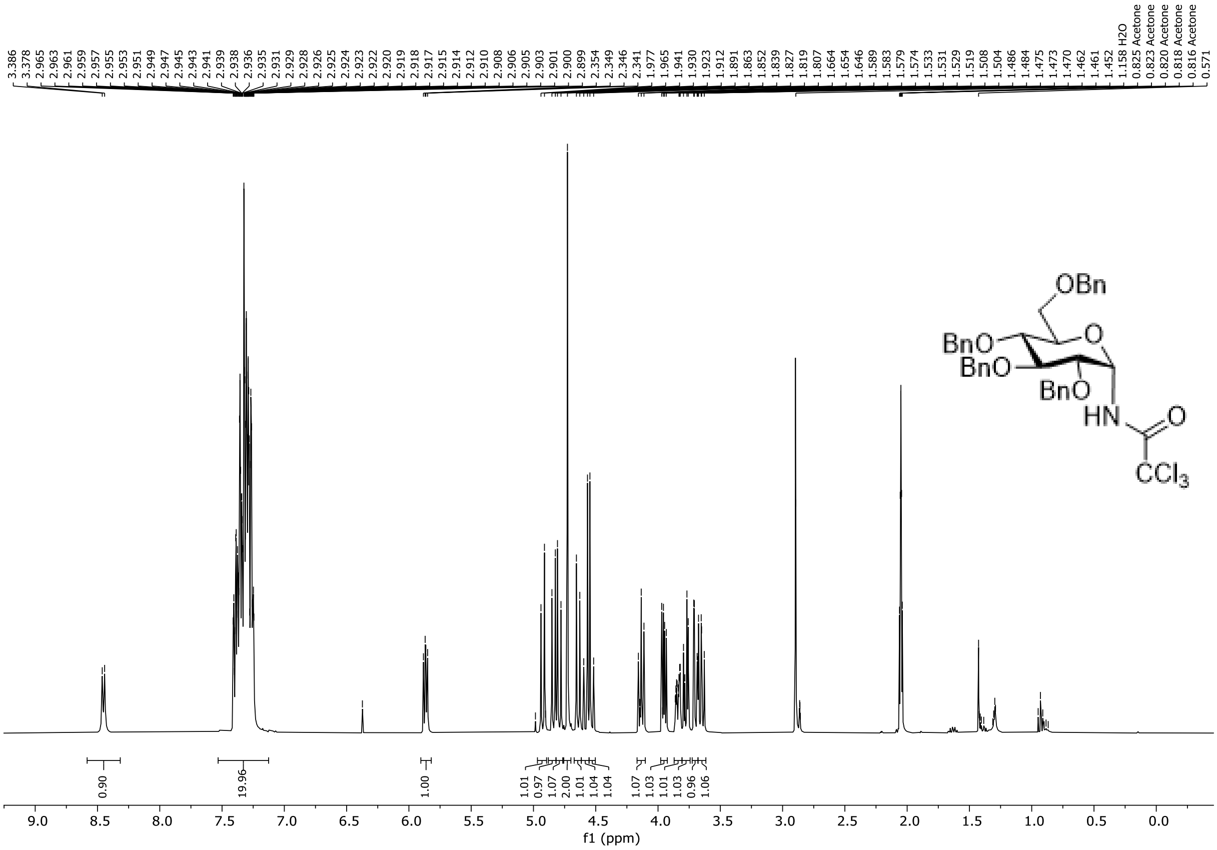

^{13}C NMR of N-trichloroacetyl-2,3,4,6-tetra-O-benzyl-α-D-glucopyranosylamide (8) (Reversibility) (101 MHz, Acetone-d6)

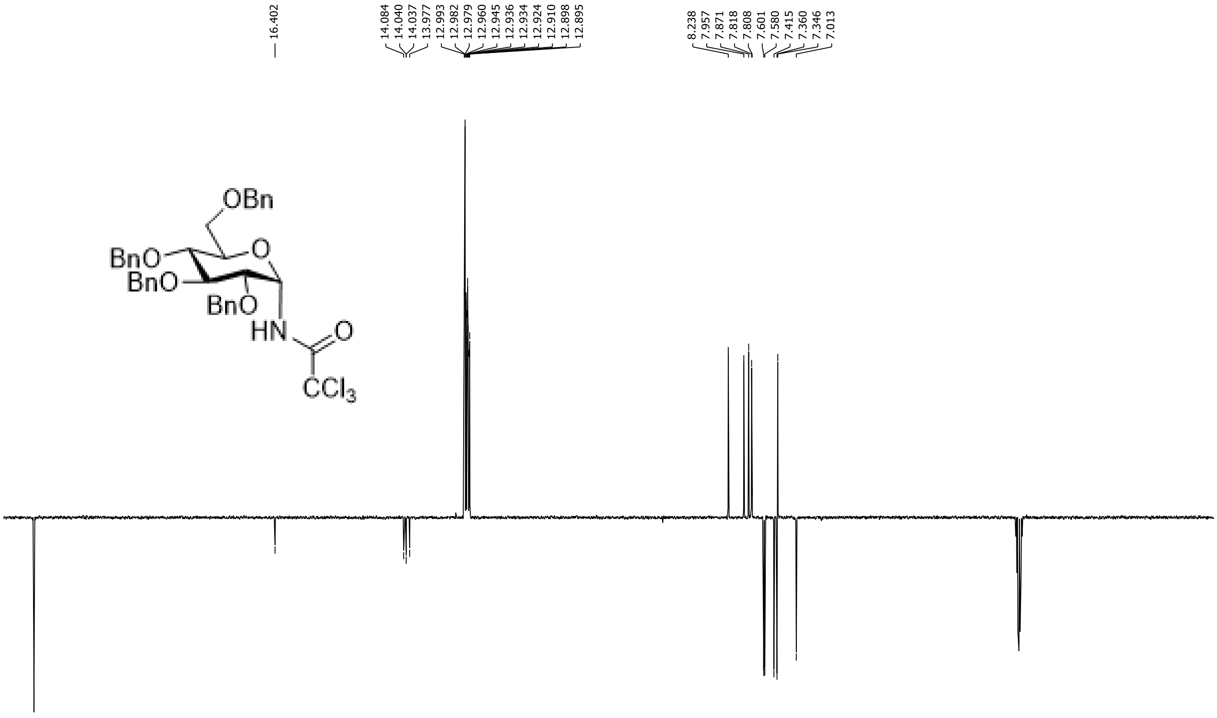

HH COSY of N-trichloroacetyl-2,3,4,6-tetra-O-benzyl-α-D-glucopyranosylamide (8) (Reversibility) (Acetone-d6)

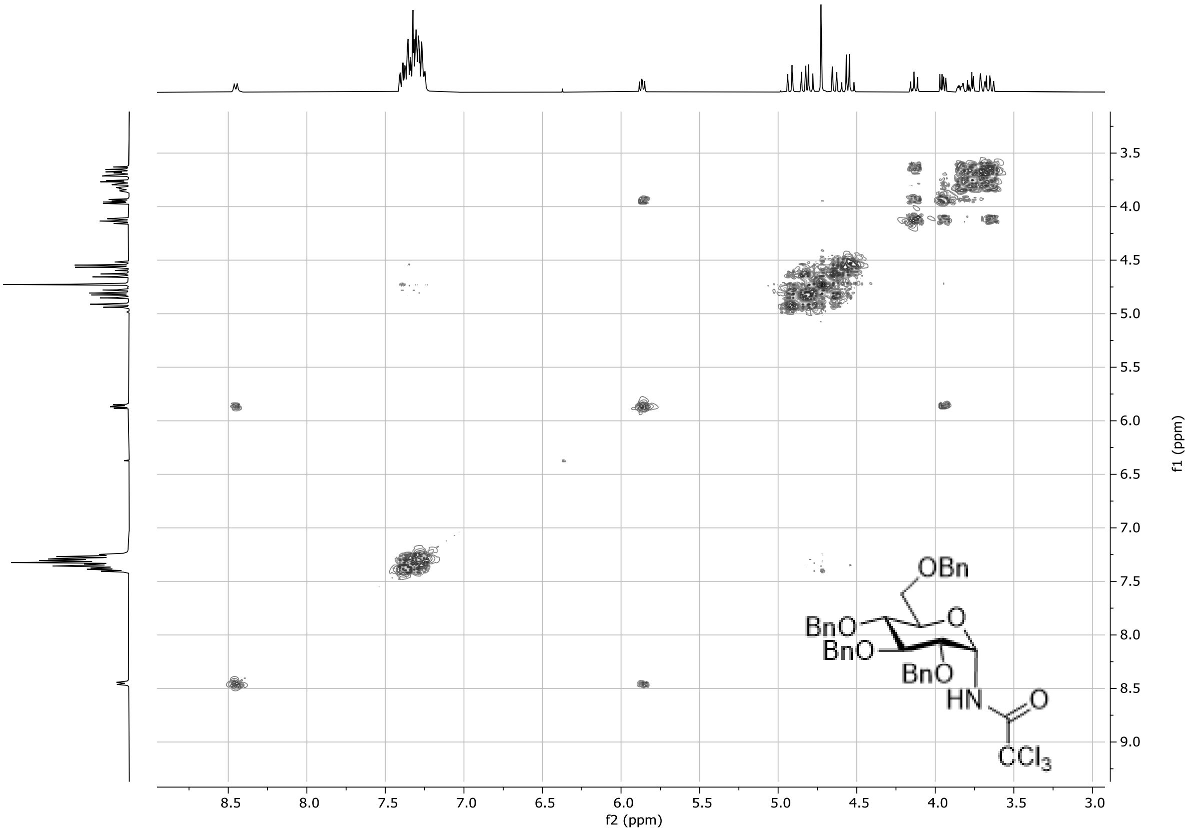

CH HSQC of N-trichloroacetyl-2,3,4,6-tetra-O-benzyl-α-D-glucopyranosylamide (8) (Reversibility) (Acetone-d6)

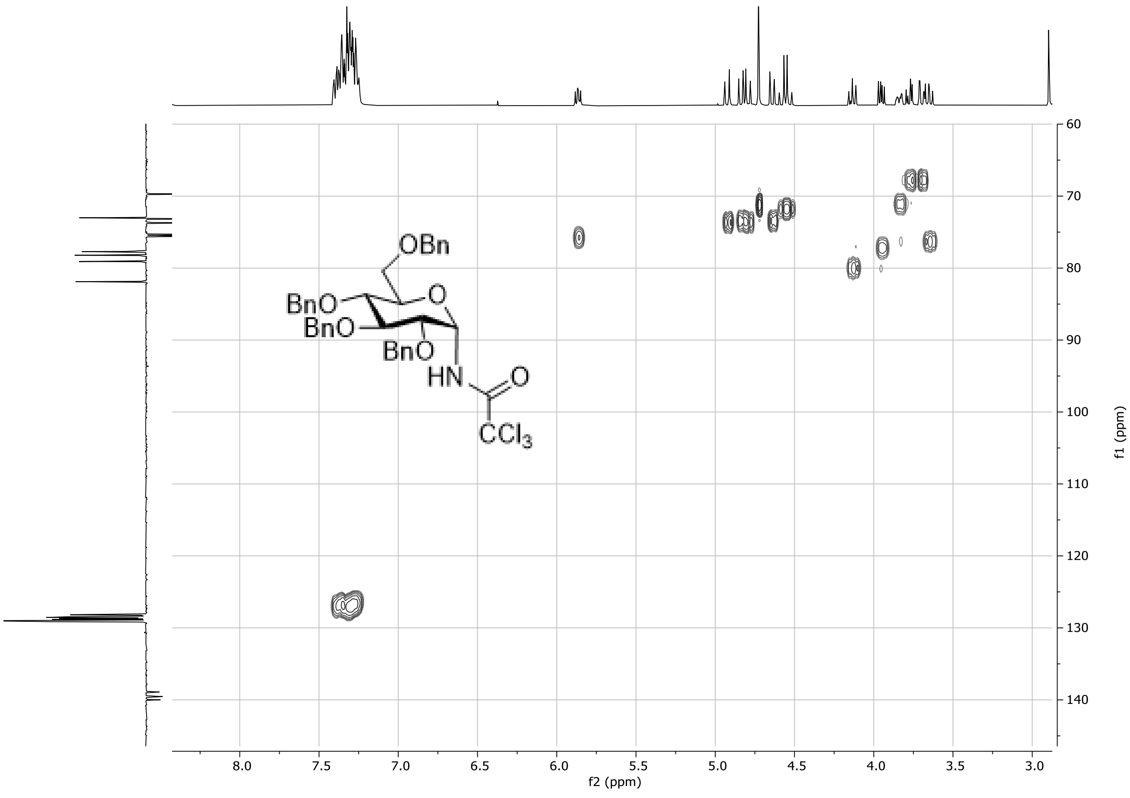

Supplement: Supplementary file 1 — ol3c02196_si_001.pdf [file ol3c02196_si_001.pdf]
